# Supplementary material for: LET-381/FoxF and its target UNC-30/Pitx2 specify and maintain the molecular identity of C. elegans mesodermal glia that regulate motor behavior
Source: EMBO J. 2024 Feb 15;43(6):4. doi: 10.1038/s44318-024-00049-w (PMC10943081; doi:10.1038/s44318-024-00049-w)
Supplement: Supplementary file 1 — Table EV1 [file 44318_2024_49_MOESM1_ESM.docx]

**Table EV1. List of *C. elegans* Transcription Factors with significantly enriched expression in GLR glia (log2Fold Change >1, p-adj< 0.05).** Transcription Factor genes ranked by adjusted p-value. Mutants and/or RNAi of the Top 7 transcription factors of this list were tested for defects in GLR gene expression and morphology; GLR with defects were observed only in *let-381* and *unc-30* mutants.

| **gene** | **Padj** | **log2 fold change** | **base Mean** | **Human Ortholog (if applicable)** |
| --- | --- | --- | --- | --- |
| ***let-381*** | 1.24E-31 | 3.96992369 | 931.237715 | *FOXF* |
| ***fkh-2*** | 3.44E-20 | 5.90100428 | 1448.93045 | *FOXG* |
| ***unc-30*** | 8.68E-20 | 3.57172972 | 9159.67189 | *PITX* |
| ***nhr-56*** | 1.03E-15 | 2.08391644 | 346.31019 |  |
| ***tag-68*** | 1.77E-14 | 2.37306606 | 1899.99157 | *SMAD6/SMAD7* |
| ***duxl-1*** | 1.2E-13 | 1.84520547 | 671.573562 | *ZHX2* |
| ***hlh-1*** | 2.21E-13 | 2.63648844 | 215.079362 | *MYOD/MYF* |
| ***let-607*** | 4.79E-10 | 2.44826798 | 5030.29367 | *CREB3L3* |
| ***fkh-9*** | 3.08E-09 | 1.22261316 | 1566.01925 | *FOXM* |
| ***nhr-88*** | 7E-09 | 1.32612595 | 450.171436 | *HNF4A8* |
| ***nhr-256*** | 0.00000222 | 1.09736897 | 415.553692 | *HNF4A2* |
| ***hmg-1.2*** | 0.00000447 | 1.51298112 | 3021.99495 | *HMGB3* |
| ***aly-1*** | 0.0000305 | 1.2582307 | 191.843676 | *ALYREF* |
| ***nhr-129*** | 0.0000336 | 1.45807642 | 52.4334427 |  |
| ***ceh-88*** | 0.0000344 | 1.32644818 | 213.937354 |  |
| ***nhr-226*** | 0.0000644 | 1.75808608 | 92.766823 |  |
| ***daf-12*** | 0.0000857 | 1.20518418 | 268.995288 | *NR1I1* |
| ***nhr-125*** | 0.0000969 | 2.06163421 | 132.363249 |  |
| ***nhr-32*** | 0.00012025 | 1.05550933 | 229.427897 |  |
| ***elt-7*** | 0.00013735 | 1.06372637 | 306.439622 | *GATA4* |
| ***ceh-79*** | 0.00016477 | 1.02811257 | 142.535705 |  |
| ***nhr-228*** | 0.00019062 | 1.02714318 | 1293.42217 |  |
| ***nhr-109*** | 0.0003689 | 1.31335368 | 96.0212711 |  |
| ***fkh-7*** | 0.00040377 | 1.35297915 | 450.995184 | *FOXP* |
| ***bed-2*** | 0.0004252 | 1.02934756 | 235.831669 |  |
| ***baz-2*** | 0.00051003 | 1.32259797 | 367.659128 | *BAZ2* |
| ***aha-1*** | 0.00052705 | 1.22661892 | 214.116765 | *ARNT* |
| ***K10B3.5*** | 0.00068745 | 1.03067817 | 206.967365 |  |
| ***dve-1*** | 0.00081895 | 1.0946805 | 459.615583 | *SATB* |
| ***hbl-1*** | 0.000858 | 1.42211448 | 58.702432 | *REST* |
| ***nhr-103*** | 0.00181563 | 1.28081207 | 56.5210546 |  |
| ***nhr-154*** | 0.00269862 | 1.41476574 | 82.791574 |  |
| ***nhr-84*** | 0.00329384 | 1.01189361 | 155.992289 |  |
| ***nhr-18*** | 0.00421766 | 1.07177343 | 86.9689312 |  |
| ***nhr-182*** | 0.00436037 | 1.04643881 | 219.289695 |  |
| ***nhr-106*** | 0.00480435 | 1.06180575 | 97.1650622 |  |
| ***lin-28*** | 0.00739187 | 1.13576454 | 222.058392 | *LIN28* |
| ***nhr-47*** | 0.00756044 | 1.12789643 | 598.390972 |  |
| ***ceh-74*** | 0.00798661 | 1.14041521 | 50.9532688 |  |
| ***nhr-59*** | 0.01913946 | 1.11610247 | 88.079953 |  |
