# Supplementary material for: LET-381/FoxF and its target UNC-30/Pitx2 specify and maintain the molecular identity of C. elegans mesodermal glia that regulate motor behavior
Source: EMBO J. 2024 Feb 15;43(6):4. doi: 10.1038/s44318-024-00049-w (PMC10943081; doi:10.1038/s44318-024-00049-w)
Supplement: Supplementary file 2 — Table EV2 [file 44318_2024_49_MOESM2_ESM.docx]

**Table EV2. *let-381* motif-containing minimal promoters from 14 out of 19 genes are sufficient to drive expression of these genes in GLR glia**. Promoters driving GLR glia expression are shown in green. Promoters not driving GLR glia expression are shown in yellow. Three independent extrachromosomal arrays were scored for each promoter. Computationally predicted motifs are not always functional in vivo^1^, which explains why only 20 out of 37 total promoters drive GLR glia expression.

| **GLR enriched gene** | **gfp-based promoter fusion** | **promoter size (bp)** | **# of *let-381* motifs in promoter** | | **Location of motifs from start codon** |
| --- | --- | --- | --- | --- | --- |
| *F41G4.8* | *promoter 1::gfp* | 220 | | 2 | –315, –212 |
|  | *promoter 2::gfp* | 149 | | 1 | –1661 |
| *twk-4* | *twk-4 promoter 1::gfp* | 159 | | 1 | –3634 |
|  | *twk-4 promoter 2::gfp* | 172 | | 1 | –1320 |
|  | *twk-4 promoter 3::gfp* | 167 | | 1 | –384 |
| *ocr-1* | *ocr-1 promoter 1::gfp* | 165 | | 1 | –996 |
|  | *ocr-1 promoter 2::gfp* | 162 | | 1 | –242 |
|  | *ocr-1 promoter 3::gfp* | 169 | | 1 | +924 |
| *twk-9* | *twk-9 promoter 1::gfp* | 214 | | 1 | –62 |
| *mig-6* | *mig-6 promoter 1::gfp* | 168 | | 1 | –1561 |
|  | *mig-6 promoter 2::gfp* | 170 | | 1 | –1149 |
|  | *mig-6 promoter 3::gfp* | 181 | | 1 | –372 |
|  | *mig-6 promoter 4::gfp* | 193 | | 1 | +2128 |
|  | *mig-6 promoter 5::gfp* | 187 | | 1 | +9758 |
| *mig-17* | *mig-17 promoter 1::gfp* | 177 | | 1 | –193 |
|  | *mig-17 promoter 2::gfp* | 162 | | 1 | +273 |
|  | *mig-17 promoter 3::gfp* | 171 | | 2 | +415, + 508 |
| *let-2* | *let-2 promoter 1::gfp* | 174 | | 1 | –2780 |
|  | *let-2 promoter 2::gfp* | 149 | | 2 | –1965, –1944 |
|  | *let-2 promoter 3::gfp* | 214 | | 1 | –500 |
|  | *let-2 promoter 4::gfp* | 177 | | 1 | +611 |
|  | *let-2 promoter 5::gfp* | 184 | | 1 | +1550 |
| *acc-2* | *acc-2 promoter 1::gfp* | 209 | | 2 | –1446, –1389 |
| *oac-7* | *oac-7 promoter 1::rfp* | 180 | | 1 | +2561 |
| *kvs-5* | *kvs-5 promoter 1::rfp* | 200 | | 1 | +867 |
|  | *kvs-5 promoter 2::rfp* | 152 | | 1 | +3138 |
| *lbp-1* | *Ibp-1 promoter 1::rfp* | 158 | | 1 | –1090 |
|  | *Ibp-1 promoter 2::rfp* | 163 | | 1 | +491 |
| *adt-3* | *adt-3 promoter 1::rfp* | 181 | | 1 | +4517 |
| *R03E9.2* | *R03E9.2 promoter 1::rfp* | 172 | | 2 | –5259, –5196 |
|  | *R03E9.2 promoter 2::rfp* | 182 | | 1 | –5050 |
|  | *R03E9.2 promoter 3::rfp* | 158 | | 1 | –3428 |
|  | *R03E9.2 promoter 4::rfp* | 170 | | 1 | –2180 |
| *F49B2.6* | *F49B2.6 promoter 1::rfp* | 153 | | 1 | –1459 |
|  | *F49B2.6 promoter 2::rfp* | 154 | | 2 | –198, –154 |
| *nta-1* | *nta-1 promoter 1::rfp* | 156 | | 1 | –69 |
|  | *nta-1 promoter 2::rfp* | 154 | | 1 | +145 |
| *T14B4.9* | *T14B4.9 promoter 1::gfp* | 172 | | 1 | –1647 |
|  | *T14B4.9 promoter 2::gfp* | 254 | | 2 | –393, –279 |
|  | *T14B4.9 promoter 3::gfp* | 171 | | 1 | +210 |
| *tbc-12* | *tbc-12 promoter 1::gfp* | 172 | | 2 | +1481, +1545 |
|  | *tbc-12 promoter 2::gfp* | 155 | | 1 | +1669 |
|  | *tbc-12 promoter 3::gfp* | 232 | | 2 | +2086, +2208 |
|  | *tbc-12 promoter 4::gfp* | 232 | | 1 | +2918 |
| *pgp-4* | *pgp-4 promoter 1::rfp* | 187 | | 1 | –325 |
|  | *pgp-4 promoter 2::rfp* | 157 | | 1 | +558 |
| *haf-7* | *haf-7 promoter 1::rfp* | 178 | | 2 | –1468, –1370 |
|  | *haf-7 promoter 2::rfp* | 204 | | 1 | +2134 |

1. Slattery, M., Zhou, T., Yang, L., Dantas Machado, A.C., Gordan, R., and Rohs, R. (2014). Absence of a simple code: how transcription factors read the genome. Trends Biochem Sci *39*, 381-399. 10.1016/j.tibs.2014.07.002.
