# Supplementary material for: LET-381/FoxF and its target UNC-30/Pitx2 specify and maintain the molecular identity of C. elegans mesodermal glia that regulate motor behavior
Source: EMBO J. 2024 Feb 15;43(6):4. doi: 10.1038/s44318-024-00049-w (PMC10943081; doi:10.1038/s44318-024-00049-w)
Supplement: Supplementary file 3 — Appendix [file 44318_2024_49_MOESM3_ESM.pdf]

Appendix

LET-381/FoxF and its target UNC-30/Pitx2 specify and maintain the molecular identity of *C. elegans* mesodermal glia that regulate motor behavior

Authors: Nikolaos Stefanakis, Jessica Jiang, Yupu Liang, Shai Shaham

*Correspondence to shaham@rockefeller.edu

Table of Contents:

Appendix Figure S1 p. 2

Appendix Figure S2 p. 4

Appendix Figure S3 p. 6

Appendix Figure S4 p. 7

Appendix Figure S5 p. 8

Appendix Table S1 p. 11

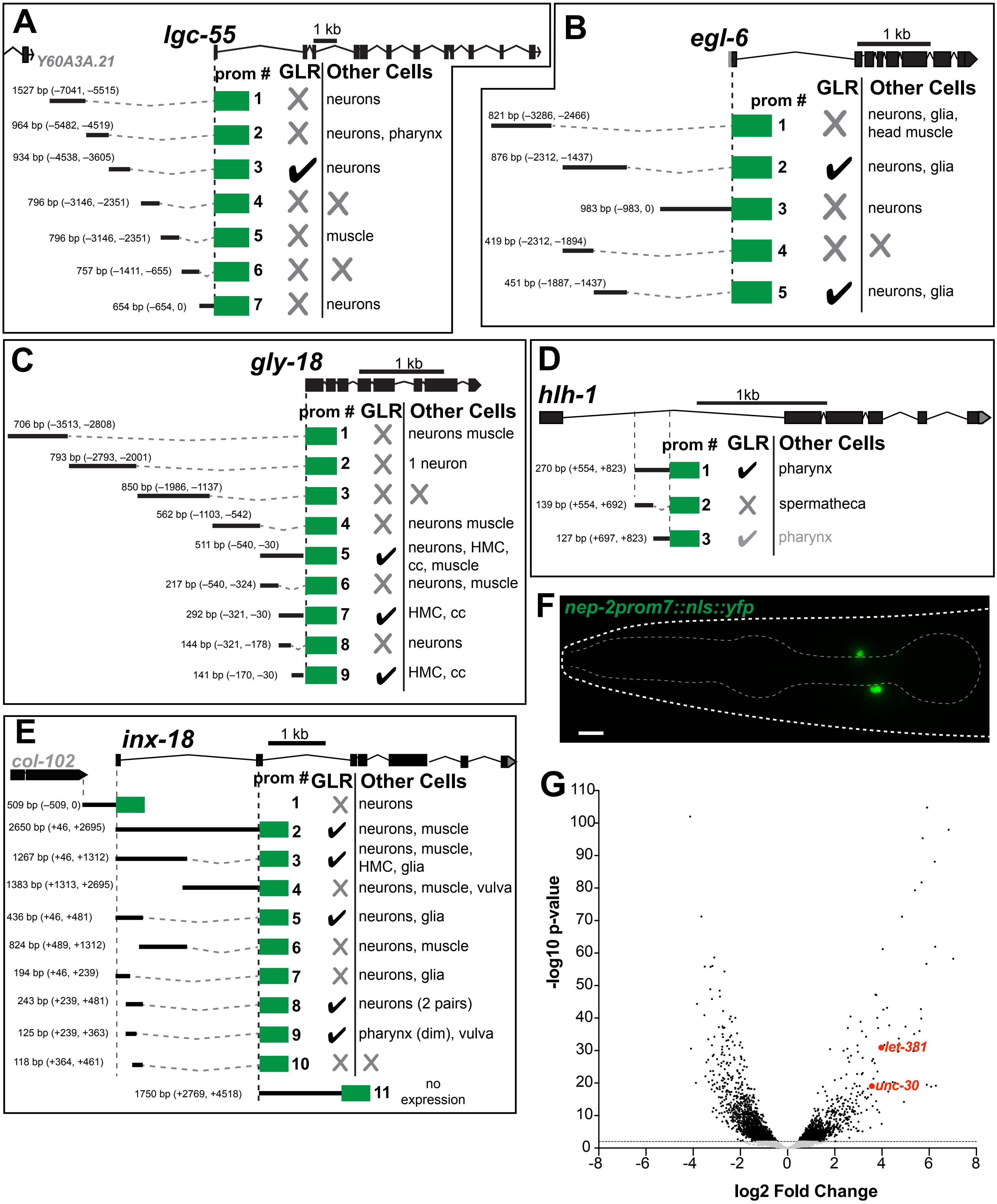
A
lgc-55
1 kb
Y60A3A.21
prom # GLR Other Cells
1527 bp (–7041, –5515) 1 neurons
964 bp (–5482, –4519) 2 neurons, pharynx
934 bp (–4538, –3605) 3 neurons
796 bp (–3146, –2351) 4
796 bp (–3146, –2351) 5 muscle
757 bp (–1411, –655) 6
654 bp (–654, 0) 7 neurons
B
egl-6
1 kb
prom # GLR Other Cells
821 bp (–3286, –2466) 1 neurons, glia, head muscle
876 bp (–2312, –1437) 2 neurons, glia
983 bp (–983, 0) 3 neurons
419 bp (–2312, –1894) 4
451 bp (–1887, –1437) 5 neurons, glia
C
gly-18
1 kb
prom # GLR Other Cells
706 bp (–3513, –2808) 1 neurons muscle
793 bp (–2793, –2001) 2 1 neuron
850 bp (–1986, –1137) 3
562 bp (–1103, –542) 4 neurons muscle
511 bp (–540, –30) 5 neurons, HMC, cc, muscle
217 bp (–540, –324) 6 neurons, muscle
292 bp (–321, –30) 7 HMC, cc
144 bp (–321, –178) 8 neurons
141 bp (–170, –30) 9 HMC, cc
D
hlh-1
1kb
prom # GLR Other Cells
270 bp (+554, +823) 1 pharynx
139 bp (+554, +692) 2 spermatheca
127 bp (+697, +823) 3 pharynx
E
inx-18
1 kb
col-102
prom # GLR Other Cells
509 bp (–509, 0) 1 neurons
2650 bp (+46, +2695) 2 neurons, muscle
1267 bp (+46, +1312) 3 neurons, muscle, HMC, glia
1383 bp (+1313, +2695) 4 neurons, muscle, vulva
436 bp (+46, +481) 5 neurons, glia
824 bp (+489, +1312) 6 neurons, muscle
194 bp (+46, +239) 7 neurons, glia
243 bp (+239, +481) 8 neurons (2 pairs)
125 bp (+239, +363) 9 pharynx (dim), vulva
118 bp (+364, +461) 10
1750 bp (+2769, +4518) 11 no expression
F
nep-2prom7::nls::yfp
G
-log10 p-value
log2 Fold Change
let-381
unc-30

Appendix Figure S1. Analysis of cis-regulatory regions of GLR glia-expressed genes.

(A) – (E) Cis-regulatory dissection analysis for the genes *lgc-55* (A), *egl-6* (B), *gly-18* (C), *hlh-1* (F) and *inx-18* (E).

(F) A stable transgenic strain expressing nuclear YFP under the GLR glia-specific *nep-2prom7* was used to isolate GLR glia for the transcriptome analysis. Anterior is left, dorsal is up and scale bar is 10 μm.

(G) Volcano plot show significantly enriched and depleted genes (p-value >0.05) among all GLR glia expressed genes (>50 reads). *let-381* and *unc-30*, the two transcription factor genes we found to have important roles in GLR glia development, are among the most highly enriched genes.

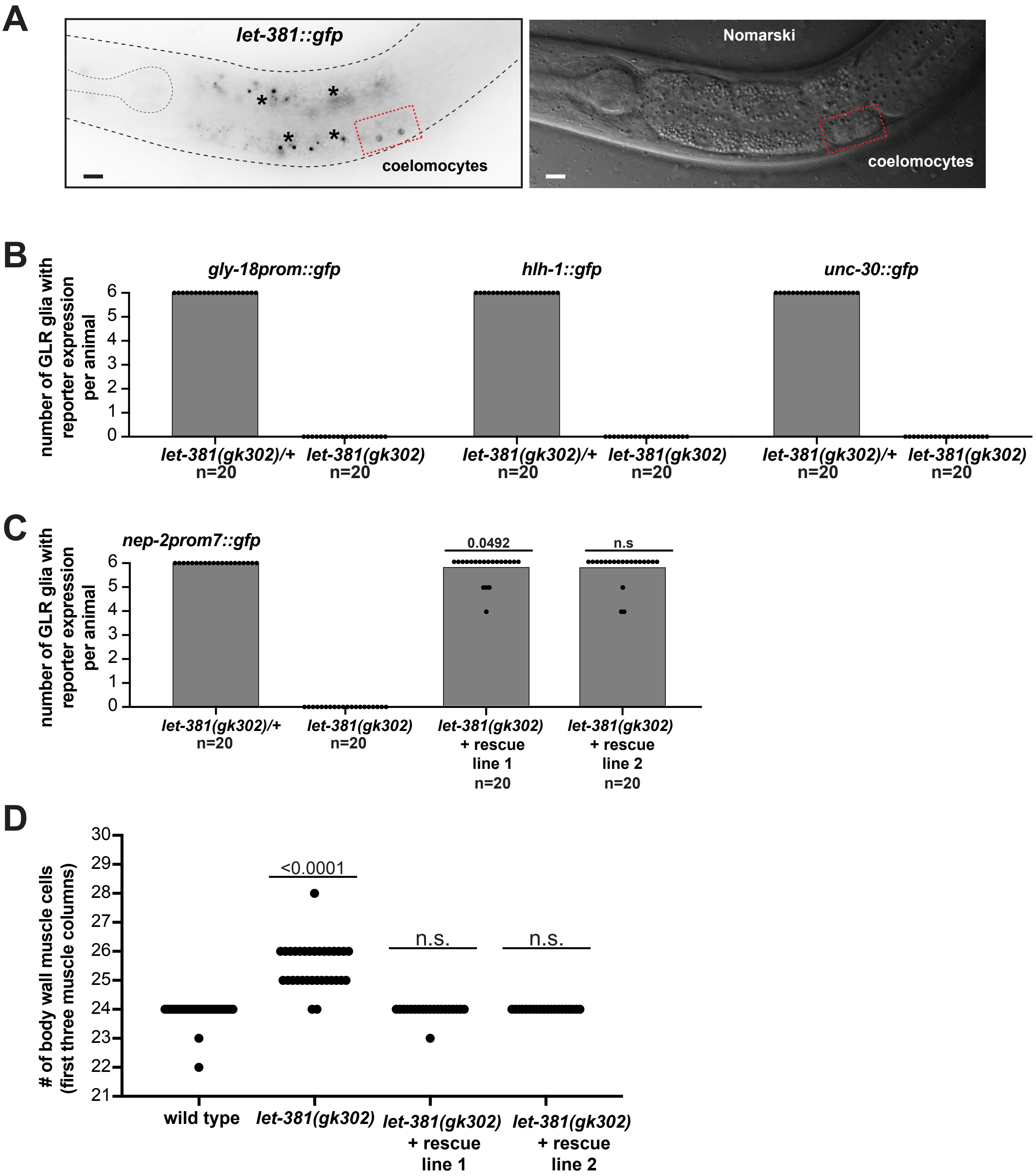
A
let-381::gfp
coelomocytes
Nomarski
coelomocytes
B
gly-18prom::gfp
hlh-1::gfp
unc-30::gfp
number of GLR glia with reporter expression per animal
let-381(gk302)/+ n=20
let-381(gk302) n=20
C
nep-2prom7::gfp
0.0492
n.s
number of GLR glia with reporter expression per animal
let-381(gk302)/+ n=20
let-381(gk302) n=20
let-381(gk302) + rescue line 1 n=20
let-381(gk302) + rescue line 2 n=20
D
<0.0001
n.s.
n.s.
# of body wall muscle cells (first three muscle columns)
wild type
let-381(gk302)
let-381(gk302) + rescue line 1
let-381(gk302) + rescue line 2

Appendix Figure S2. *let-381/FoxF* is required for GLR glia fate specification.

(A) *let-381::gfp* is expressed in the coelomocytes (red dashed box). Asterisks denote non-specific gut granule fluorescence. Nomarski image on the right panel.

(B) Number of GLR glia expressing *gly-18prom::gfp*, *hlh-1::gfp* and *unc-30::gfp* expression in wild type and homozygous *let-381(gk302)* mutants. Expression of all genes tested is absent from GLR cells in *let-381* homozygous null mutants.

(C), (D) Animals transgenic (2 independent lines) for the fosmid genomic clone WRM069bF08 carrying wild-type *let-381* have (B) 6 GLR glia and (C) no extra head muscle cells.

Data information: Anterior is left, dorsal is up and scale bars are 10μm for all animal images. Un-paired t-test used for statistical analysis in (B) and (C).

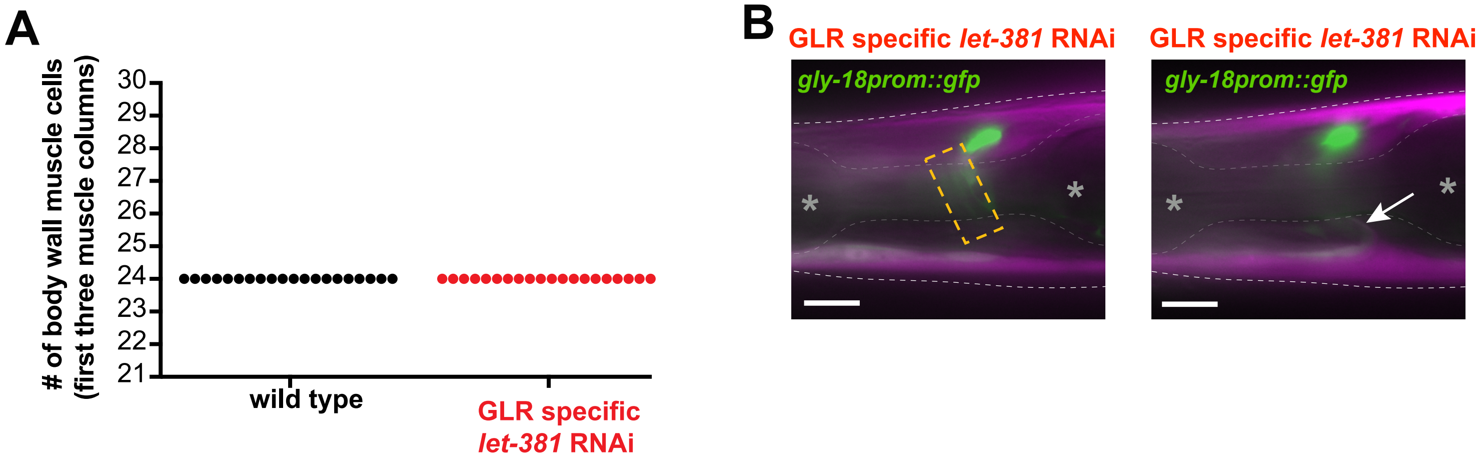

Appendix Figure S3. Postembryonic *let-381* knockdown does not affect GLR fate specification.

(A) Animals carying the GLR glia-specific *let-381* RNAi arrays do not have extra head muscle cells.

(B) In addition, *let-381* RNAi does not cause anteriorly displaced Nerve Ring. Yellow dashed box outlines the Nerve Ring (green) on the left image and arrow on the right image shows a head muscle arm extending into the Nerve Ring. As evidenced, the Nerve Ring is located in its normal position between the two pharyngeal bulbs (grey asterisks).

Data information: Anterior is left, dorsal is up and scale bars are 10μm for all animal images.

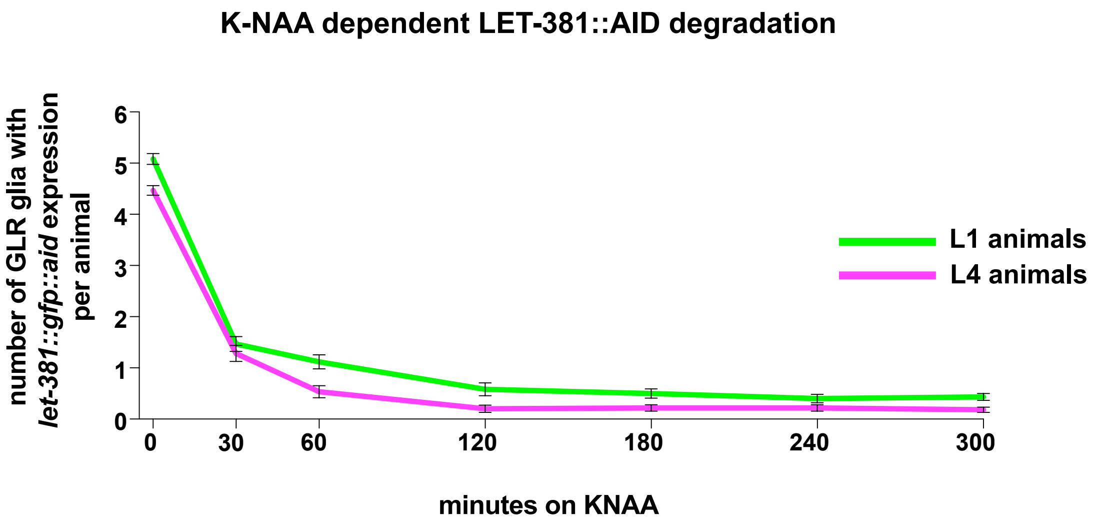

Appendix Figure S4. LET-381::GFP::AID is rapidly degraded in the presence of the K-NAA auxin analog.

Temporal analysis of *let-381::gfp::aid* degradation in presence of the auxin analog K-NAA. *let-381::gfp* expression is lost in GLR glia within 2 hours of exposure to K-NAA in both L1 and L4 stage animals.

Data information: n=60 for each time point. Error bars show standard error of the mean.

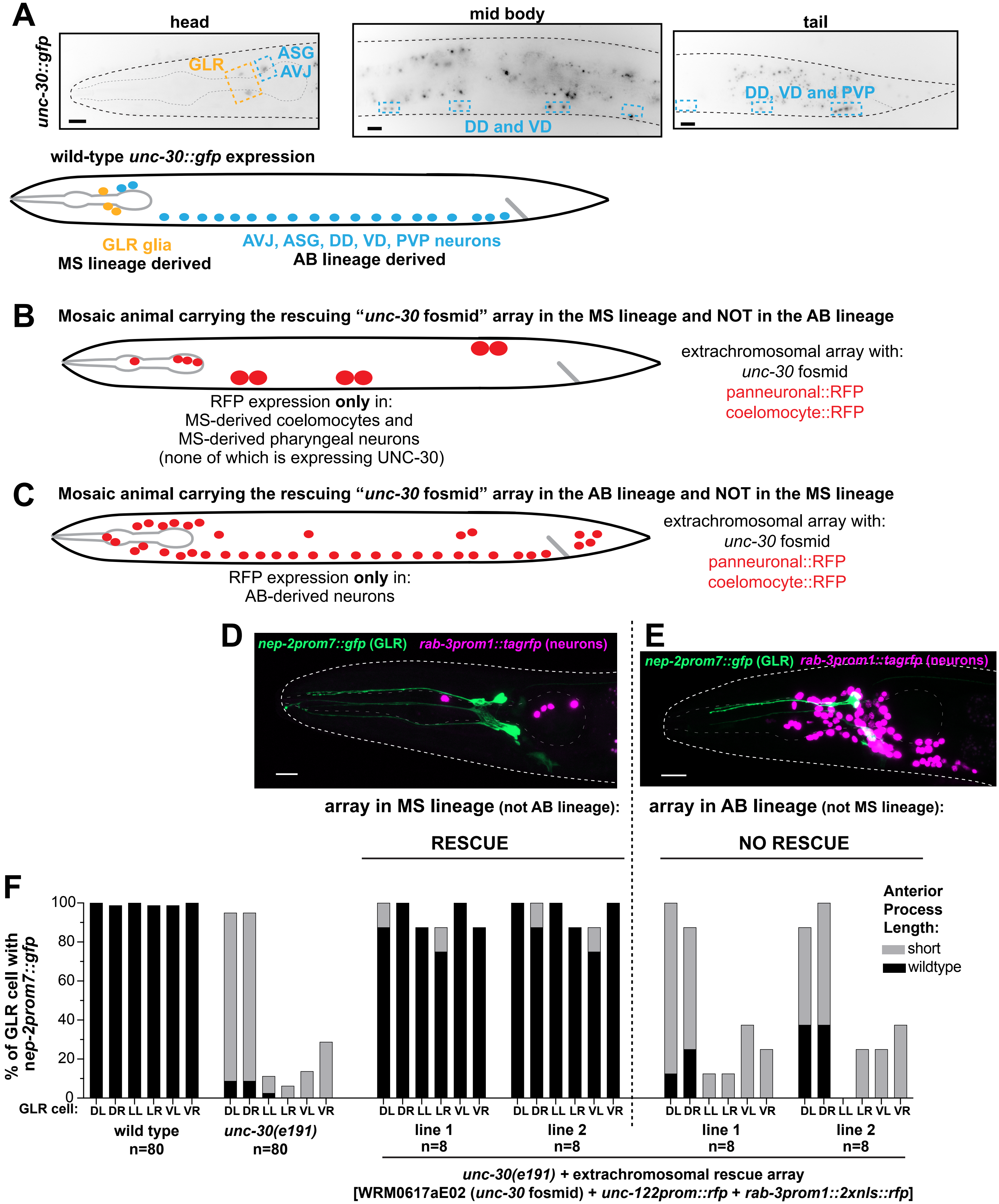
A
unc-30::gfp
head
mid body
tail
GLR
ASG
AVJ
DD and VD
DD, VD and PVP
wild-type unc-30::gfp expression
GLR glia
MS lineage derived
AVJ, ASG, DD, VD, PVP neurons
AB lineage derived
B
Mosaic animal carrying the rescuing "unc-30 fosmid" array in the MS lineage and NOT in the AB lineage
RFP expression only in:
MS-derived coelomocytes and
MS-derived pharyngeal neurons
(none of which is expressing UNC-30)
extrachromosomal array with:
unc-30 fosmid
panneuronal::RFP
coelomocyte::RFP
C
Mosaic animal carrying the rescuing "unc-30 fosmid" array in the AB lineage and NOT in the MS lineage
RFP expression only in:
AB-derived neurons
extrachromosomal array with:
unc-30 fosmid
panneuronal::RFP
coelomocyte::RFP
D
nep-2prom7::gfp (GLR) rab-3prom1::tagrfp (neurons)
E
nep-2prom7::gfp (GLR) rab-3prom1::tagrfp (neurons)
array in MS lineage (not AB lineage):
array in AB lineage (not MS lineage):
RESCUE
NO RESCUE
F
% of GLR cell with nep-2prom7::gfp
100
80
60
40
20
0
GLR cell:
DL DR LL LR VL VR
wild type n=80
unc-30(e191) n=80
line 1 n=8
line 2 n=8
line 1 n=8
line 2 n=8
Anterior Process Length:
short
wildtype
unc-30(e191) + extrachromosomal rescue array
[WRM0617aE02 (unc-30 fosmid) + unc-122prom::rfp + rab-3prom1::2xnls::rfp]

Appendix Figure S5. **UNC-30 acts cell autonomously to control GLR glia gene expression and morphology.**

(A) Fluorescence images (top) and schematic representation (bottom) of endogenous *unc-30::gfp* expression in wild-type animals. *unc-30::gfp* is expressed in the MS-lineage derived GLR glia (yellow dashed boxes), and in the ASG and AVJ head neurons, DD and VD Ventral Nerve Cord neurons and PVP neurons of the preanal ganglion, all deriving from the neuroectodermal-like lineage of the blast cell AB. We performed mosaic analysis to determine whether *unc-30* acts cell autonomously to control GLR glia gene expression and morphology. To this end we used unstable extrachromosomal arrays carrying a) wild type copies of the *unc-30* locus (*unc-30* fosmid WRM0617aE02) that can rescue the *unc-30(e191)* phenotype (Fig. EV3A) and b) *rfp* based panneuronal (*rab-3prom1::2xnls::tagrfp*) and coelomocyte (*unc-122prom::rfp*) transgenes to follow the array.

(B) Rare mosaic animals carrying the extrachromosomal array in the MS-lineage and NOT in the AB-lineage would express RFP only in the coelomocytes and the six MS-derived pharyngeal neurons I3, I4, I6, M1, M4 and M5, easily distinguished by their stereotypic position.

(C) Rare mosaic animals carrying the extrachromosomal array in the AB-lineage and NOT the MS lineage would have broad neuronal expression but no expression in the six MS-derived neurons mentioned above and no expression in coelomocytes.

(D) Fluorescence image of animal carrying the rescuing array in the MS lineage and not the AB lineage. RFP expression is in the MS-derived pharyngeal neurons M4, I4, I6, M1

and M5. GFP expression in GLR glia (green) shows rescue of the *unc-30(e191)* phenotypes.

(E) Fluorescence image of animal carrying the rescuing array in the AB lineage and not the MS lineage. RFP is broadly expressed in AB-derived neurons. GFP expression in GLR glia (green) does not show rescue of the *unc-30(e191)* phenotypes.

(F) Quantification of the mosaic rescue analysis. Presence of the array in the MS lineage and not the AB lineage is sufficient to rescue the *unc-30(e191)* null mutant phenotype on GLR gene expression and morphology.

Data information: Anterior is left, dorsal is up and scale bars are 10μm for all animal images.

Appendix Table S1. List of primers used in this study.

**TRANSGENIC REPORTERS MADE BY PCR FUSION**			
	**primer C**	**Primer D**	**Primer D***
gfp/rfp amplicon	agcttgcatgcctgcaggtcg	aagggcccgtacggccgacta	caagaaaaacgccgtcctcg
	**Primer A**	**Primer A***	**Primer B:** B primers start with CTCTAGAGTCGACCT GCAGGCATGCAAGC (homology with gfp/rfp amplicon), followed by the gene specific sequence listed below
nep-2 prom1	ggacgatgctcttcgcaaag	gctcttcgcaaagtggctcc	atcgggaggcgtccgaccgt
nep-2 prom2	gacgtcagcgcgttcttaac	cagcgcgttcttaaccatgc	atcgggaggcgtccgaccgt
nep-2 prom3	ggacgatgctcttcgcaaag	gctcttcgcaaagtggctcc	gtagatcaaaccgtaatggg
nep-2 prom4	ggacgatgctcttcgcaaag	gctcttcgcaaagtggctcc	cggagtcagaagttatacaa
nep-2 prom5	gactccgcccattccgattc	cccattccgattcccacttg	gtagatcaaaccgtaatggg
nep-2 prom6	cgacctcatcatattttaagtg	cgacctcatcatattttaagtg	gtagatcaaaccgtaatggg
nep-2 prom7	gactccgcccattccgattc	cccattccgattcccacttg	cttaaaatatgatgaggtcg
lgc-55 prom1	ccagcctaacttgctccgtt	ccatgccaatttagcgcatc	ggtctataacaagggtgtcg
lgc-55 prom2	cataggcgtgttttaaagca	gcgctactccaccttgaaga	ggcgtaggctttggcattgg
lgc-55 prom3	ccaatgccaaagcctacgcc	gcctaagcctaagcctaagt	ccaccagtactctttcaatg
lgc-55 prom4	cctggtgggtactataaagc	gggtactataaagcggggca	gtaggcgccaaaacgcttgc
lgc-55 prom5	ctacgtggtagcccaaaaca	gttagtggtactgtgcacaag	cctctcatcttccgagagac
lgc-55 prom6	gcaccacctcaagatttatc	cattggtttcgctcttgtttg	cgccacgaaaaacttgttga
lgc-55 prom7	caacaagtttttcgtggcg	ggacccaaaattctctaccc	ccatttcatttcgacatcta
egl-6 prom1	ccgcaattttttggtggtg	gaacatttcaccggaatctc	agccgagttaaagtctaaag
egl-6 prom2	cctagtggtgcgttccttcc	ctttccttccaggaatttcg	cagctttctatgttgccagc
egl-6 prom3	cagctgaattgagctaccac	cgttttcttgtcgttttgctc	tgctgaaaagctgtcattgt
egl-6 prom4	cctagtggtgcgttccttcc	ctttccttccaggaatttcg	gaaacctttcggtctcacac
egl-6 prom5	ggtttcaagttagagctact	gagctactactttctattcc	cagctttctatgttgccagc
gly-18 prom1	cttgtcaaacatgctaactg	gagccaactagctgtttctt	gaagtcgcggaagctactta
gly-18 prom2	ctaagtagcttccgcgacttc	ctttacttacctacagtgtcc	cacagttacaaggtgcaccc
gly-18 prom3	ggtgcaccttgtaactgtgt	ctcgtagcttgttgaacgag	ctatactaatgccaatattg
gly-18 prom4	gccactccactatattttcg	ctgtgttcacacaatgactgc	caatactttgacagctgtct
gly-18 prom5	gagacagctgtcaaagtattg	gtatcacttgtgaaaagccc	cttgatacgttttaacacag
gly-18 prom6	gagacagctgtcaaagtattg	gtatcacttgtgaaaagccc	agacatgagagaaattcagg
gly-18 prom7	ctcatgtctaacccttgtgg	cccttgtggaattttgtcaa	cttgatacgttttaacacag
gly-18 prom8	ctcatgtctaacccttgtgg	cccttgtggaattttgtcaa	gaattattggaaaactggga
gly-18 prom9	caattgaagaactccgcgac	caattgaagaactccgcgac	cttgatacgttttaacacag
hlh-1 prom1	ggttataatgagcaccagatg	ggttataatgagcaccagatg	caacgggttaacccagtcta
hlh-1 prom2	ggttataatgagcaccagatg	ggttataatgagcaccagatg	cataaattctcttctcccct
hlh-1 prom3	gaaatgggtcatgggaatag	gggtcatgggaatagtaaag	caacgggttaacccagtcta
inx-18 prom1	ggagaagcttccccagccta	caaccggacagagcagctac	tcgcgaacgtcttcaggcttc
inx-18 prom2	ggaatgcactttctggcctc	ctaaggtttcttgaaccaag	cgtcaactctggaaattttc
inx-18 prom3	ggaatgcactttctggcctc	ctaaggtttcttgaaccaag	ccgtatacacattacatgtg
inx-18 prom4	cacatgtaatgtgtatacgg	ggagagcactcatatcatct	cgtcaactctggaaattttc

inx-18 prom5	ggaatgcactttctggcctc	ctaaggtttcttgaaccaag	ctacaaggcggcctactttg
inx-18 prom6	gcacgcaggcagacatgttc	ggcagacatgttcgtacctg	ccgtatacacattacatgtg
inx-18 prom7	ggaatgcactttctggcctc	ctaaggtttcttgaaccaag	cttgaatagggagatggaac
inx-18 prom8	gtcccacattacactttcgc	gtcccacattacactttcgc	ctacaaggcggcctactttg
inx-18 prom9	gtcccacattacactttcgc	gtcccacattacactttcgc	tttattagtatttgatggtc
inx-18 prom10	gtaaacataatgcgattgtag	gtaaacataatgcgattgtag	ctacaaggcggcctactttg
inx-18 prom11	catgtttgctgttttggtgtc	caaacaatatggttcgtagg	ccattgccctggtaaattgt
unc-30 prom1	cgtctccaactgtgttctgt	tctgttgtcttttacctacg	caaaagctgcaagatcttgg
unc-30 prom2	ccaagatcttgcagcttttg	gaaagtcttcttggcgacatg	cttgttttggagttacagcc
unc-30 prom3	cgatagaagtgagcttggtc	ccaaaacaaggtgtctgatc	gaacgctcttttccagttttc
unc-30 prom4	ctgctgaaaacttagttatg	catatgcagagagaggcttag	cacttgggtgtaacttctca
unc-30 prom5	tgagaagttacacccaagtg	ggaacacgttgaccgtgaga	cccttatcatcaccagatgt
unc-30 prom6	gtaaacagtttgcgcaattgc	ggtgatgataagggtagatg	gtgttgatcttttgccggag
unc-30 prom7	cggccacactgactgctatac	gcagcagtcgcataatcggt	ctgaaataaaagaaatggaag
unc-30 prom8	gcagtcaggtaagcagaagg	gtaagcagaaggcaggcatc	gagtcgtctagctgaaaatgg
unc-30 prom9	ctagtcgattttcgactgct	ccaacgtgagttttacggat	ccacaggatgctgtcaaacc
unc-30 prom10	ggtttgacagcatcctgtgg	ggtttctcctactccaatcc	gaatgcagcttgggctctgg
pll-1 prom1	cttgtctgcgtgcagtaattc	acaatttgccggcttgccga	cacattgtgtttggttttcc
hot-5 prom2	gacacactactggcgttact	cgaagtacaagttaagcttc	ctgacggaaactcttactca
glb-26 prom1	gactgtggagacgatcgtac	gttcgaagatctgcacgaag	ctgggaatgagcacacgaaa
T28A11.3 prom1	ctatcaaatgggttcttctc	*cgttggactctacaccgaga*	*ctgacagaaaagaataggg*
F28H7.2 prom1	gcgatatattcccacctctg	*gctccagaacgagacatttg*	*cgaatcaaatagttgacgag*
ZC317.2 prom1	ctccactggatgggttacgc	*cagtgtgaagctccagcttc*	*ctggaaaatcatttgatagg*
F35D2.3 prom1	cgtctcccgaaatttccaga	*cctgagatgcaccgtatgtc*	*ctgtaagaagccgccattag*
F13D12.10 prom1	cccattttcagaacgatctag	*gaaaaccgtcgacctggttc*	*tgtagctgctcgtgttcggc*
mnp-1 prom3	cgtatccatgtggtcaactg	*gtgagcttctactacaatca*	*acgcagatggtttcgcctg*
ncam-1 prom2	ggttttacggcggcatatgt	gcggcatatgttattctagc	caaacacatgctcttcgcat
unc-54 prom3	cgactccaaagtaggttagt	gagacatgtttcatctgaag	cgcttctttcaaatggtttc
F41G4.8 prom1	gacatgtgaacttaggtttcc	*gatcctgatttccgctccaa*	*gtgaactcaactcaaactttg*

F41G4.8 prom2	gagatcgattagacacagtg	*ggtaagaaaagggtttcgag*	*ccagccgctgcaatactgag*
twk-4 prom1	ctcaccactaggatctgttag	*gagacatactttcctgttcgg*	*ccgcctatatcttggtttttc*
twk-4 prom2	gaaagtataggtgagggagg	*ctgagggcaaggaggagaag*	*caggtcgaagtattttacagg*
twk-4 prom3	ccgaagagaaagggttcaatc	gttttgtgaatcccaagtgc	*gggtagcagtagaatggggg*
ocr-1 prom1	gcgttgacagcaactttcgg	gcatggacaagttcaaaggg	*cgttctggacgttgctttccc*
ocr-1 prom2	ctgcagtttcacgacacaac	ggatctccatgaacacccttc	*cagattctcggattgtctttc*
ocr-1 prom3	gagagtgagaggattttccg	ccacgaacttgatgaatgac	*caacgctccataccatttctc*
twk-9 prom1	cattcacatgttttggcaag	ggcagttcatattttggagg	*cgggaatgtggaagctgca*
mig-6 prom1	cagatgactcttcccagtttc	gctatctcttccgtcaaatc	*cagaaactttttgtgtgcag*
mig-6 prom2	gaaatctgatccgccgtggtc	ctttccgaatgttgttccgg	*gaagatctagatgatctcac*
mig-6 prom3	gaaccgaactcgatcgttttc	ctgcgtctcttttaactctc	*gaatggcgcacactttggctc*
mig-6 prom4	ctgcggagatcaaggacagc	gcagacttgcaatcggtaag	*cacttctctgagcaagccgac*
mig-6 prom5	gttcacatttatctccagtc	gaatacgatgcacgtcgctc	*ctcattgatacccctttcaag*
mig-17 prom1	gaggcttctttcttacaacac	gtggagagaatggatggggtg	*cccatctcttgacccatggtc*
mig-17 prom2	gaggaaagttcaggatggac	gctcagtagaagcttagtgg	*gacatttttcggtcgatttcc*
mig-17 prom3	gctcagtagaagcttagtgg	ggaaatcgaccgaaaaatgtc	*ctcctaacagcccaaaaaag*
let-2 prom1	caatagttacggggcacacc	ggtggcatgaagaatgattg	*gaaaacttcttgacttgcac*
let-2 prom2	ccctactctctatgttttgc	gcactttgtgcaacacgaaag	*gatgaatcgcacatatgtggc*
let-2 prom3	gaaggttgacattcctaggc	ctgaacttttgacctttcag	*ggtcagtagattgatcgggtg*
let-2 prom4	gcctaaaaagtgaacgtttg	catgcagagattgctctaacc	*gatattggggagagagttaac*
let-2 prom5	gaatcaagggagatcgtgga	ggttgcaacggaactgacgg	*ctagacttccctatcacaac*
acc-2 prom1	ccatacttttggcccaccctg	gctttggtgtttagcctattc	*cgaagaaaaggaatgtgcag*
oac-7 prom1	ggacgtcaagaaagccattg	caagaagtaccaaactgagg	*catttgaatatccgtggcgg*
kvs-5 prom1	cctcatcctttctatgcttg	cgggtttcttcttgcaaaaac	cttcctacgcgcctacctaac
kvs-5 prom2	gtaatataggaacggcatctc	gaagaaacgcctcttgttttc	*ccgcaaagtatcaaacttcac*
lbp-1 prom1	gtcatggtgtgtacttcgttc	caatggaatcacgtgccgtc	*ggcagagcaagtctaaaggg*
lbp-1 prom2	cgcttttgcaagttttcaag	gagacgccaaggaagagacc	*ggagtcgtttgtagaatcggc*
adt-3 prom1	cgccaccaatgccacatgttc	ccaagttttagccccccttcc	*ggacaaattcagtaggacatc*
R03E9.2 prom1	gaaatgaaagggaatacccgg	cgaaaatgcgaatgtgcagac	*ccagggcctaattttcatacc*
R03E9.2 prom2	ccgtcagttttgcaatggtc	gatatgatgttttcggttcc	*tacaggaagctttctagaag*
R03E9.2 prom3	gaaacgtgacgcaatctcatc	gtgtgtgggtgtgtgtgacac	*ggaaaacagccagctacacc*
R03E9.2 prom4	gccaaatattgcaagaatcgg	gtgacagtaaatacaaggag	*cgtgcgcgtaagatatgacg*
F49B2.6 prom1	gagactctcttacaccacgtg	cgctccattgagaaattgtag	*ctctatatgcaaatgcgctc*
F49B2.6 prom2	cttcttcttcttgtctgtgtg	gtgctttaccccaaaaaatg	*ggactctaagtagcaagggg*
nta-1 prom1	cccaactcctgactctaaaag	caattgtttgtgatttcccc	*cgttgacttttccatttccc*
nta-1 prom2	gcccttgtcacaggtaaattg	cgcaagttagtcaagatatg	*cccggtgatggttacctttg*
T14B4.9 prom1	cgatctttctccatgattgc	attagcatggtttccctcag	ctttgattatgacggacatgc

T14B4.9 prom2	cggagggaagtaaaatctta	cgatttactggtaactacca	gtacagtcatatttacagatgg
T14B4.9 prom3	ggcacctagaataacaatgag	ggagacgcaaagagacagct c	ctccaaatttgagctcagggg
tbc-12 prom1	cctgcagctcgttttttttg	gtcgttgtctactccattttg	*gagcaaaaagagagaaactc*
tbc-12 prom2	cagatataattccattgggc	cacgtttttctgttcggccac	*gacgagcaaatattaccttcc*
tbc-12 prom3	gttaacaaggccccgttgtcg	gctatttttcctgaagctcac	*ggaaattgaccacacaaatgg*
tbc-12 prom4	caaaccagttgcgtgaatgcc	gtggccatttaatcgcaattc	*ctaggggcttttaagtacttg*
pgp-4 prom1	gaggacataattttagccgc	gtgagcaacaaccatttttg	ctctgcttaacaatggttctg
pgp-4 prom2	ctgacctaaaaagacaacgtc	gcacacactacaacttacccg	ctggcaattttcattgggacc
haf-7 prom1	gctatatttttcatcgtggc	gttcacacacattattgctc	caggaaatgcaacaatatgc
haf-7 prom2	gggaaagccctgaaatctga	ggatttttattccaaccctc	gtgcacctagcaatttcatgc

**FOSMID RECOMBINEERING**		
**Fosmid clone (gene)**	**Primer F**	**Primer R**
WRM0641dF 02 (*gly-18*)	cacgaggtcttgccatgccacaactgacacataa aaattggattttatgagctgtctcatcctactttcac	aacgttgacccaaaaaatgaacccgaaatgaa aaaatgtactgaaaaaaactacttgctggaagtgt acttgg
WRM065dE0 1 (*gpx-8*)	acgataagggaaaactatatcaagttgaactttatt tatttcagagataagctgtctcatcctactttcac	atctaaaacttatgtaaagtctaaaacttgtatatat gttgtactaccacctacttgctggaagtgtacttgg
