# Supplementary material for: LET-381/FoxF and its target UNC-30/Pitx2 specify and maintain the molecular identity of C. elegans mesodermal glia that regulate motor behavior
Source: EMBO J. 2024 Feb 15;43(6):4. doi: 10.1038/s44318-024-00049-w (PMC10943081; doi:10.1038/s44318-024-00049-w)
Supplement: Supplementary file 6 — Movie EV2 [file 44318_2024_49_MOESM6_ESM.zip › Movie EV2/README.rtf]

Movie EV2. Locomotion of freely moving GLR glia-ablated animals. Movie was recorded at 2 frames per second. Video shows 36 frames per second.
