# Supplementary figures and images for: LET-381/FoxF and its target UNC-30/Pitx2 specify and maintain the molecular identity of C. elegans mesodermal glia that regulate motor behavior (part 1 of 3)
Source: EMBO J. 2024 Feb 15;43(6):4. doi: 10.1038/s44318-024-00049-w (PMC10943081; doi:10.1038/s44318-024-00049-w)

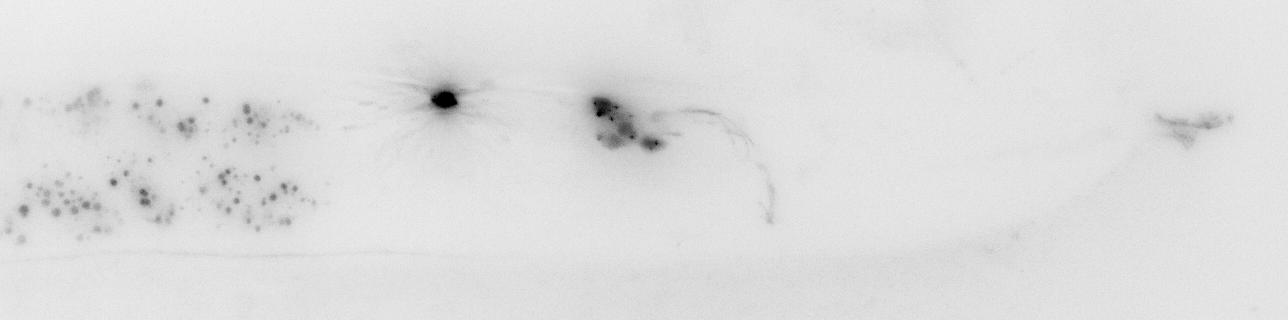

Supplement: Supplementary file 7 — Source Data Fig. 2 [file 44318_2024_49_MOESM7_ESM.zip › Figure 2/2E/2E RFP.tif]

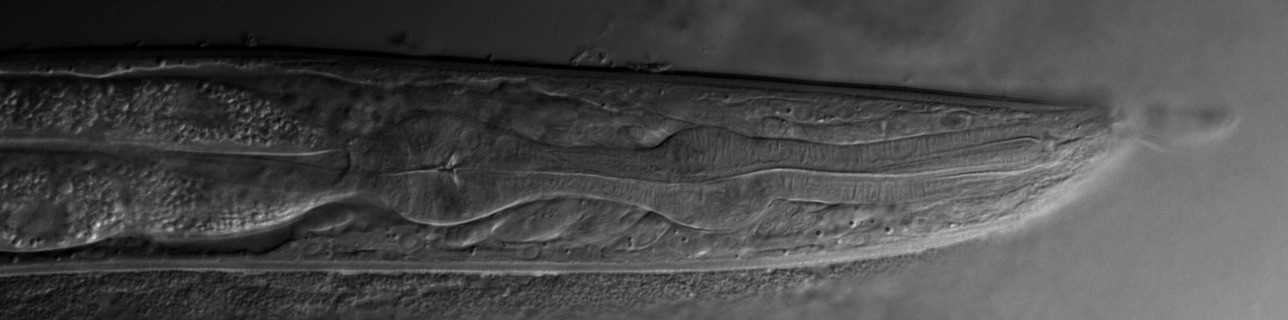

Supplement: Supplementary file 7 — Source Data Fig. 2 [file 44318_2024_49_MOESM7_ESM.zip › Figure 2/2E/2E DIC.tif]

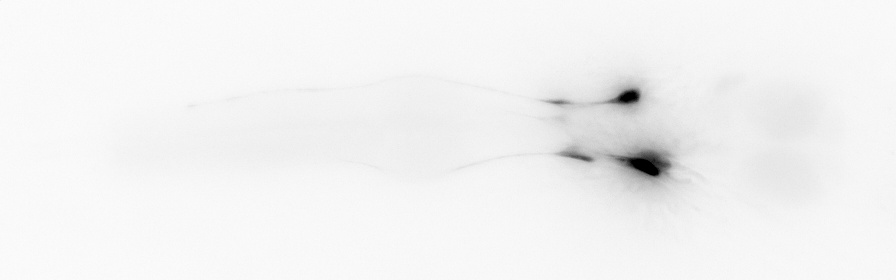

Supplement: Supplementary file 7 — Source Data Fig. 2 [file 44318_2024_49_MOESM7_ESM.zip › Figure 2/2C/2C left/2C left GFP.tif]

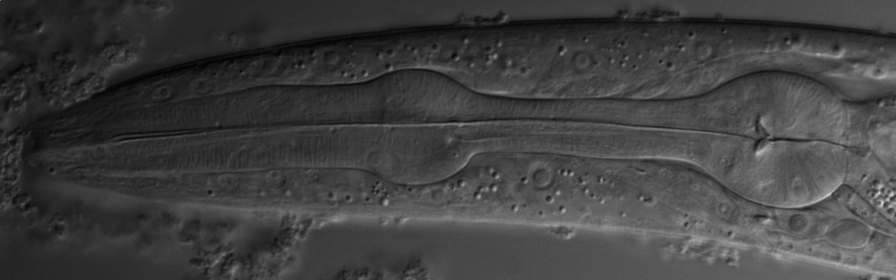

Supplement: Supplementary file 7 — Source Data Fig. 2 [file 44318_2024_49_MOESM7_ESM.zip › Figure 2/2C/2C left/2C left DIC.tif]

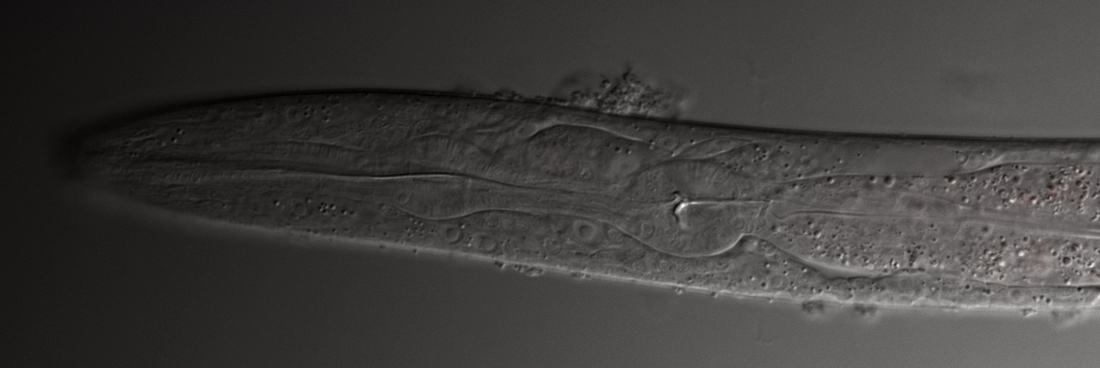

Supplement: Supplementary file 7 — Source Data Fig. 2 [file 44318_2024_49_MOESM7_ESM.zip › Figure 2/2C/2C right/2C right DIC.tif]

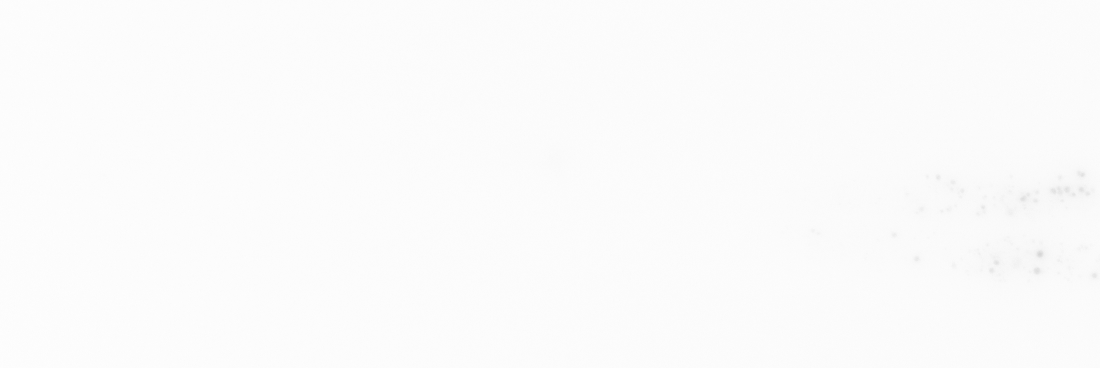

Supplement: Supplementary file 7 — Source Data Fig. 2 [file 44318_2024_49_MOESM7_ESM.zip › Figure 2/2C/2C right/2C right GFP.tif]

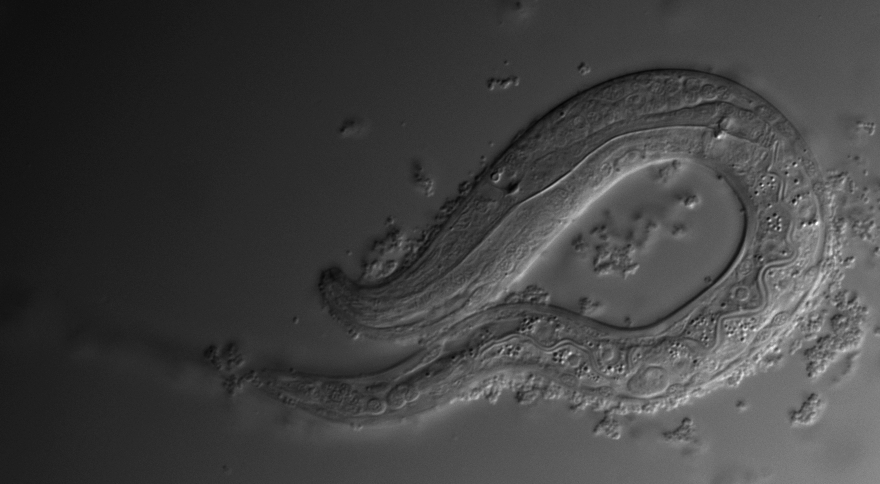

Supplement: Supplementary file 7 — Source Data Fig. 2 [file 44318_2024_49_MOESM7_ESM.zip › Figure 2/2D/2D right/2D right DIC.tif]

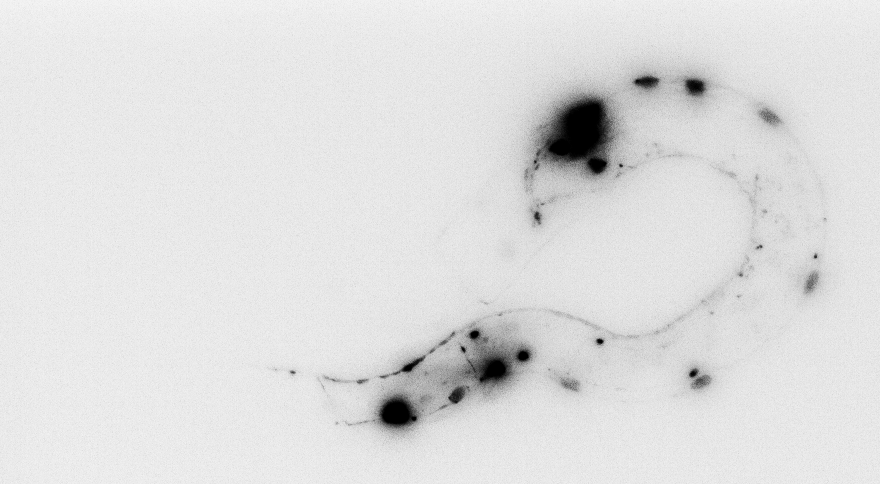

Supplement: Supplementary file 7 — Source Data Fig. 2 [file 44318_2024_49_MOESM7_ESM.zip › Figure 2/2D/2D right/2D right RFP.tif]

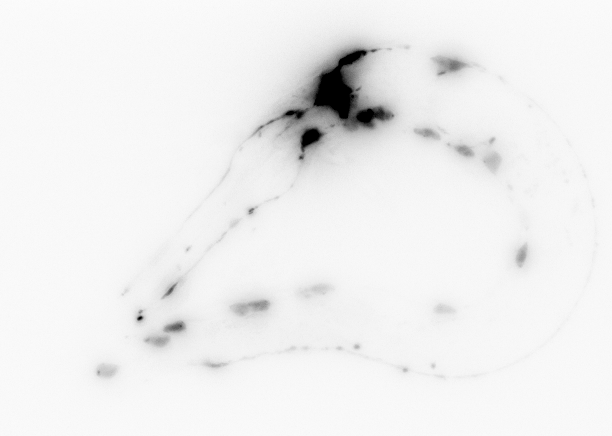

Supplement: Supplementary file 7 — Source Data Fig. 2 [file 44318_2024_49_MOESM7_ESM.zip › Figure 2/2D/2D left/2D left RFP.tif]

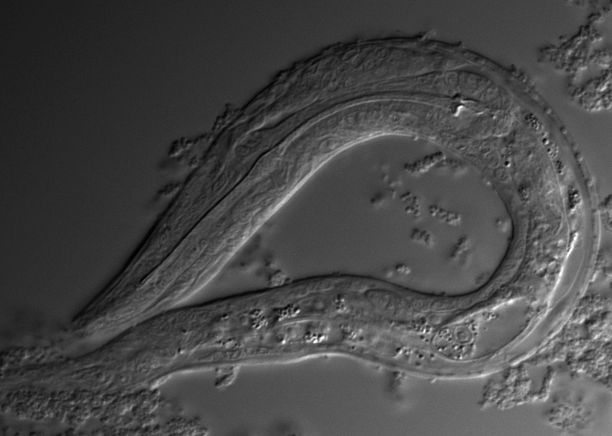

Supplement: Supplementary file 7 — Source Data Fig. 2 [file 44318_2024_49_MOESM7_ESM.zip › Figure 2/2D/2D left/2D left DIC.tif]

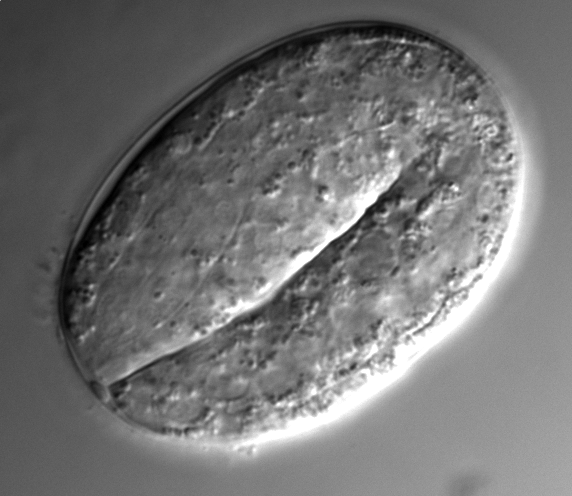

Supplement: Supplementary file 7 — Source Data Fig. 2 [file 44318_2024_49_MOESM7_ESM.zip › Figure 2/2B/2fold/DIC.tif]

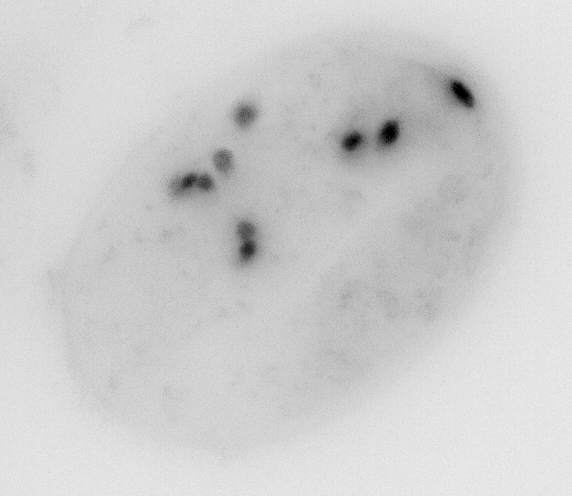

Supplement: Supplementary file 7 — Source Data Fig. 2 [file 44318_2024_49_MOESM7_ESM.zip › Figure 2/2B/2fold/2B 2fold GFP.tif]

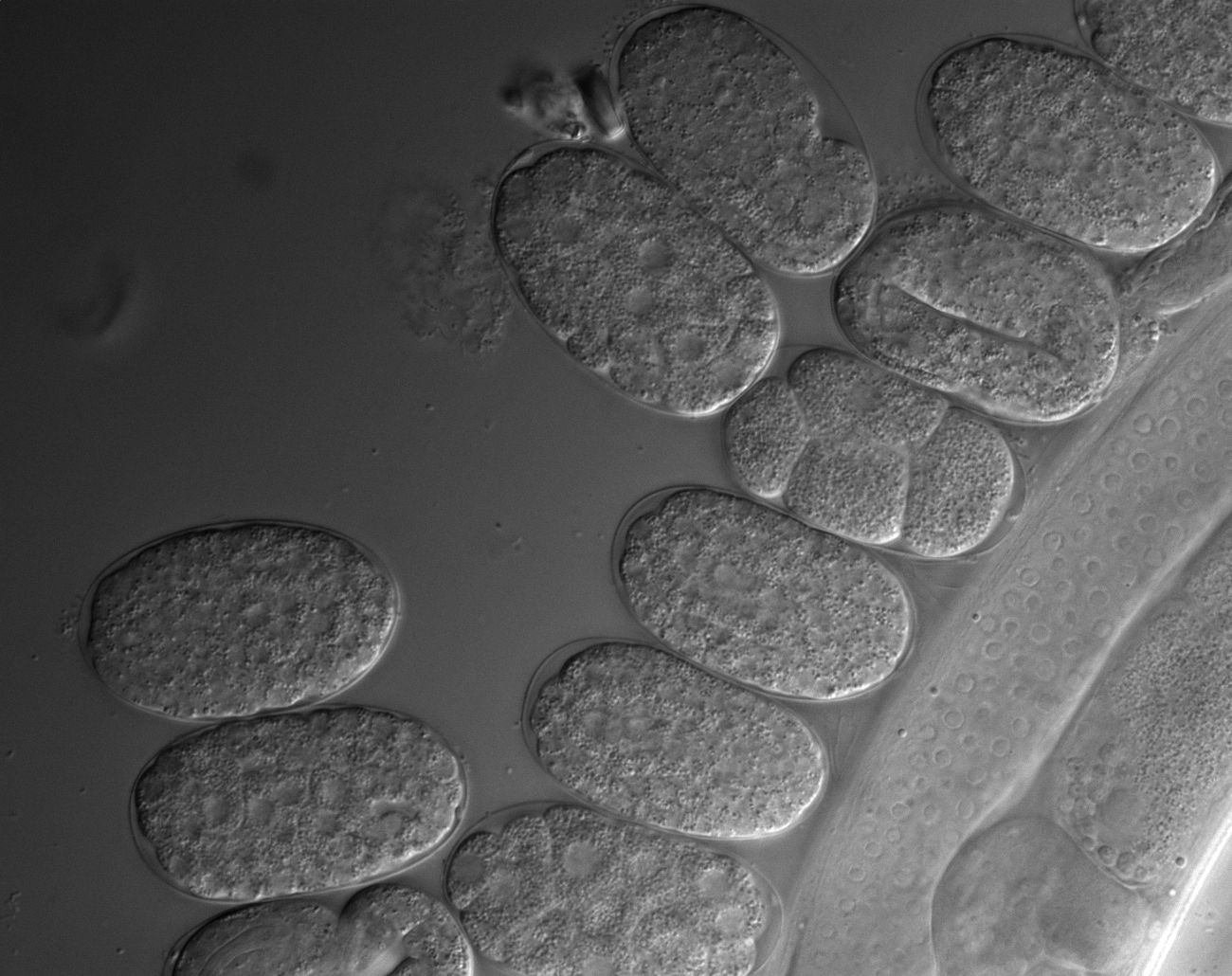

Supplement: Supplementary file 7 — Source Data Fig. 2 [file 44318_2024_49_MOESM7_ESM.zip › Figure 2/2B/prebean/DIC.tif]

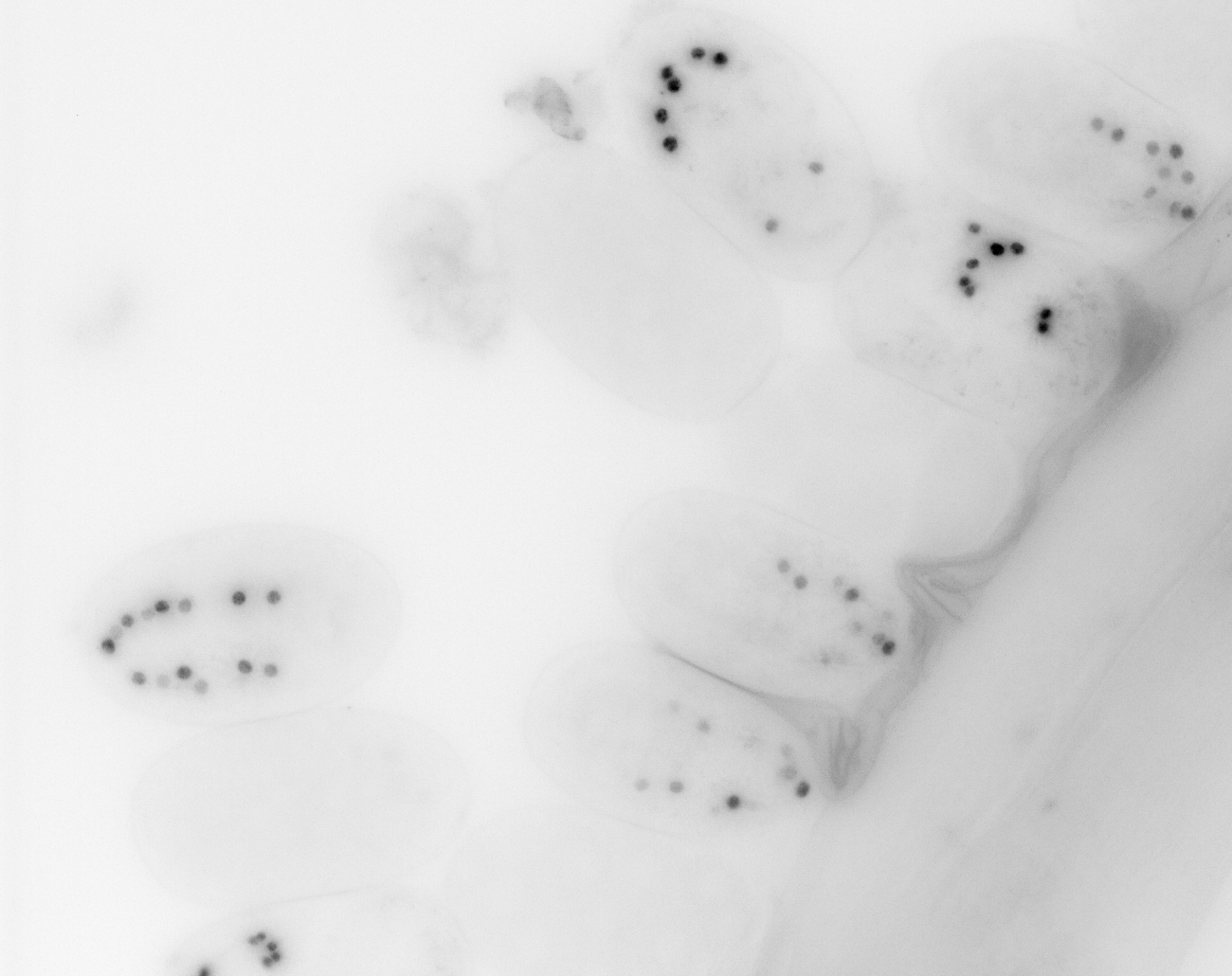

Supplement: Supplementary file 7 — Source Data Fig. 2 [file 44318_2024_49_MOESM7_ESM.zip › Figure 2/2B/prebean/2B prebean GFP.tif]

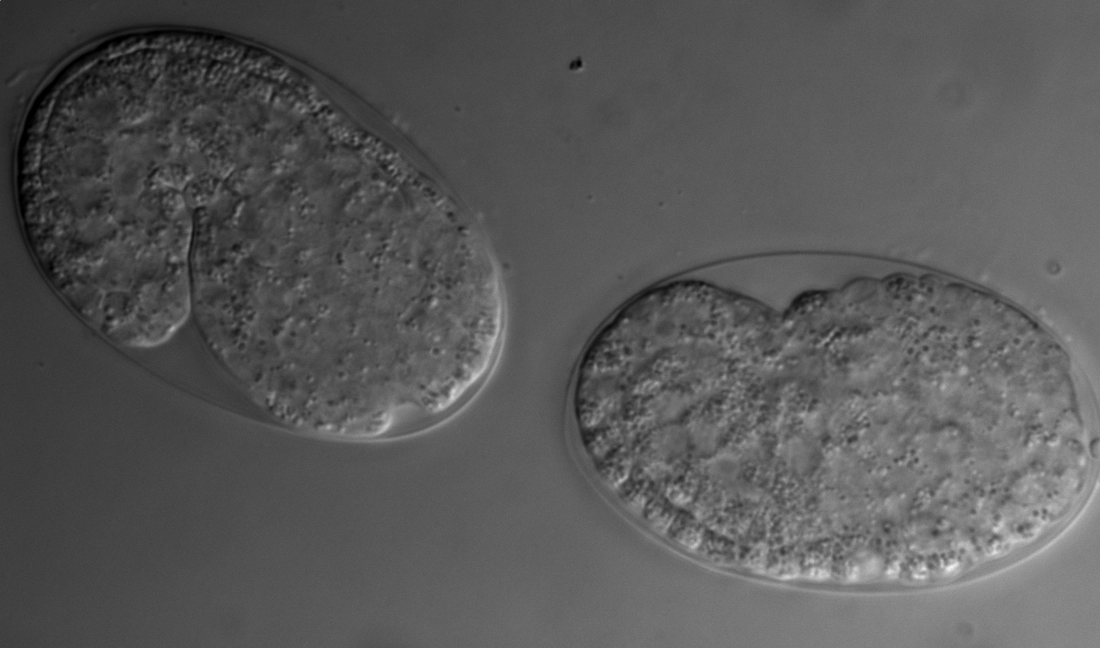

Supplement: Supplementary file 7 — Source Data Fig. 2 [file 44318_2024_49_MOESM7_ESM.zip › Figure 2/2B/bean/DIC.tif]

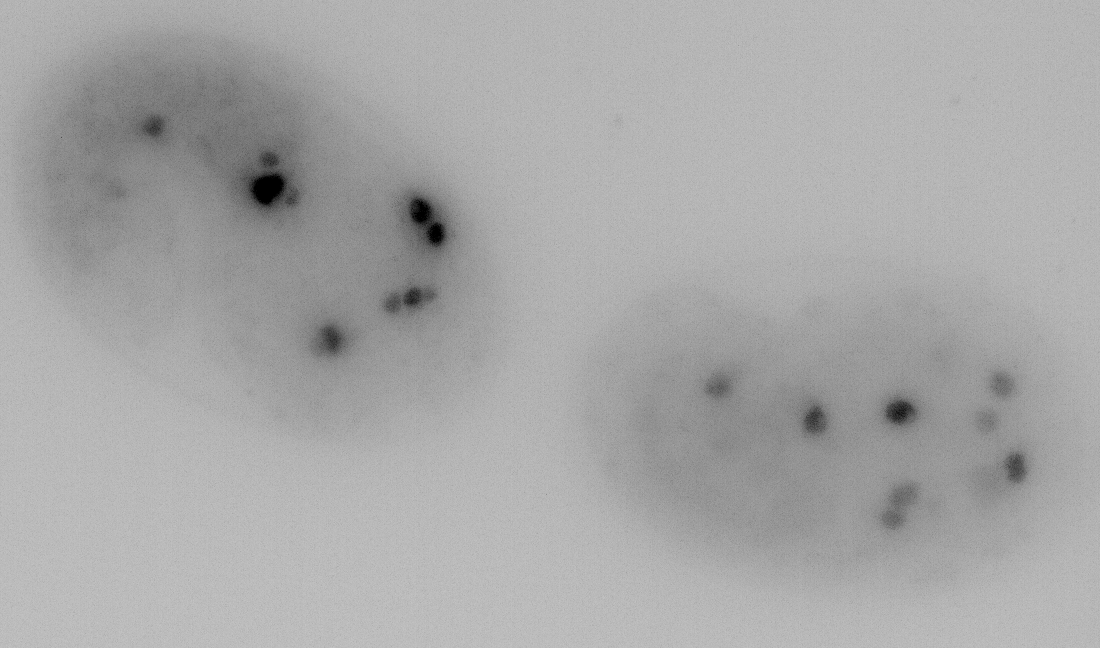

Supplement: Supplementary file 7 — Source Data Fig. 2 [file 44318_2024_49_MOESM7_ESM.zip › Figure 2/2B/bean/2B bean GFP.tif]

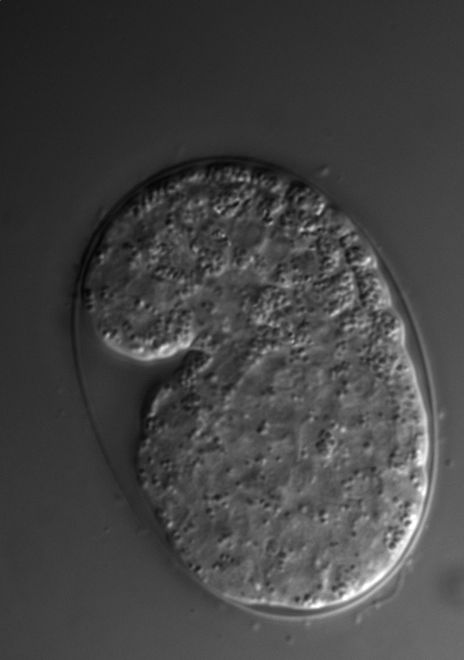

Supplement: Supplementary file 7 — Source Data Fig. 2 [file 44318_2024_49_MOESM7_ESM.zip › Figure 2/2B/comma/DIC.tif]

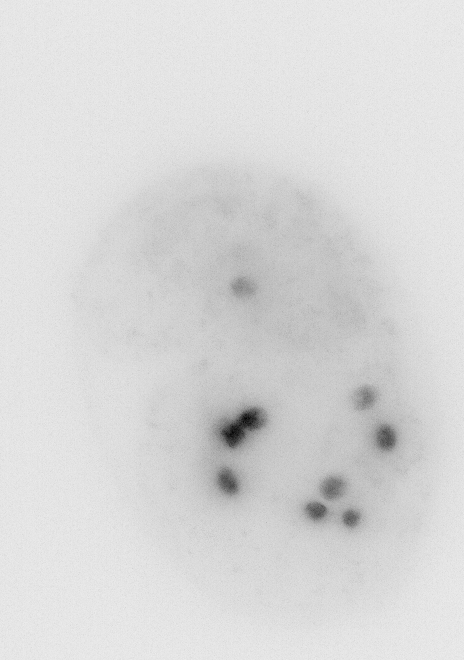

Supplement: Supplementary file 7 — Source Data Fig. 2 [file 44318_2024_49_MOESM7_ESM.zip › Figure 2/2B/comma/2B comma GFP.tif]

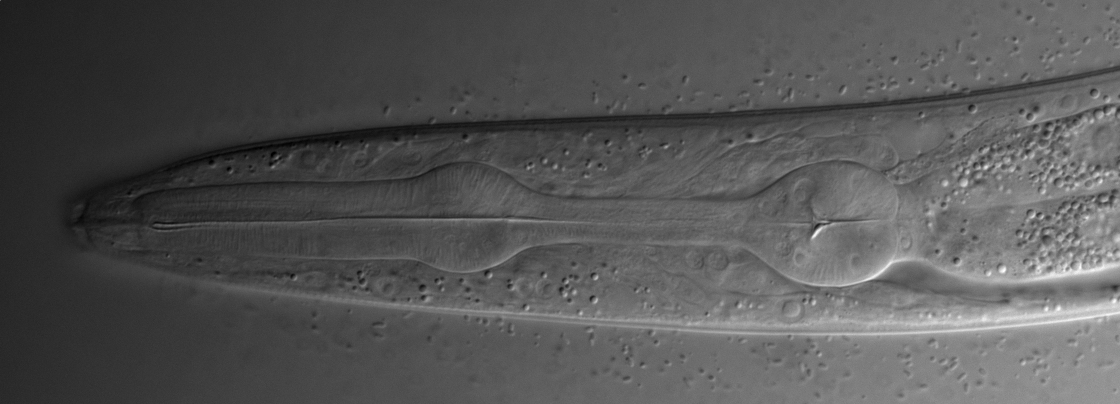

Supplement: Supplementary file 7 — Source Data Fig. 2 [file 44318_2024_49_MOESM7_ESM.zip › Figure 2/2B/Adult/DIC.tif]

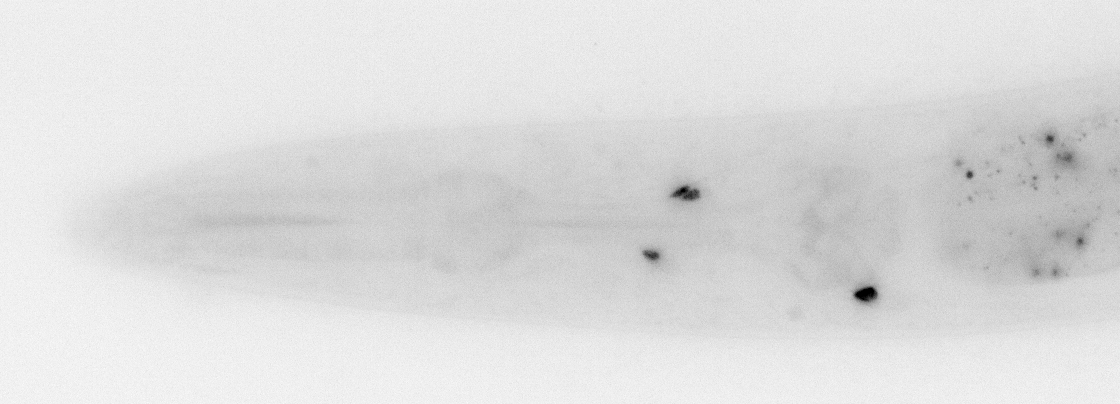

Supplement: Supplementary file 7 — Source Data Fig. 2 [file 44318_2024_49_MOESM7_ESM.zip › Figure 2/2B/Adult/2B adult GFP.tif]

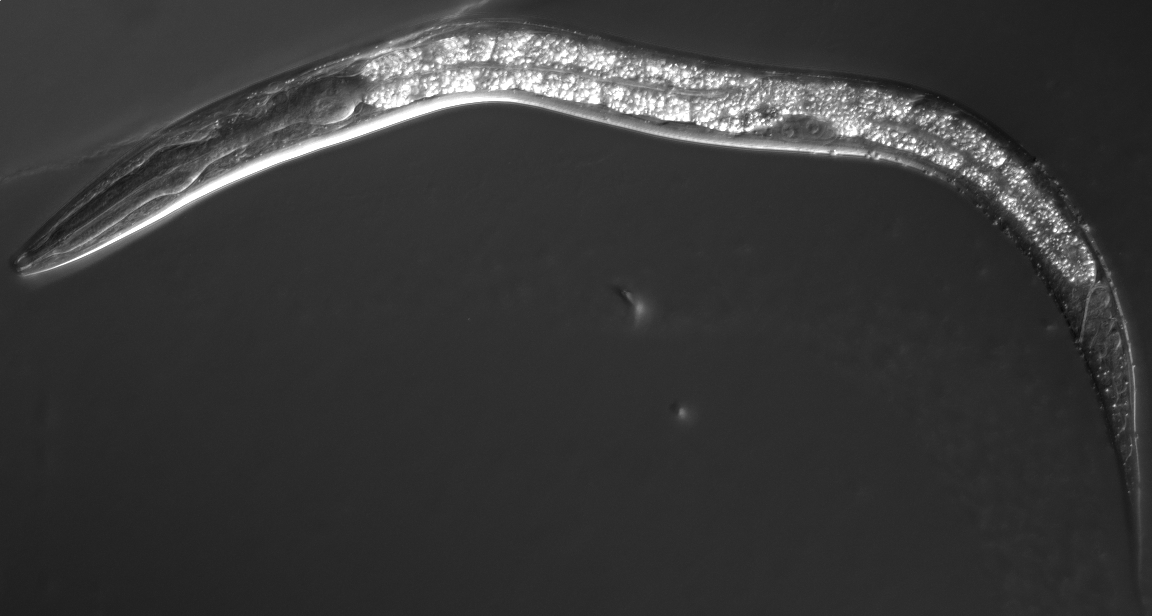

Supplement: Supplementary file 7 — Source Data Fig. 2 [file 44318_2024_49_MOESM7_ESM.zip › Figure 2/2B/L1/DIC.tif]

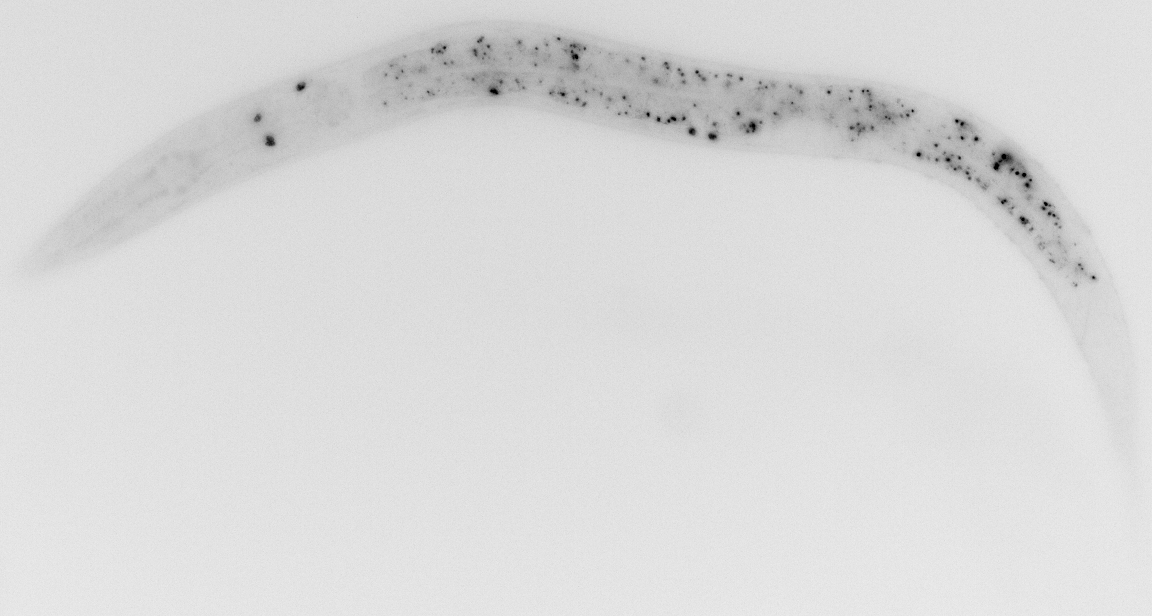

Supplement: Supplementary file 7 — Source Data Fig. 2 [file 44318_2024_49_MOESM7_ESM.zip › Figure 2/2B/L1/2B L1 GFP.tif]

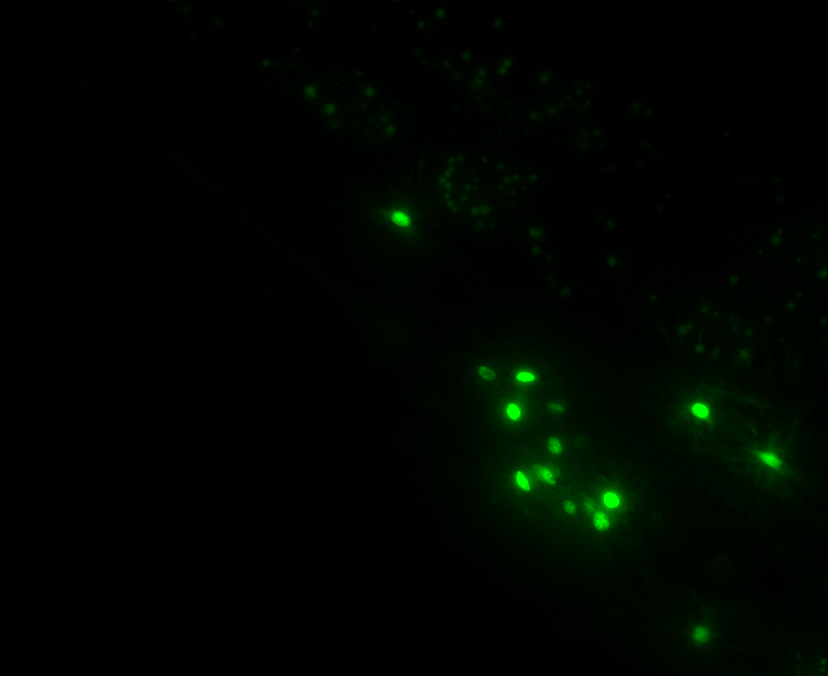

Supplement: Supplementary file 8 — Source Data Fig. 3 [file 44318_2024_49_MOESM8_ESM.zip › Figure 3/3E/right/3E right RFP (green).tif]

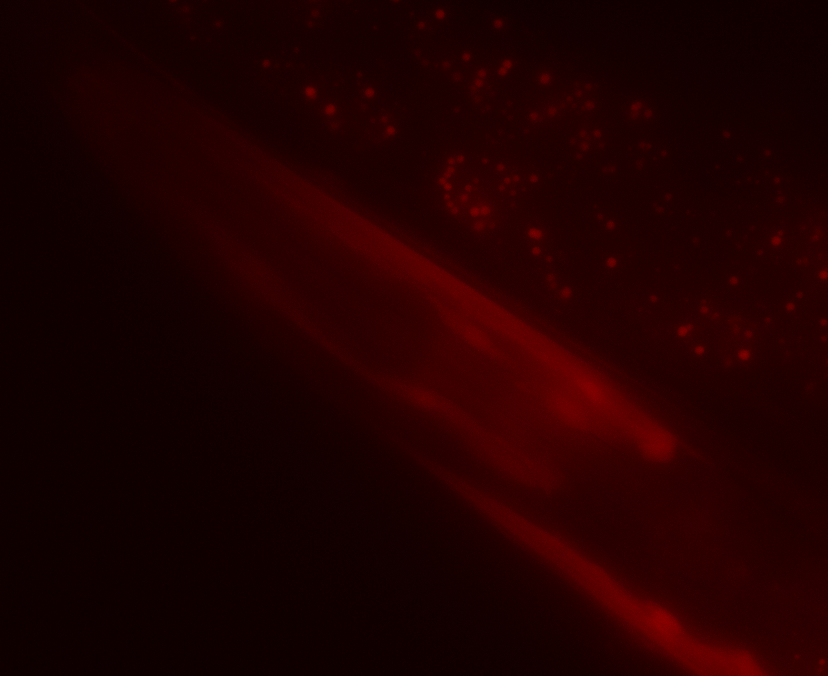

Supplement: Supplementary file 8 — Source Data Fig. 3 [file 44318_2024_49_MOESM8_ESM.zip › Figure 3/3E/right/3E right GFP (magenta).tif]

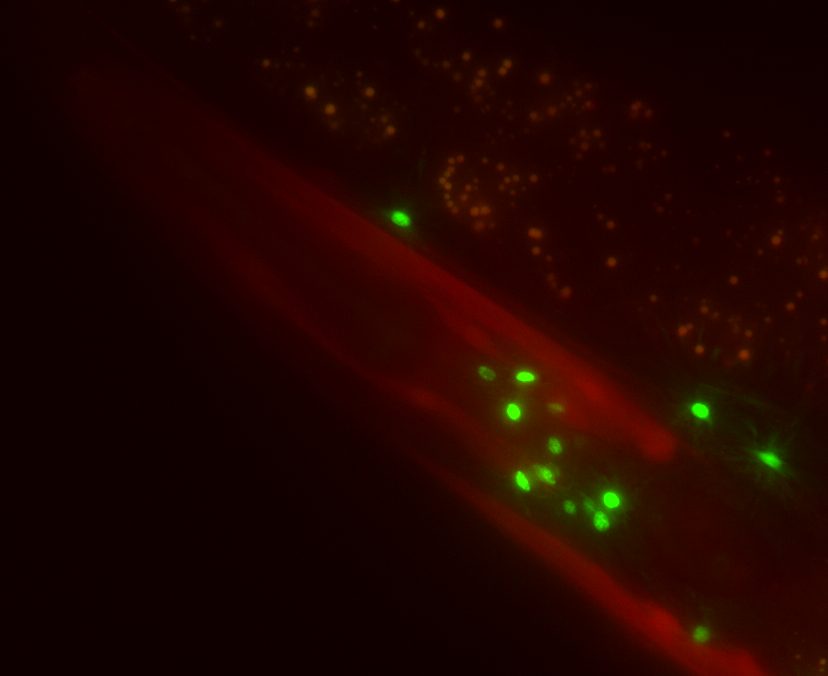

Supplement: Supplementary file 8 — Source Data Fig. 3 [file 44318_2024_49_MOESM8_ESM.zip › Figure 3/3E/right/3E right merge.tif]

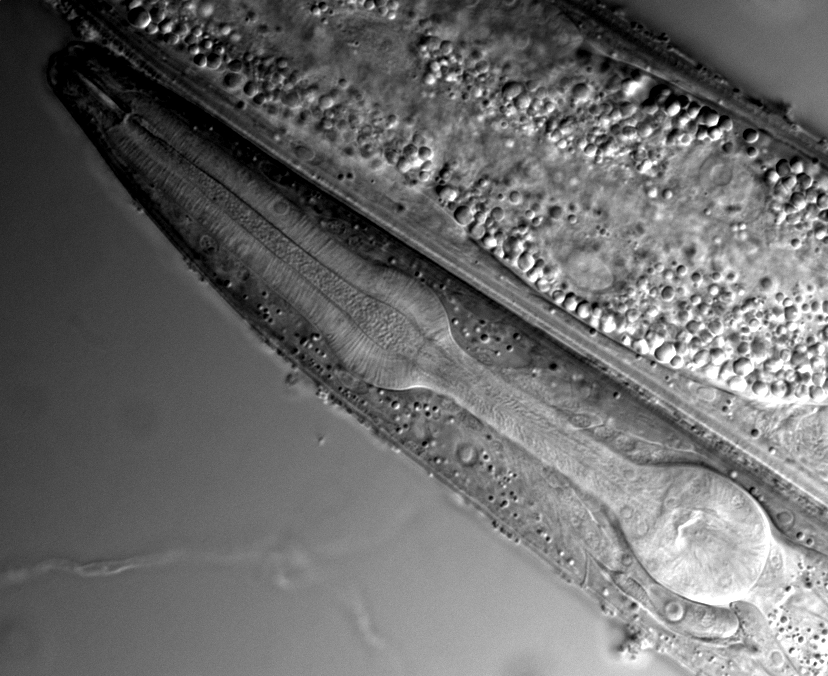

Supplement: Supplementary file 8 — Source Data Fig. 3 [file 44318_2024_49_MOESM8_ESM.zip › Figure 3/3E/right/3E right DIC.tif]

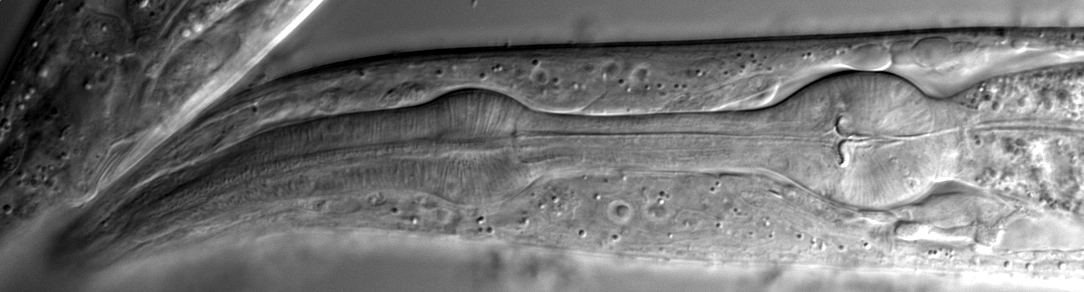

Supplement: Supplementary file 8 — Source Data Fig. 3 [file 44318_2024_49_MOESM8_ESM.zip › Figure 3/3E/middle/3E middle DIC.tif]

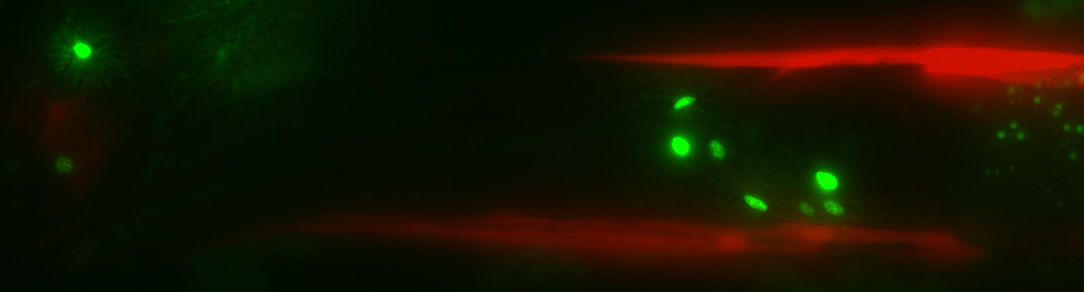

Supplement: Supplementary file 8 — Source Data Fig. 3 [file 44318_2024_49_MOESM8_ESM.zip › Figure 3/3E/middle/3E middle merge.tif]

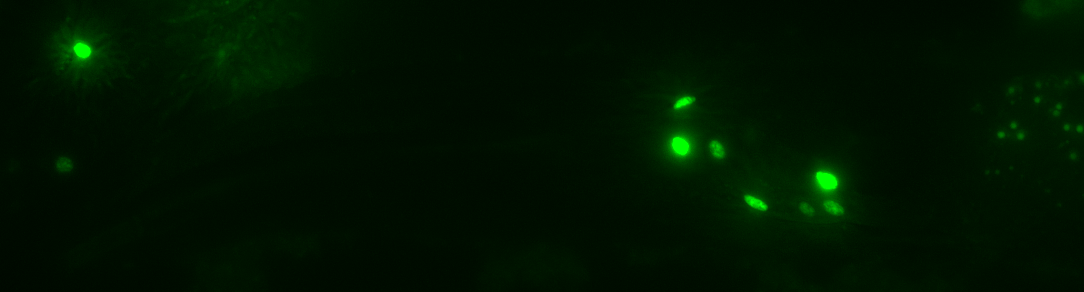

Supplement: Supplementary file 8 — Source Data Fig. 3 [file 44318_2024_49_MOESM8_ESM.zip › Figure 3/3E/middle/3E middle RFP (green).tif]

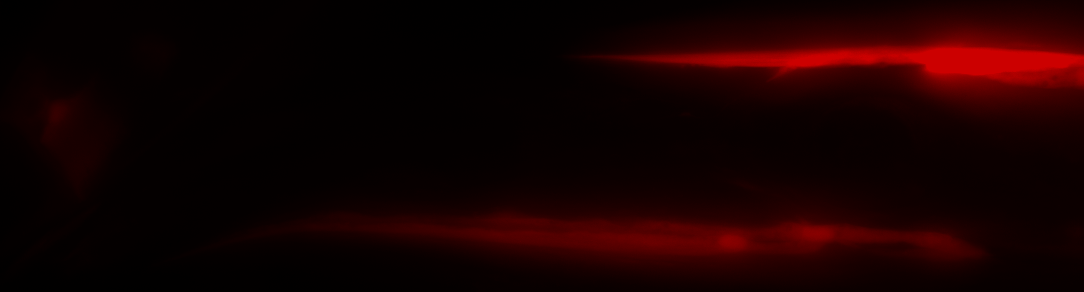

Supplement: Supplementary file 8 — Source Data Fig. 3 [file 44318_2024_49_MOESM8_ESM.zip › Figure 3/3E/middle/3E middle GFP (magenta).tif]

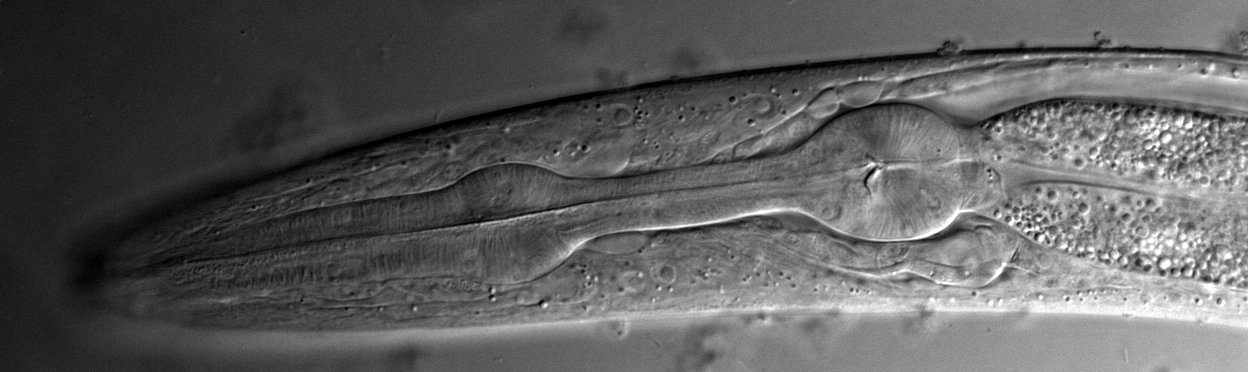

Supplement: Supplementary file 8 — Source Data Fig. 3 [file 44318_2024_49_MOESM8_ESM.zip › Figure 3/3E/left/3E left DIC.tif]

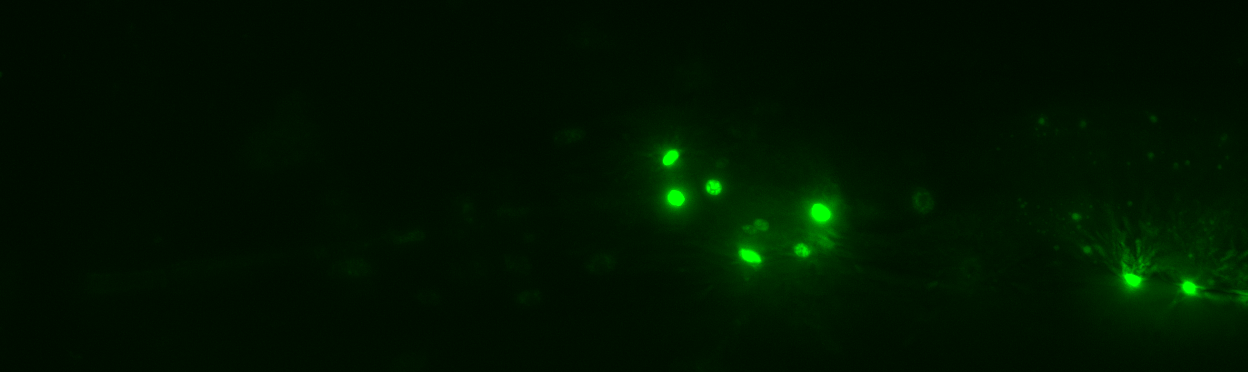

Supplement: Supplementary file 8 — Source Data Fig. 3 [file 44318_2024_49_MOESM8_ESM.zip › Figure 3/3E/left/3E left RFP (green).tif]

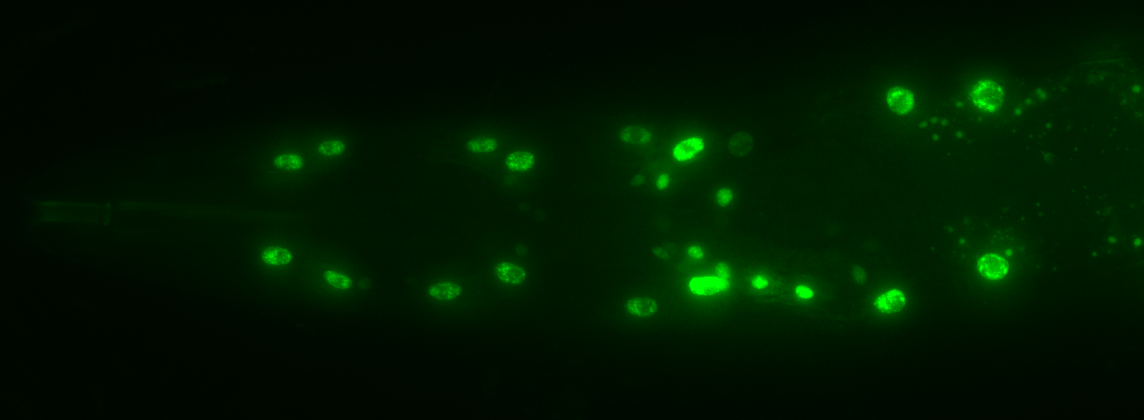

Supplement: Supplementary file 8 — Source Data Fig. 3 [file 44318_2024_49_MOESM8_ESM.zip › Figure 3/3B/right/3B right GFP.tif]

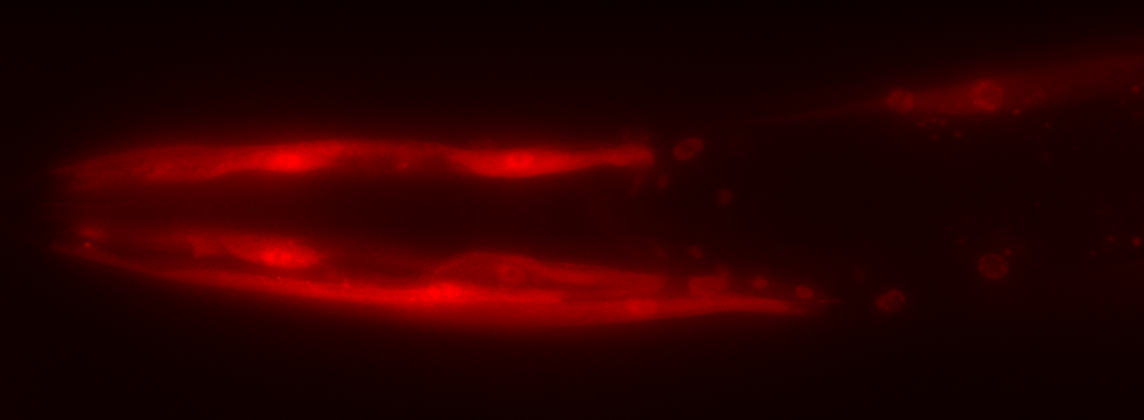

Supplement: Supplementary file 8 — Source Data Fig. 3 [file 44318_2024_49_MOESM8_ESM.zip › Figure 3/3B/right/3B right RFP (magenta).tif]

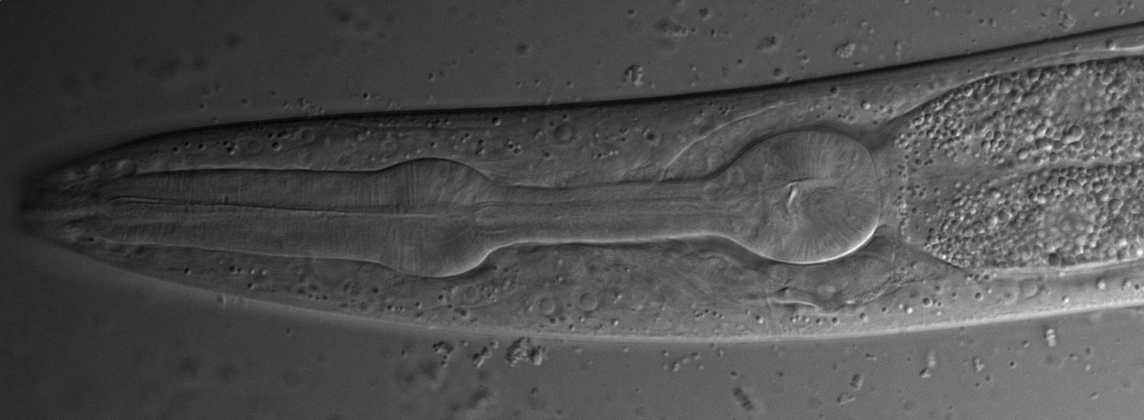

Supplement: Supplementary file 8 — Source Data Fig. 3 [file 44318_2024_49_MOESM8_ESM.zip › Figure 3/3B/right/3B right DIC.tif]

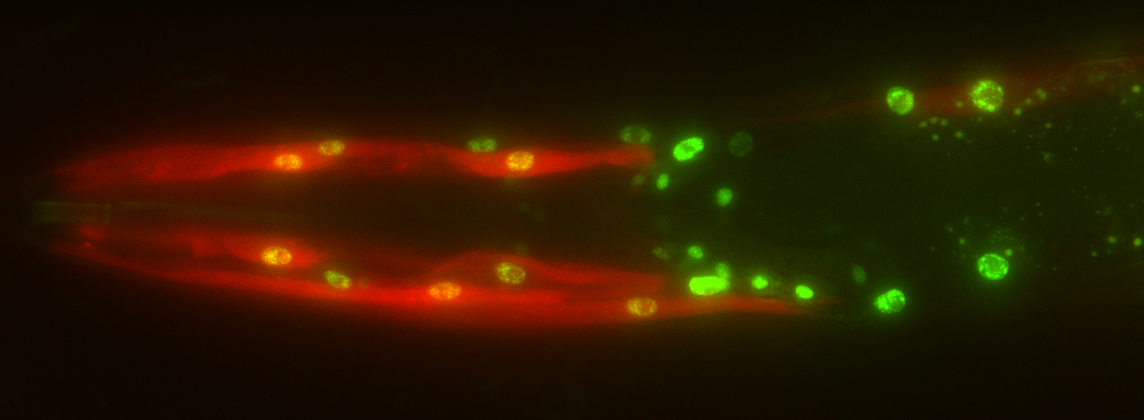

Supplement: Supplementary file 8 — Source Data Fig. 3 [file 44318_2024_49_MOESM8_ESM.zip › Figure 3/3B/right/3B right merge.tif]

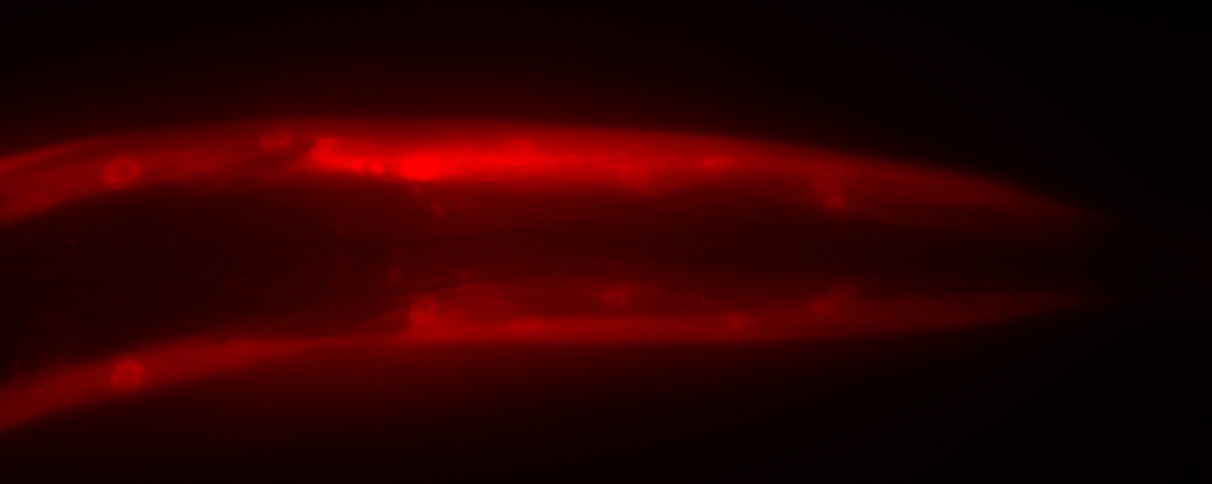

Supplement: Supplementary file 8 — Source Data Fig. 3 [file 44318_2024_49_MOESM8_ESM.zip › Figure 3/3B/middle/3B middle RFP (magenta).tif]

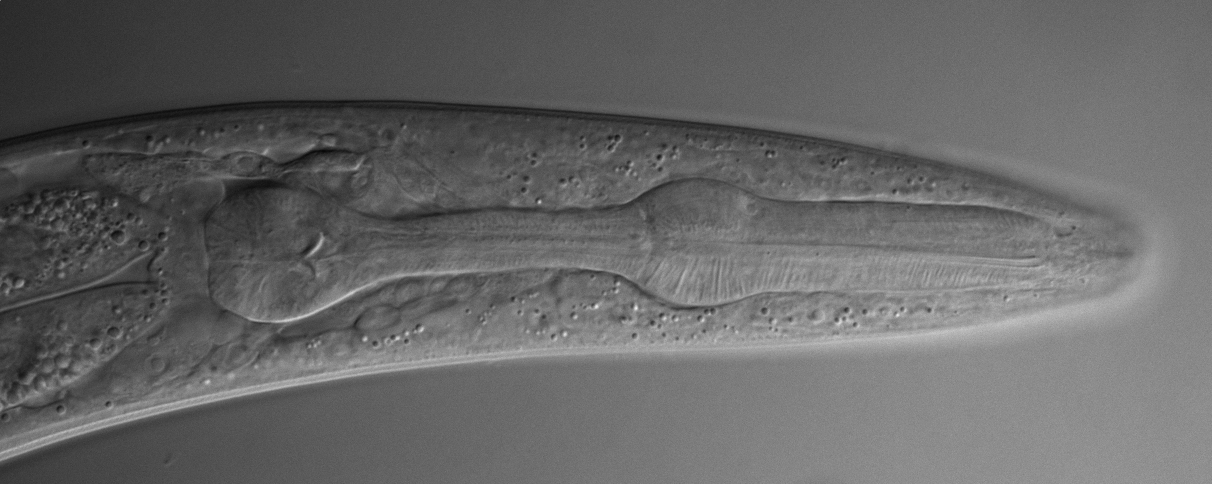

Supplement: Supplementary file 8 — Source Data Fig. 3 [file 44318_2024_49_MOESM8_ESM.zip › Figure 3/3B/middle/3B middle DIC.tif]

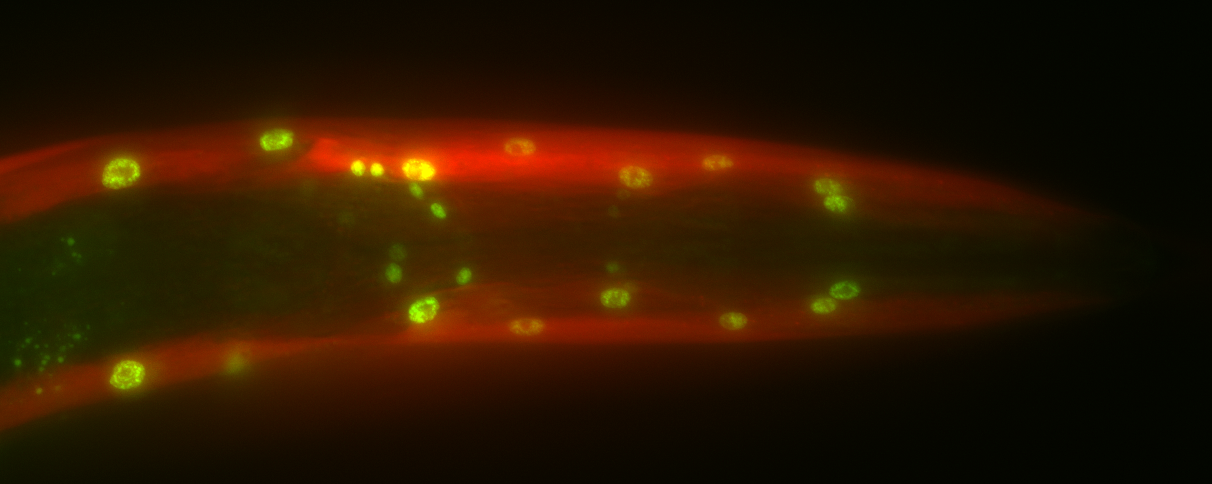

Supplement: Supplementary file 8 — Source Data Fig. 3 [file 44318_2024_49_MOESM8_ESM.zip › Figure 3/3B/middle/3B middle merge.tif]

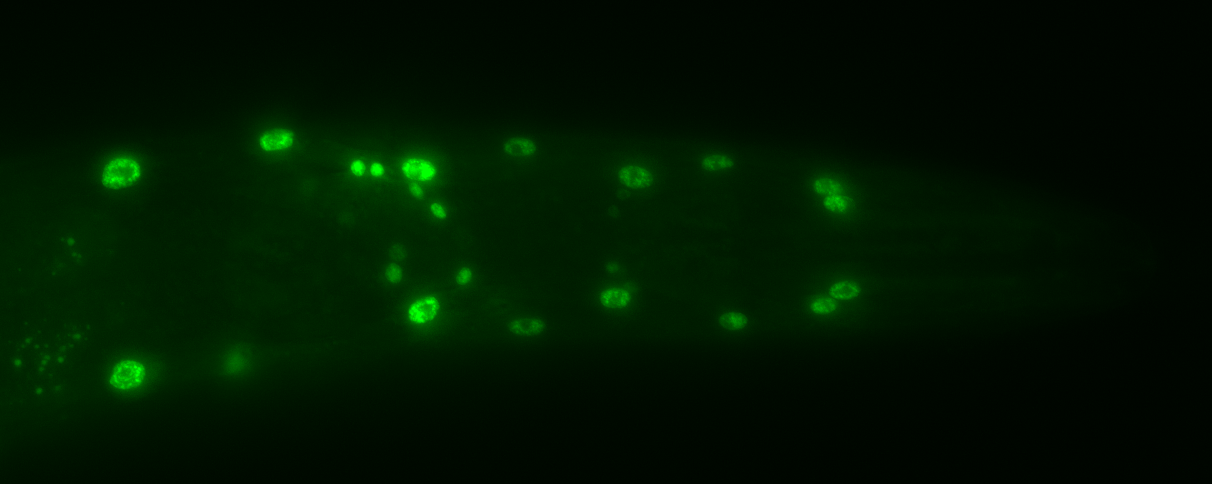

Supplement: Supplementary file 8 — Source Data Fig. 3 [file 44318_2024_49_MOESM8_ESM.zip › Figure 3/3B/middle/3B middle GFP.tif]

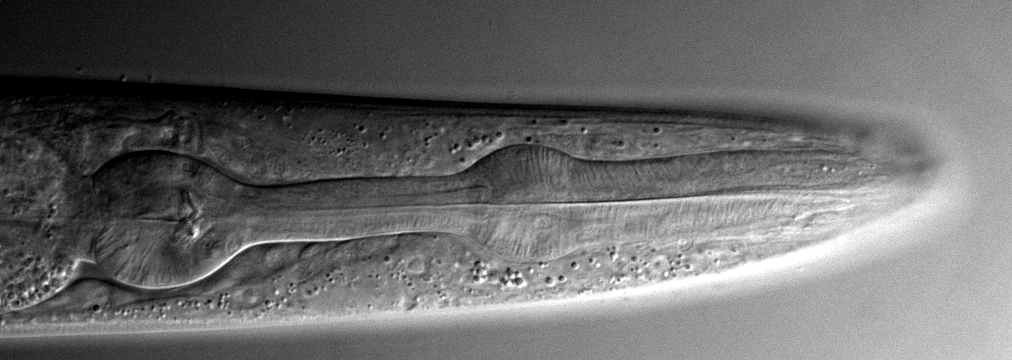

Supplement: Supplementary file 8 — Source Data Fig. 3 [file 44318_2024_49_MOESM8_ESM.zip › Figure 3/3B/left/3B left DIC wt.tif]

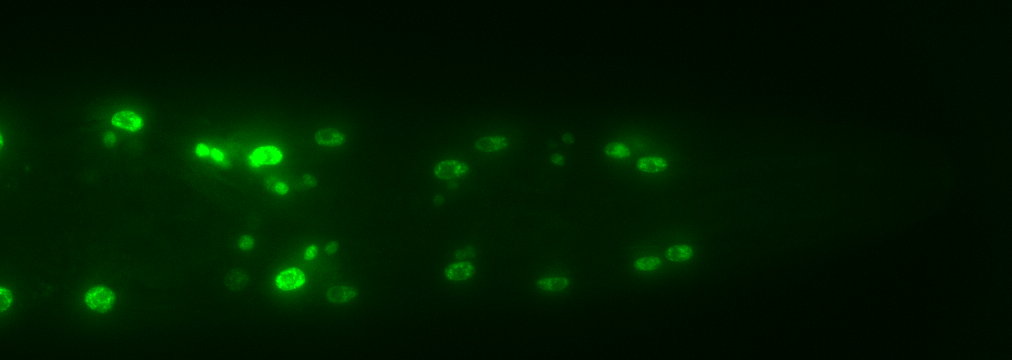

Supplement: Supplementary file 8 — Source Data Fig. 3 [file 44318_2024_49_MOESM8_ESM.zip › Figure 3/3B/left/3B left GFP.tif]

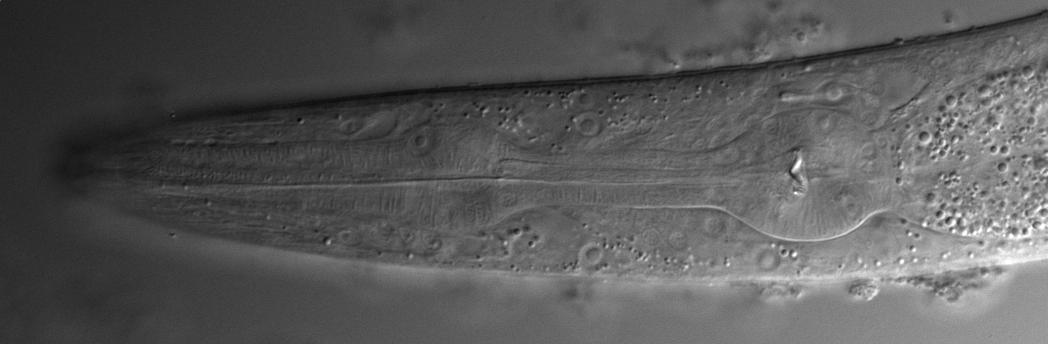

Supplement: Supplementary file 8 — Source Data Fig. 3 [file 44318_2024_49_MOESM8_ESM.zip › Figure 3/3C/right/3C right DIC.tif]

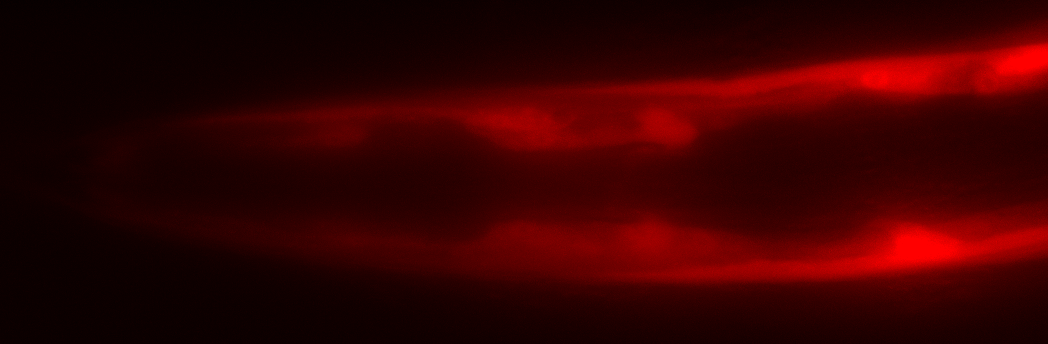

Supplement: Supplementary file 8 — Source Data Fig. 3 [file 44318_2024_49_MOESM8_ESM.zip › Figure 3/3C/right/3C right RFP (magenta).tif]

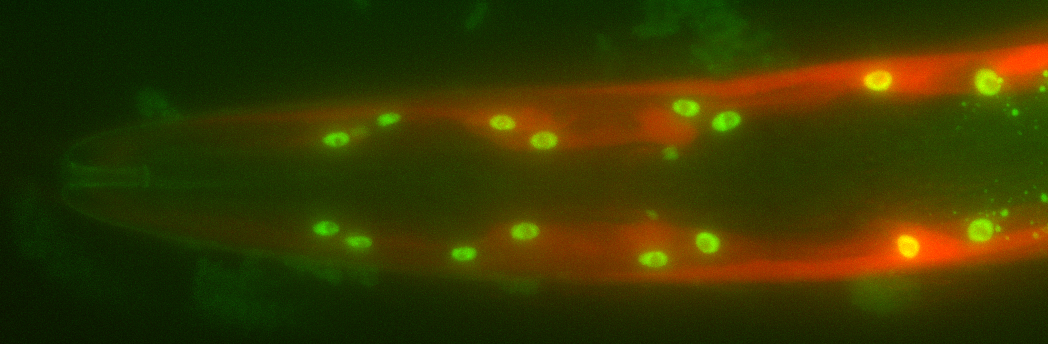

Supplement: Supplementary file 8 — Source Data Fig. 3 [file 44318_2024_49_MOESM8_ESM.zip › Figure 3/3C/right/3C right merge.tif]

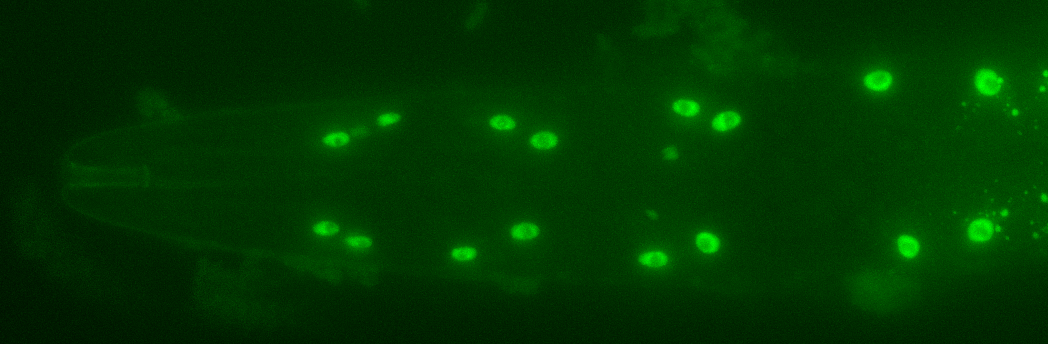

Supplement: Supplementary file 8 — Source Data Fig. 3 [file 44318_2024_49_MOESM8_ESM.zip › Figure 3/3C/right/3C right GFP.tif]

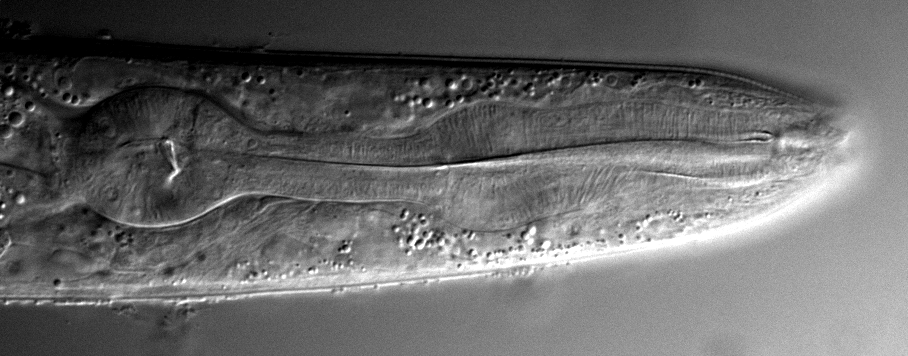

Supplement: Supplementary file 8 — Source Data Fig. 3 [file 44318_2024_49_MOESM8_ESM.zip › Figure 3/3C/middle/3C middle DIC.tif]

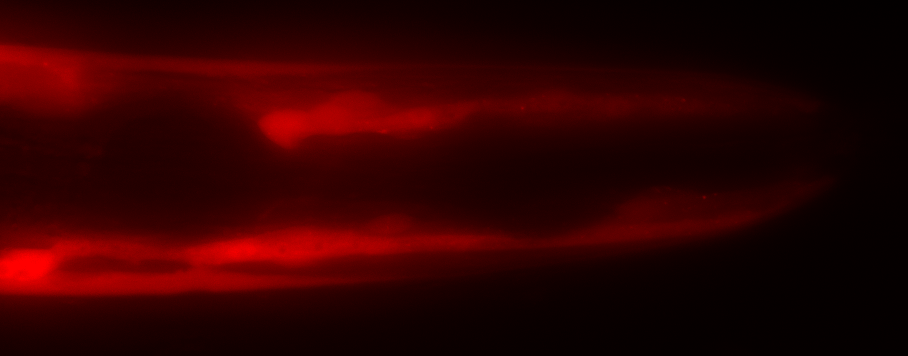

Supplement: Supplementary file 8 — Source Data Fig. 3 [file 44318_2024_49_MOESM8_ESM.zip › Figure 3/3C/middle/3C middle RFP (magenta).tif]

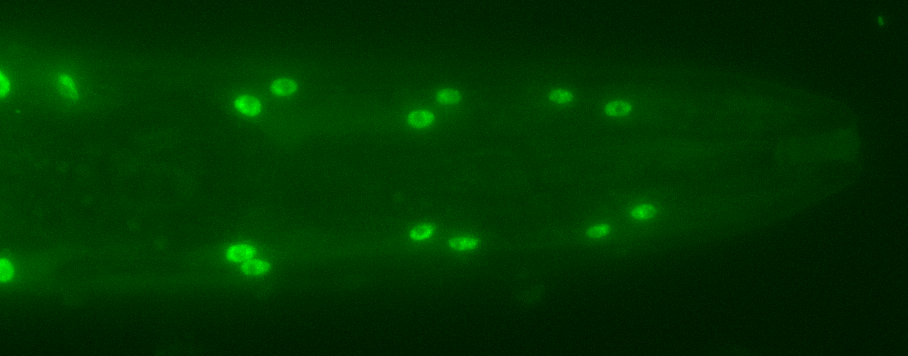

Supplement: Supplementary file 8 — Source Data Fig. 3 [file 44318_2024_49_MOESM8_ESM.zip › Figure 3/3C/middle/3C middle GFP.tif]

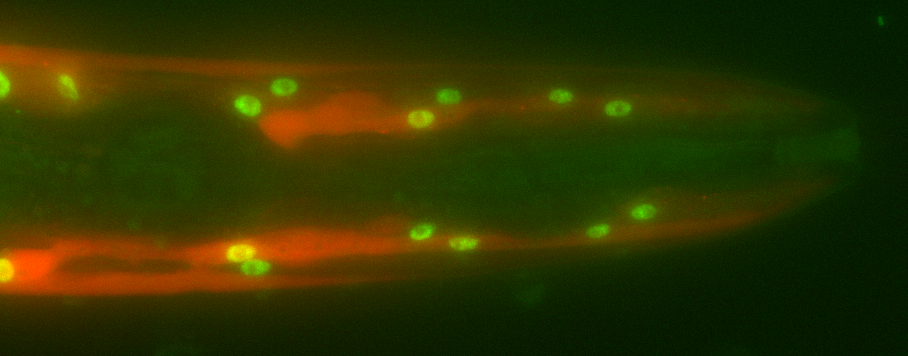

Supplement: Supplementary file 8 — Source Data Fig. 3 [file 44318_2024_49_MOESM8_ESM.zip › Figure 3/3C/middle/3C middle merge.tif]

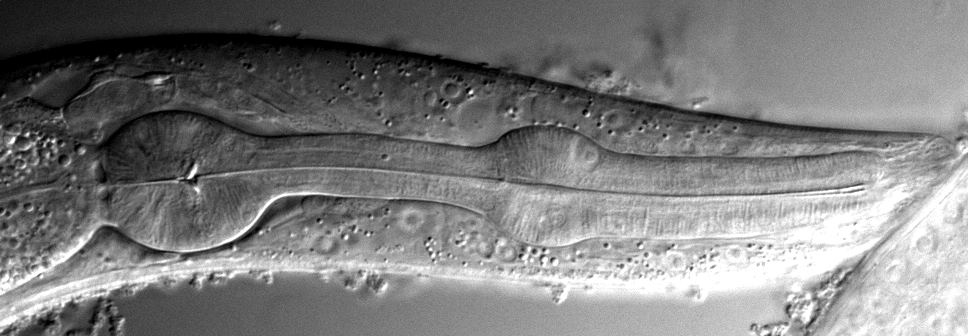

Supplement: Supplementary file 8 — Source Data Fig. 3 [file 44318_2024_49_MOESM8_ESM.zip › Figure 3/3C/left/3C left DIC.tif]

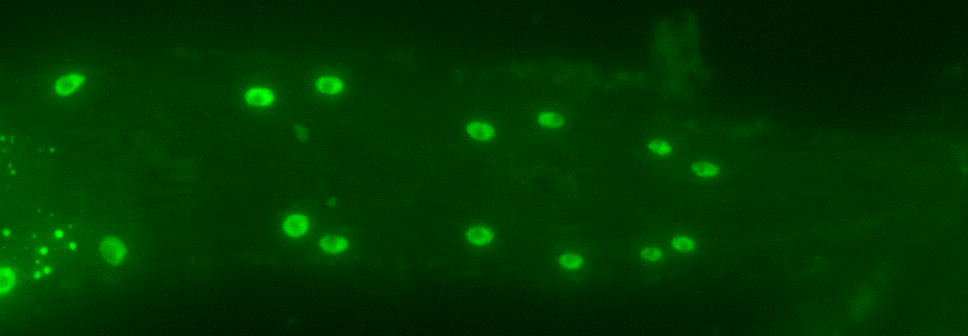

Supplement: Supplementary file 8 — Source Data Fig. 3 [file 44318_2024_49_MOESM8_ESM.zip › Figure 3/3C/left/3C left GFP.tif]

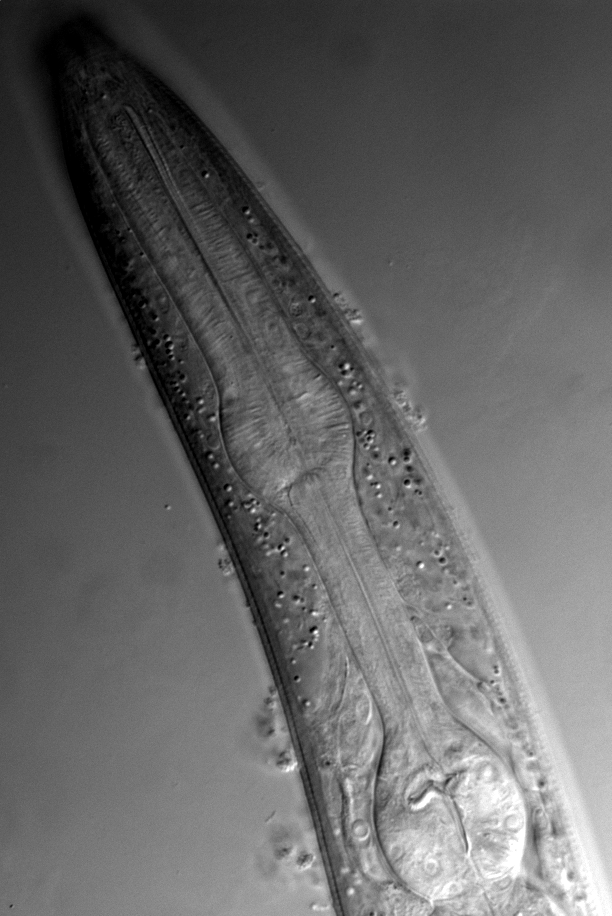

Supplement: Supplementary file 8 — Source Data Fig. 3 [file 44318_2024_49_MOESM8_ESM.zip › Figure 3/3D/right/3D right DIC.tif]

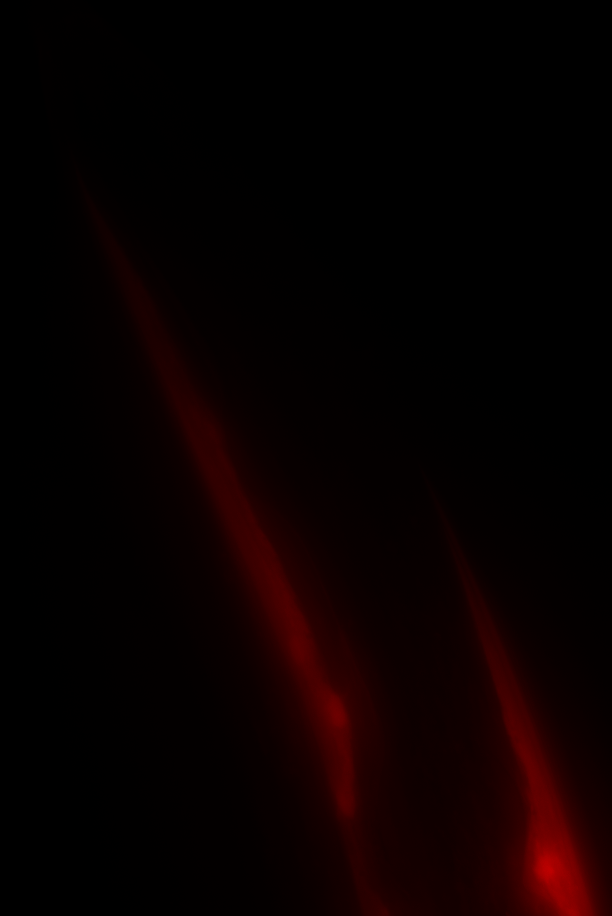

Supplement: Supplementary file 8 — Source Data Fig. 3 [file 44318_2024_49_MOESM8_ESM.zip › Figure 3/3D/right/3D right GFP (magenta).tif]

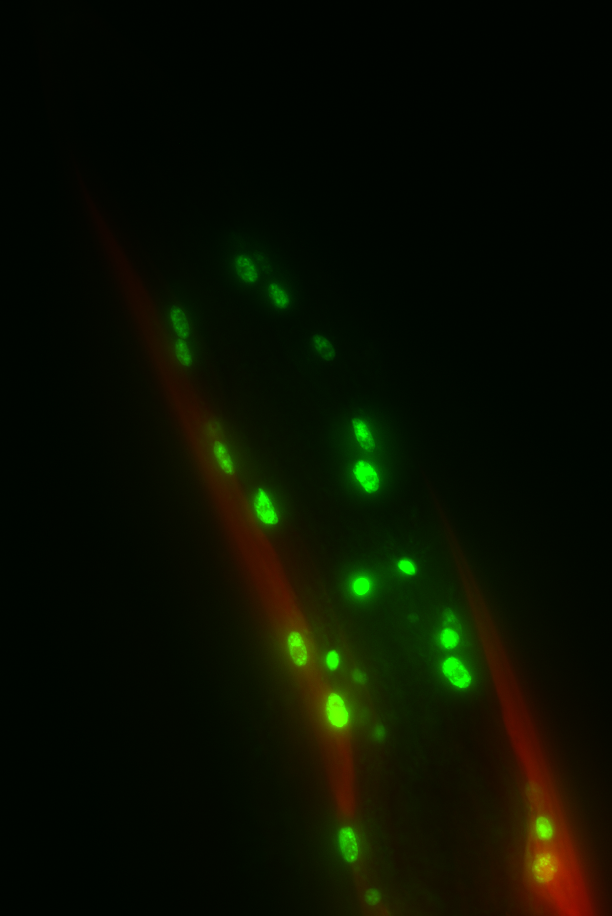

Supplement: Supplementary file 8 — Source Data Fig. 3 [file 44318_2024_49_MOESM8_ESM.zip › Figure 3/3D/right/3D right merge.tif]

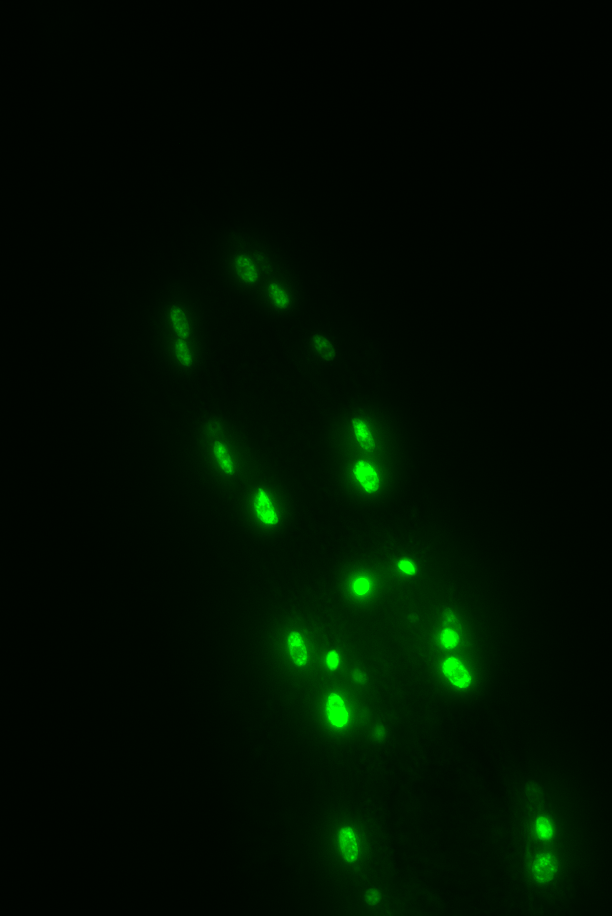

Supplement: Supplementary file 8 — Source Data Fig. 3 [file 44318_2024_49_MOESM8_ESM.zip › Figure 3/3D/right/3D right RFP (green).tif]

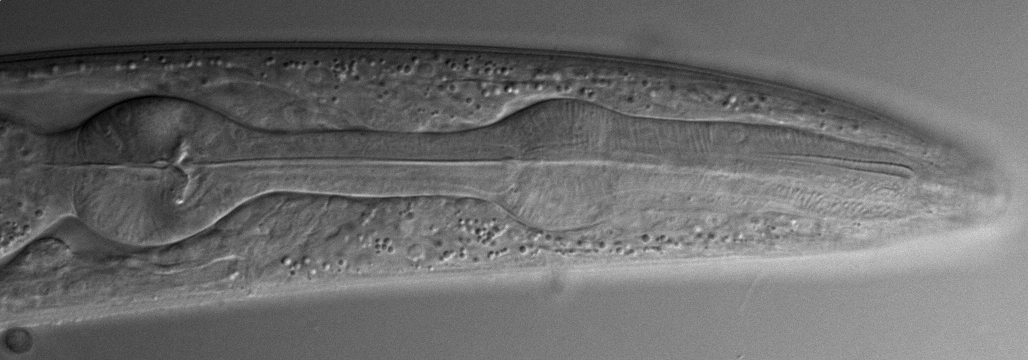

Supplement: Supplementary file 8 — Source Data Fig. 3 [file 44318_2024_49_MOESM8_ESM.zip › Figure 3/3D/middle/3D middle DIC.tif]

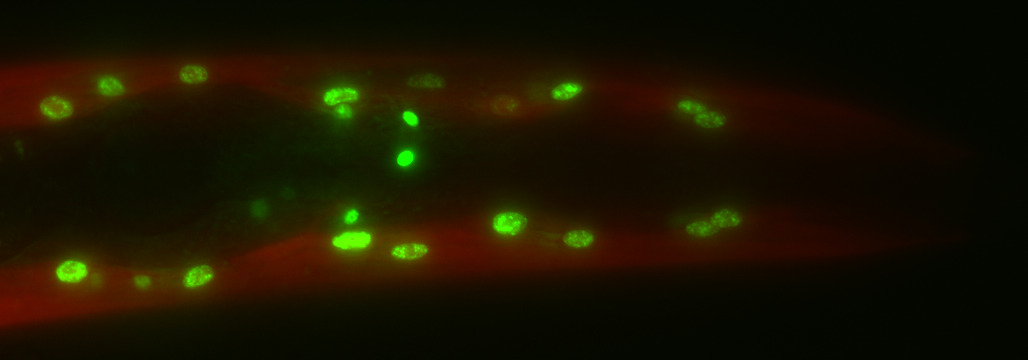

Supplement: Supplementary file 8 — Source Data Fig. 3 [file 44318_2024_49_MOESM8_ESM.zip › Figure 3/3D/middle/3D middle merge.tif]

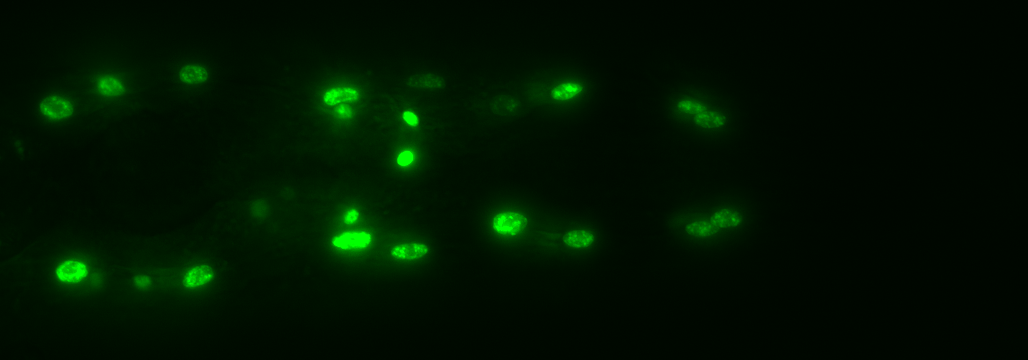

Supplement: Supplementary file 8 — Source Data Fig. 3 [file 44318_2024_49_MOESM8_ESM.zip › Figure 3/3D/middle/3D middle RFP (green).tif]

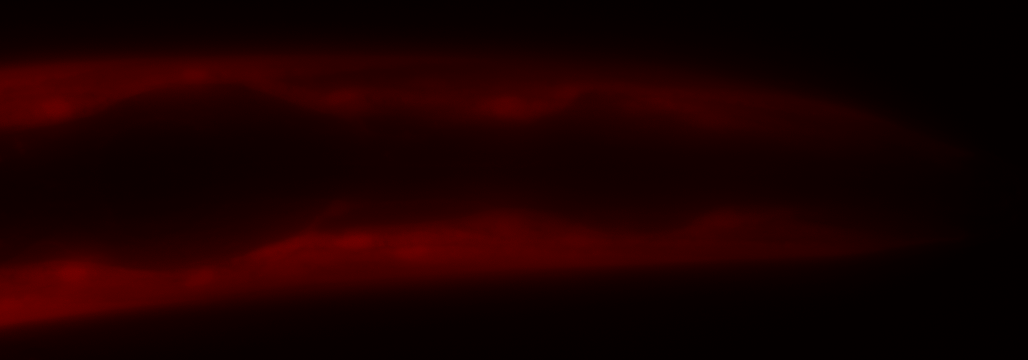

Supplement: Supplementary file 8 — Source Data Fig. 3 [file 44318_2024_49_MOESM8_ESM.zip › Figure 3/3D/middle/3D middle GFP (magenta).tif]

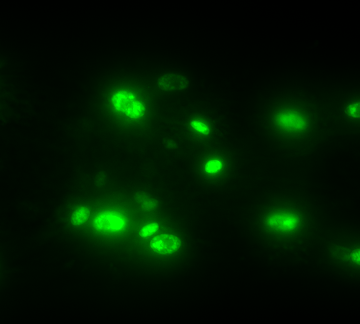

Supplement: Supplementary file 8 — Source Data Fig. 3 [file 44318_2024_49_MOESM8_ESM.zip › Figure 3/3D/left/3D left RFP (show as green on image).tif]

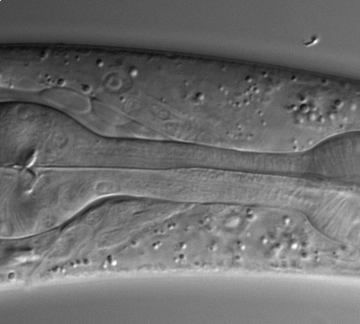

Supplement: Supplementary file 8 — Source Data Fig. 3 [file 44318_2024_49_MOESM8_ESM.zip › Figure 3/3D/left/3D left DIC.tif]

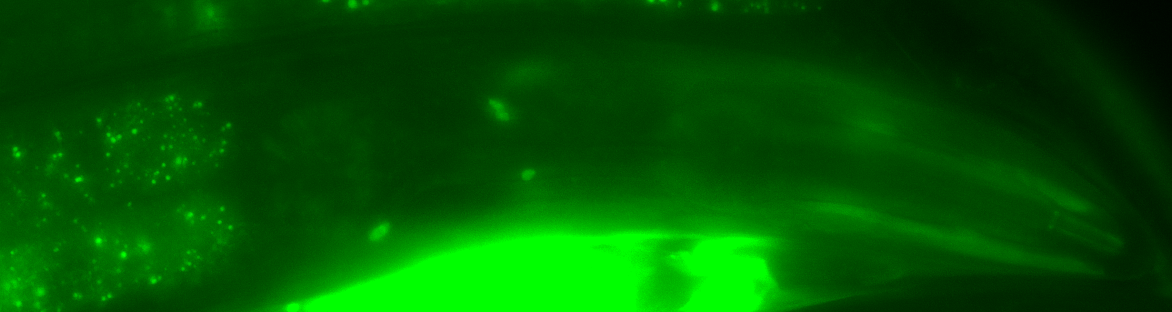

Supplement: Supplementary file 8 — Source Data Fig. 3 [file 44318_2024_49_MOESM8_ESM.zip › Figure 3/3A/right/3A right GFP.tif]

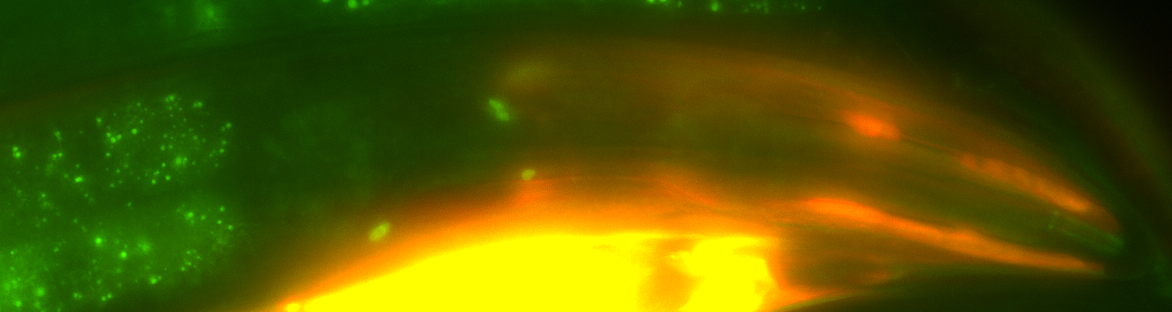

Supplement: Supplementary file 8 — Source Data Fig. 3 [file 44318_2024_49_MOESM8_ESM.zip › Figure 3/3A/right/3A right merge.tif]

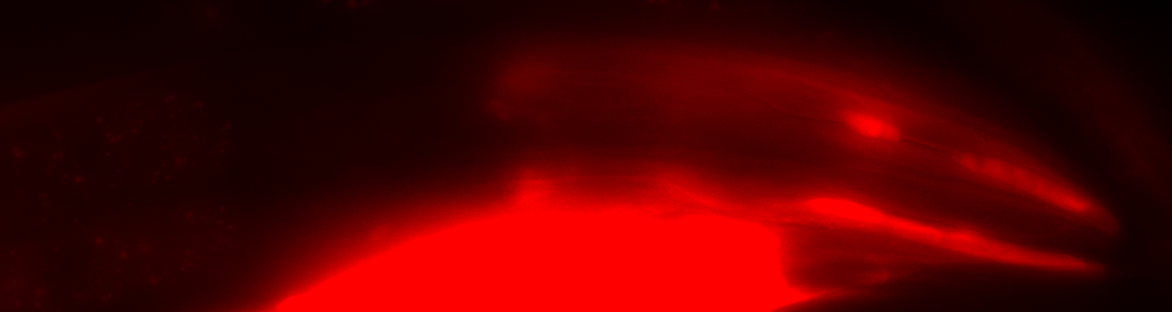

Supplement: Supplementary file 8 — Source Data Fig. 3 [file 44318_2024_49_MOESM8_ESM.zip › Figure 3/3A/right/3A right RFP (magenta).tif]

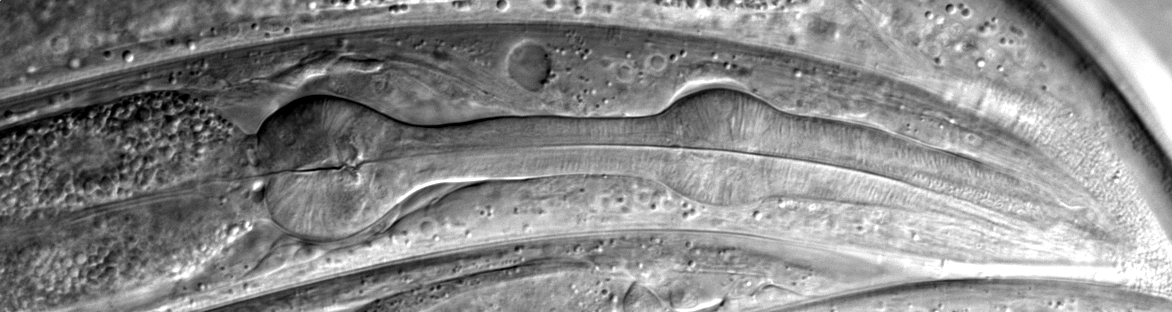

Supplement: Supplementary file 8 — Source Data Fig. 3 [file 44318_2024_49_MOESM8_ESM.zip › Figure 3/3A/right/3A right DIC.tif]

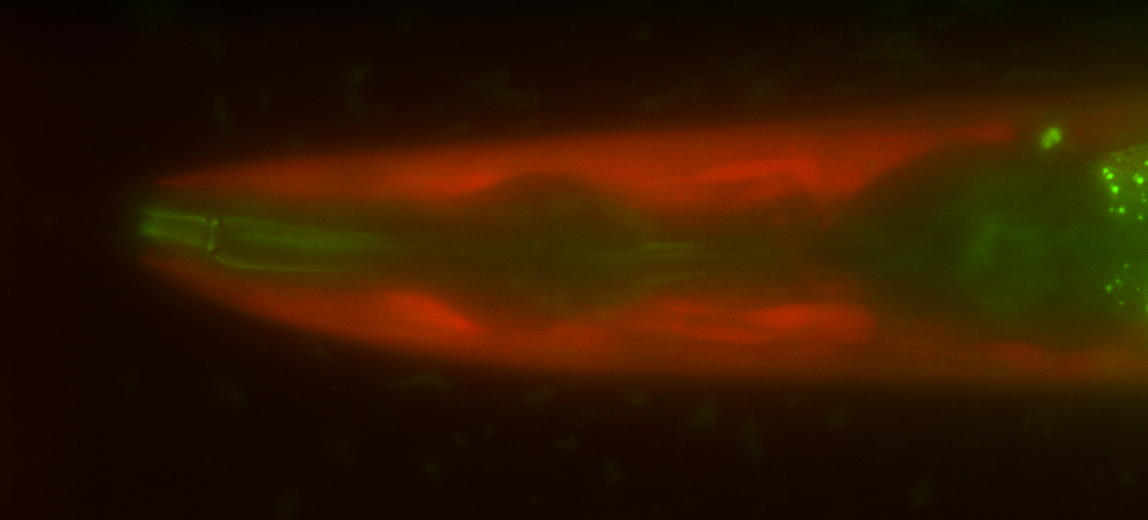

Supplement: Supplementary file 8 — Source Data Fig. 3 [file 44318_2024_49_MOESM8_ESM.zip › Figure 3/3A/middle/3A middle merge.tif]

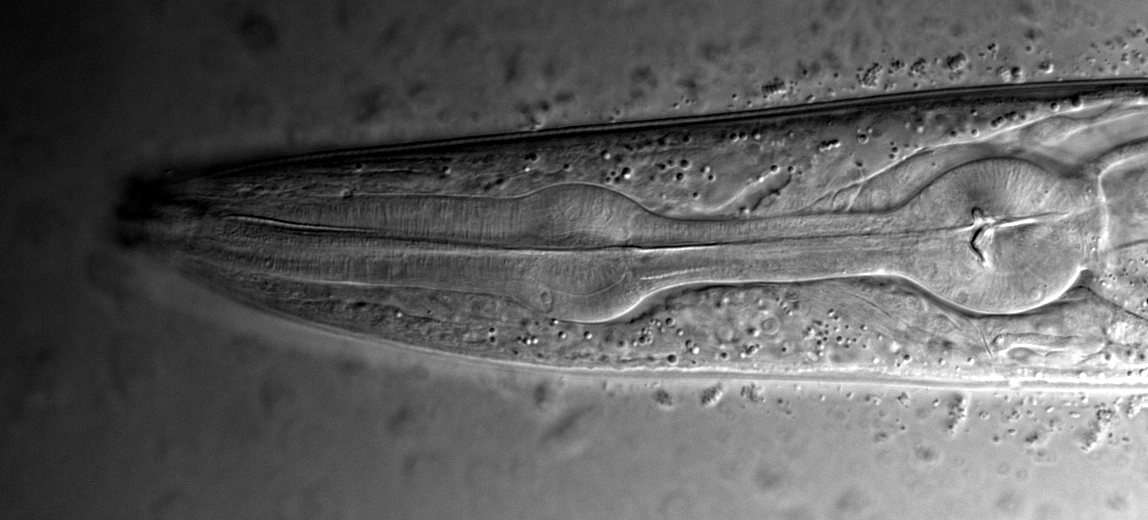

Supplement: Supplementary file 8 — Source Data Fig. 3 [file 44318_2024_49_MOESM8_ESM.zip › Figure 3/3A/middle/3A middle DIC.tif]

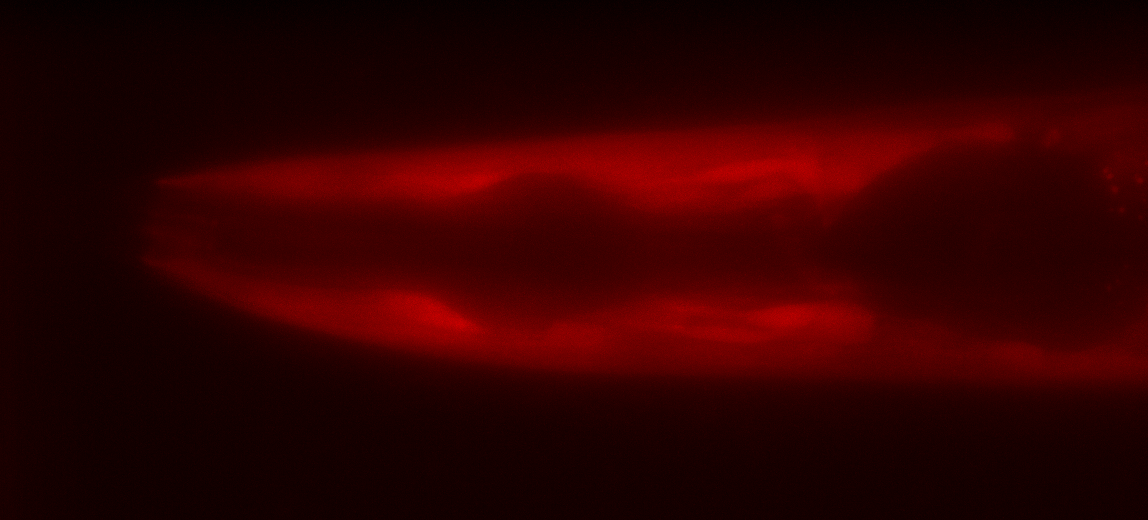

Supplement: Supplementary file 8 — Source Data Fig. 3 [file 44318_2024_49_MOESM8_ESM.zip › Figure 3/3A/middle/3A middle RFP (magenta).tif]

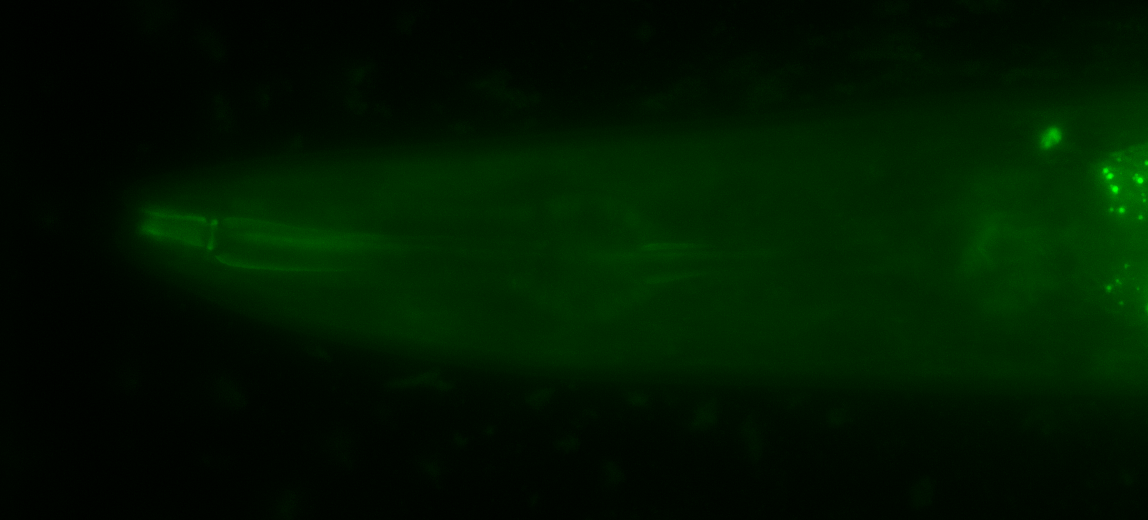

Supplement: Supplementary file 8 — Source Data Fig. 3 [file 44318_2024_49_MOESM8_ESM.zip › Figure 3/3A/middle/3A middle gfp.tif]

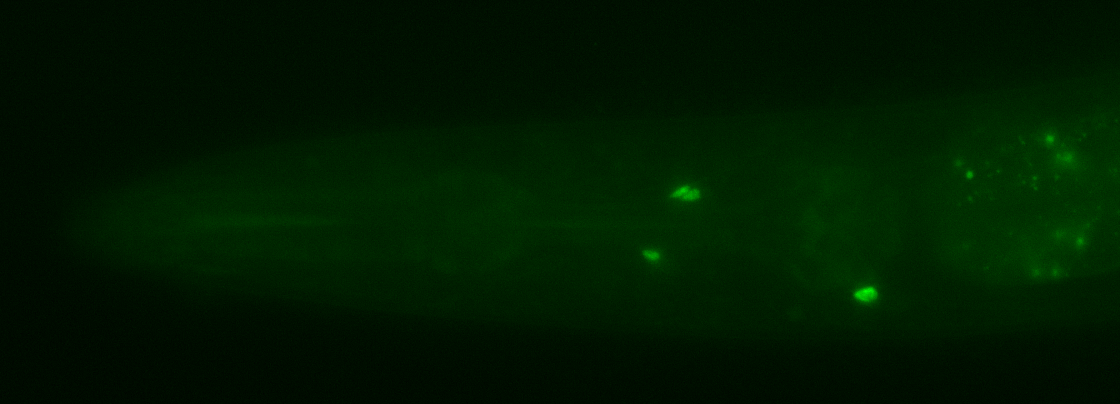

Supplement: Supplementary file 8 — Source Data Fig. 3 [file 44318_2024_49_MOESM8_ESM.zip › Figure 3/3A/left/3A left GFP.tif]

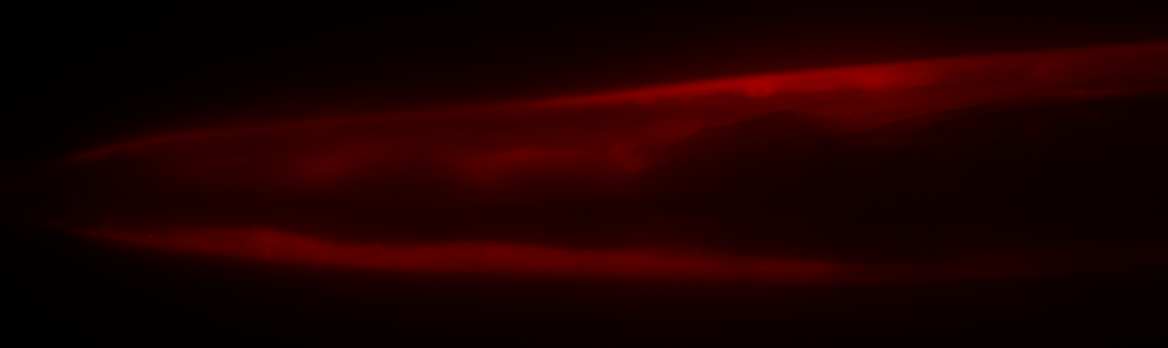

Supplement: Supplementary file 8 — Source Data Fig. 3 [file 44318_2024_49_MOESM8_ESM.zip › Figure 3/3F/middle/3F middle RFP (magenta).tif]

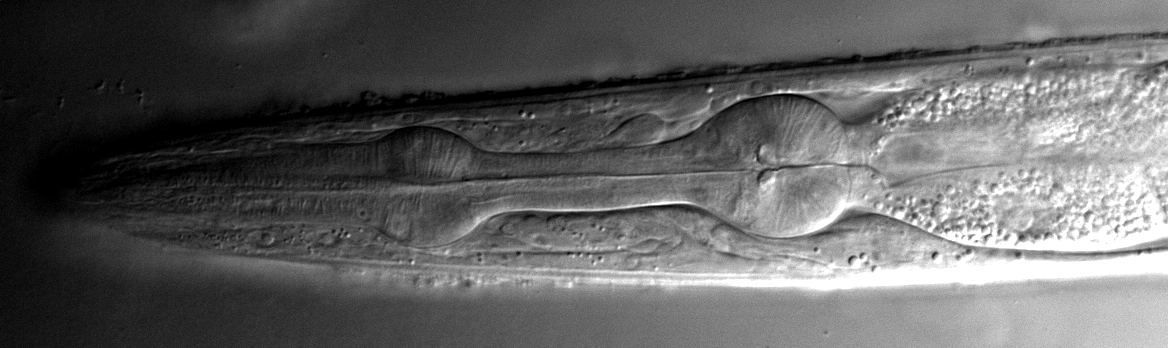

Supplement: Supplementary file 8 — Source Data Fig. 3 [file 44318_2024_49_MOESM8_ESM.zip › Figure 3/3F/middle/3F middle DIC.tif]

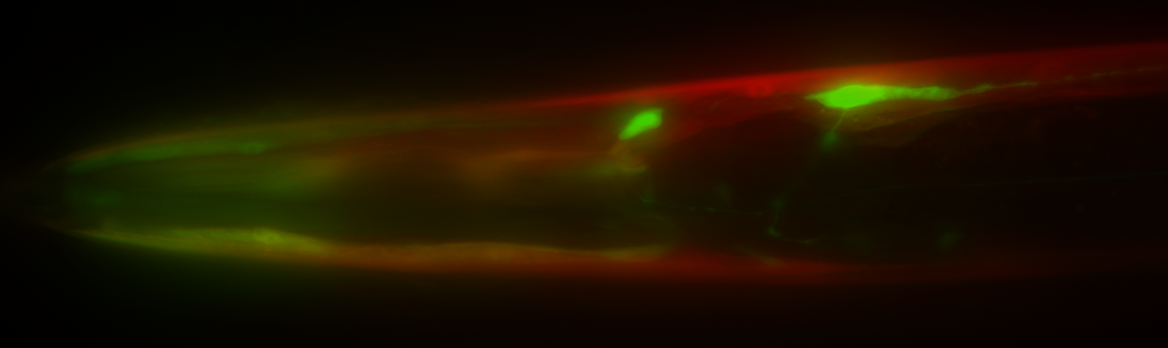

Supplement: Supplementary file 8 — Source Data Fig. 3 [file 44318_2024_49_MOESM8_ESM.zip › Figure 3/3F/middle/3F middle merge.tif]

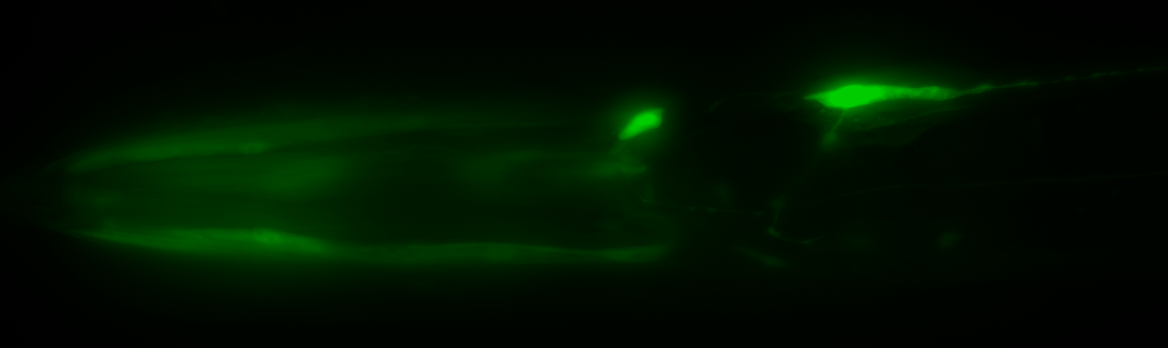

Supplement: Supplementary file 8 — Source Data Fig. 3 [file 44318_2024_49_MOESM8_ESM.zip › Figure 3/3F/middle/3F middle GFP.tif]

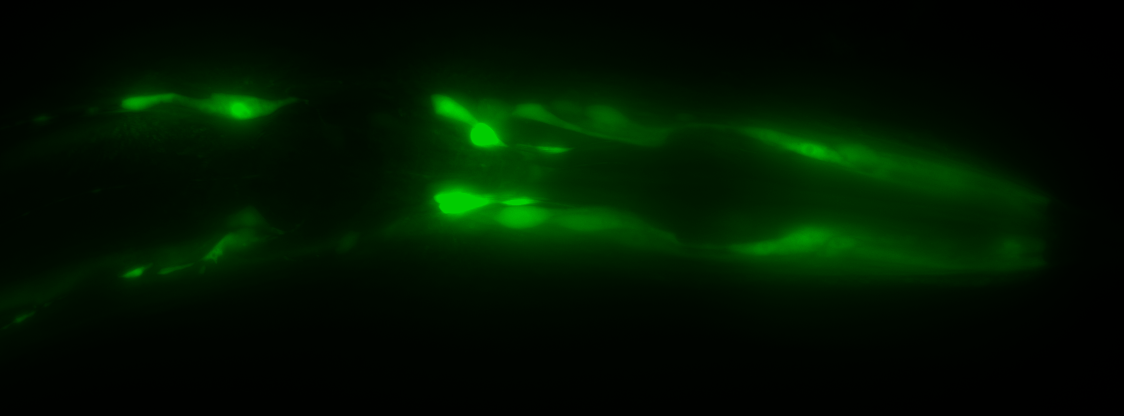

Supplement: Supplementary file 8 — Source Data Fig. 3 [file 44318_2024_49_MOESM8_ESM.zip › Figure 3/3F/left/3F left GFP.tif]

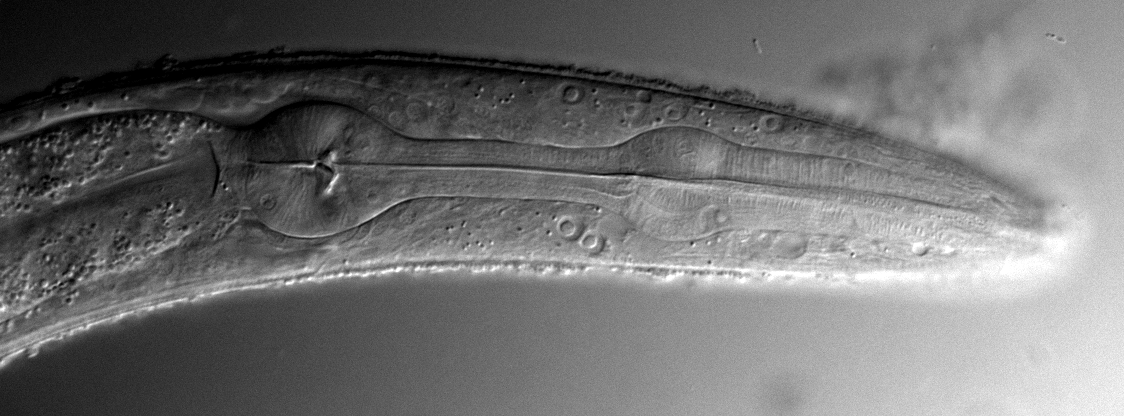

Supplement: Supplementary file 8 — Source Data Fig. 3 [file 44318_2024_49_MOESM8_ESM.zip › Figure 3/3F/left/3F left DIC.tif]

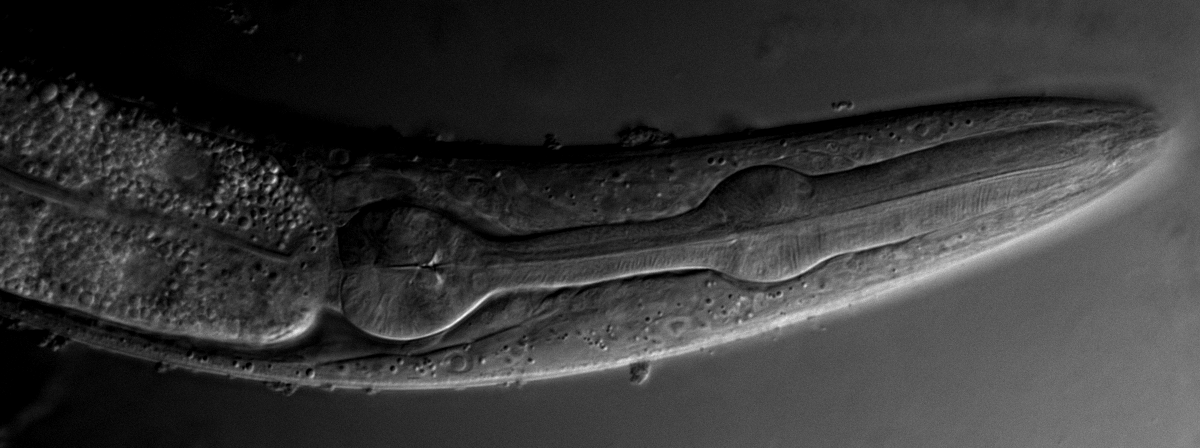

Supplement: Supplementary file 8 — Source Data Fig. 3 [file 44318_2024_49_MOESM8_ESM.zip › Figure 3/3G/right/3G right DIC.tif]

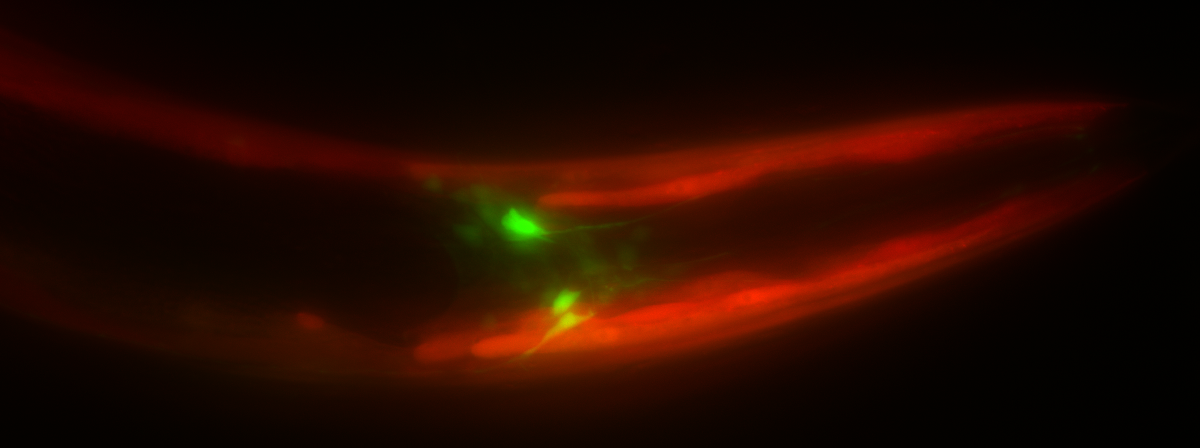

Supplement: Supplementary file 8 — Source Data Fig. 3 [file 44318_2024_49_MOESM8_ESM.zip › Figure 3/3G/right/3G right merge.tif]

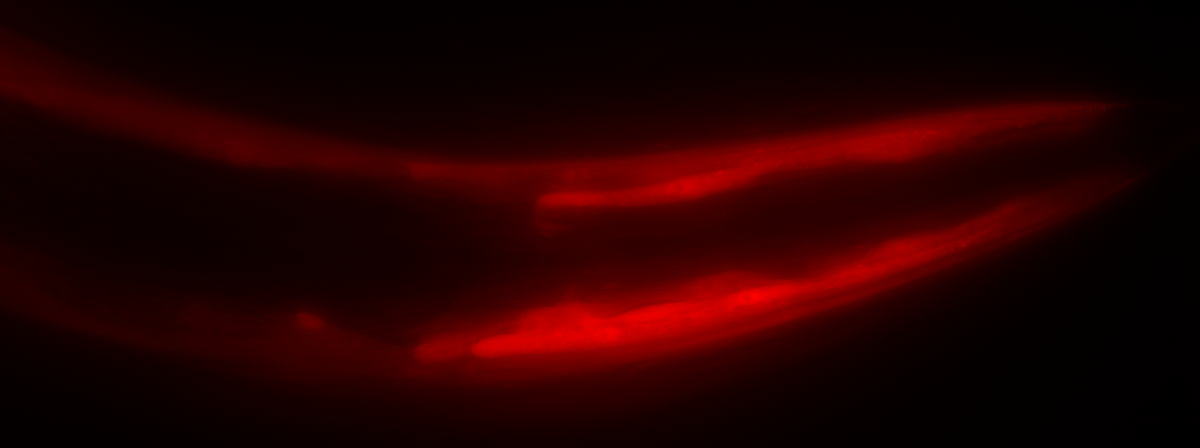

Supplement: Supplementary file 8 — Source Data Fig. 3 [file 44318_2024_49_MOESM8_ESM.zip › Figure 3/3G/right/3G right RFP (magenta).tif]

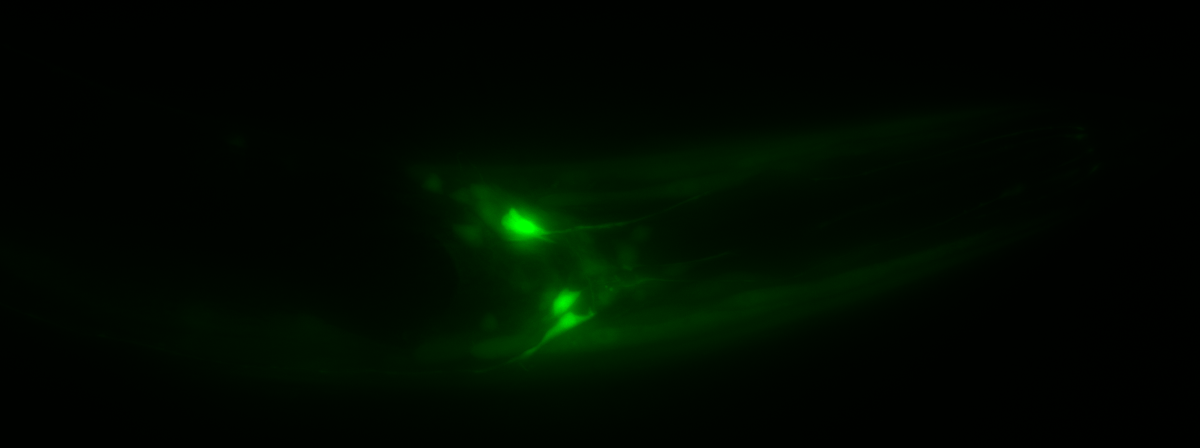

Supplement: Supplementary file 8 — Source Data Fig. 3 [file 44318_2024_49_MOESM8_ESM.zip › Figure 3/3G/right/3G right GFP.tif]

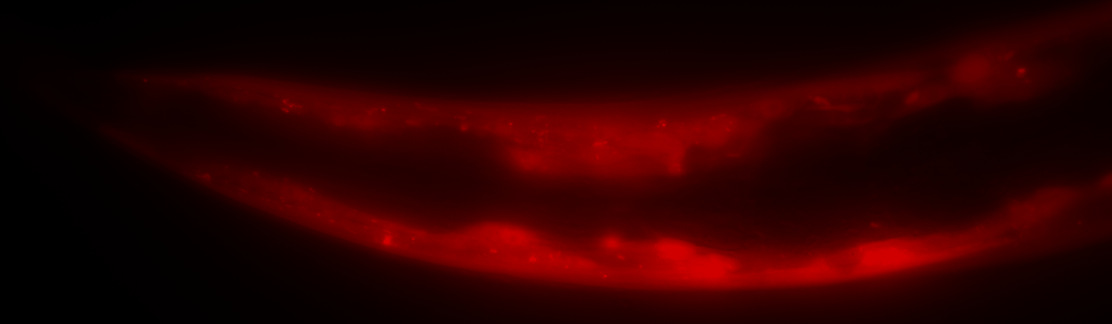

Supplement: Supplementary file 8 — Source Data Fig. 3 [file 44318_2024_49_MOESM8_ESM.zip › Figure 3/3G/middle/3G middle RFP (magenta).tif]

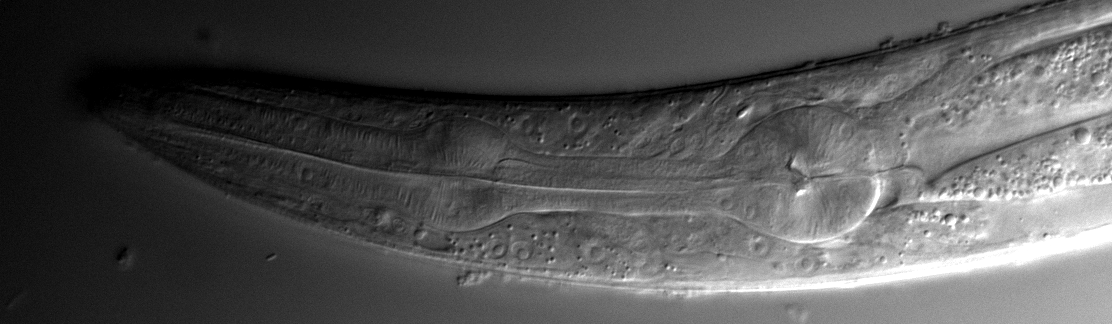

Supplement: Supplementary file 8 — Source Data Fig. 3 [file 44318_2024_49_MOESM8_ESM.zip › Figure 3/3G/middle/3G middle DIC.tif]

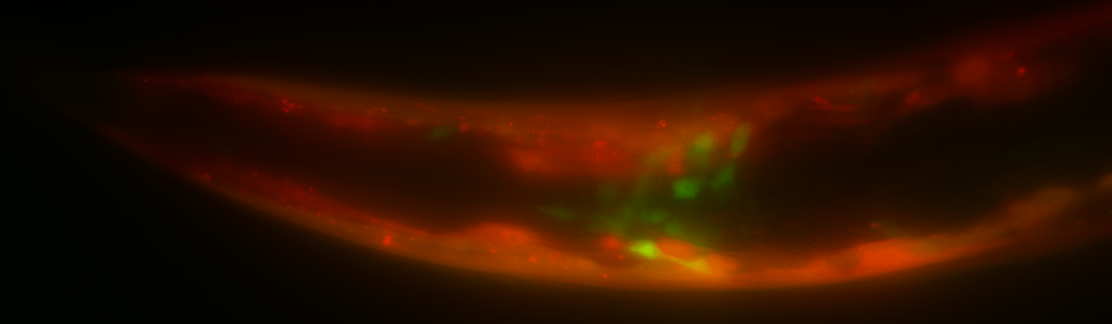

Supplement: Supplementary file 8 — Source Data Fig. 3 [file 44318_2024_49_MOESM8_ESM.zip › Figure 3/3G/middle/3G middle merge.tif]

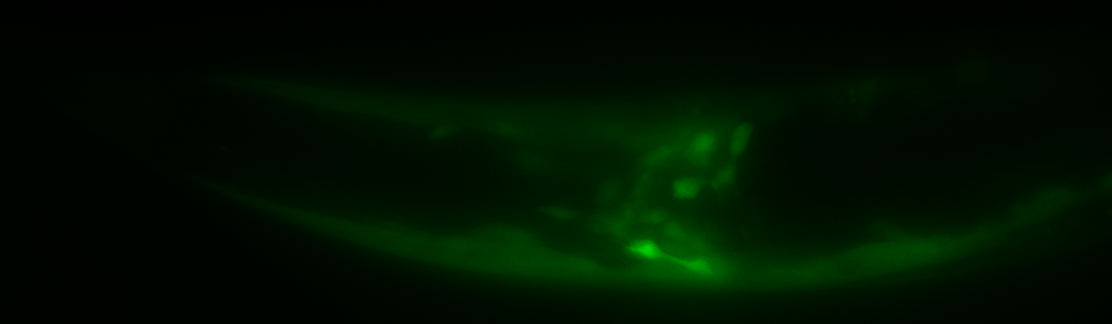

Supplement: Supplementary file 8 — Source Data Fig. 3 [file 44318_2024_49_MOESM8_ESM.zip › Figure 3/3G/middle/3G middle GFP.tif]

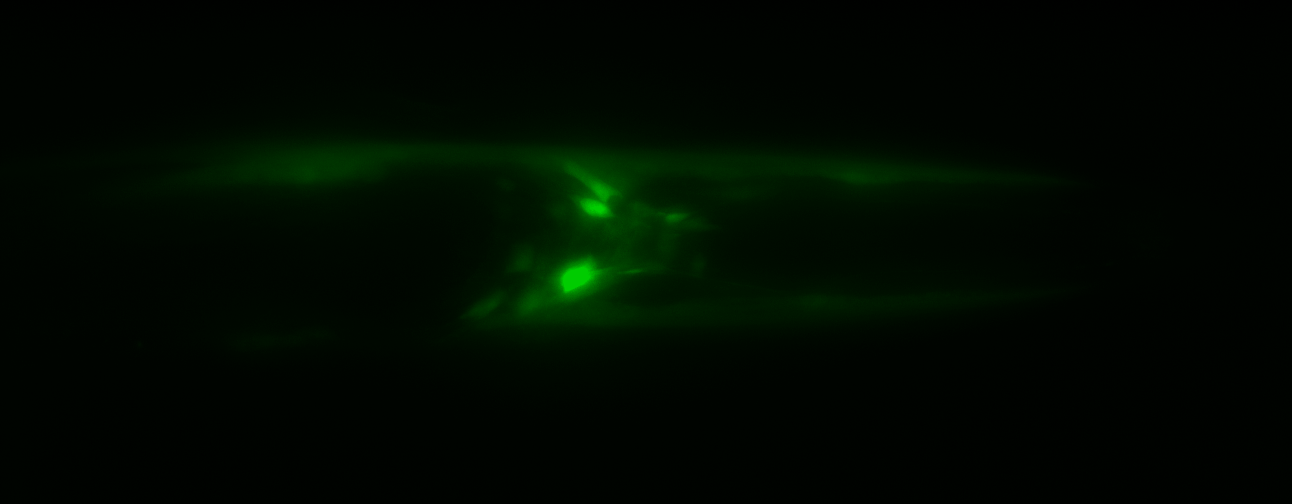

Supplement: Supplementary file 8 — Source Data Fig. 3 [file 44318_2024_49_MOESM8_ESM.zip › Figure 3/3G/left/3G left GFP.tif]

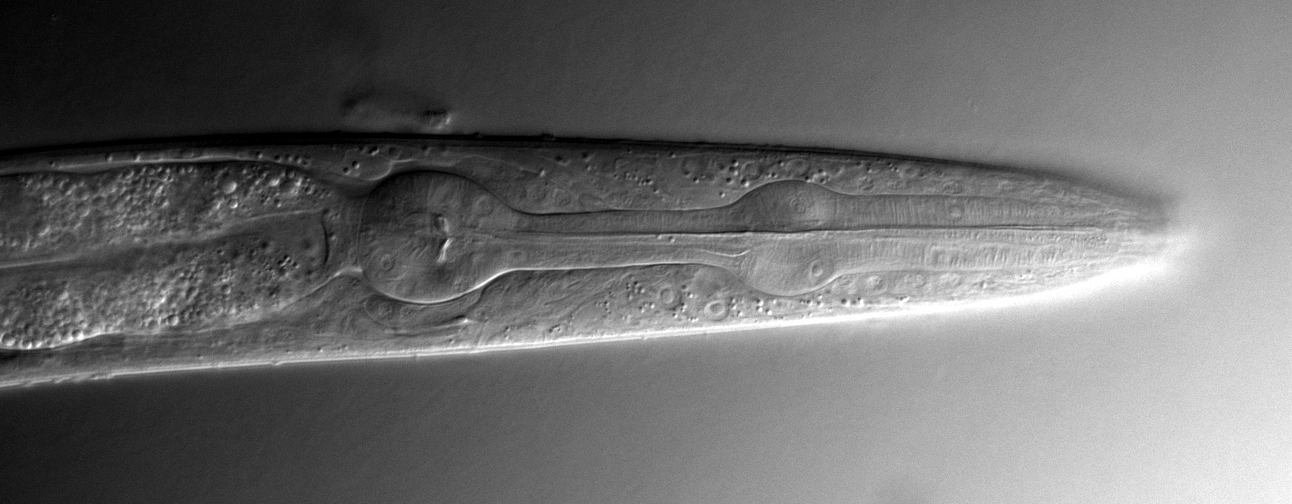

Supplement: Supplementary file 8 — Source Data Fig. 3 [file 44318_2024_49_MOESM8_ESM.zip › Figure 3/3G/left/3G left DIC.tif]

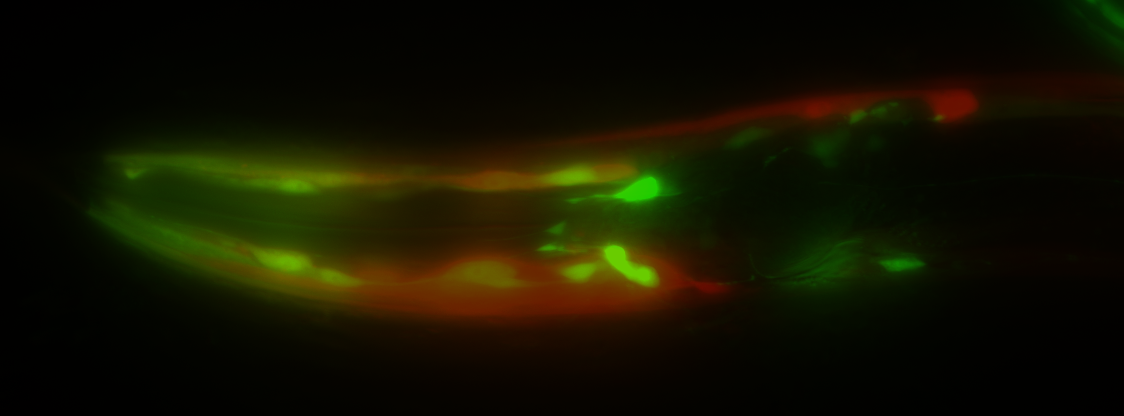

Supplement: Supplementary file 8 — Source Data Fig. 3 [file 44318_2024_49_MOESM8_ESM.zip › Figure 3/3F/right/right/3F right merge.tif]

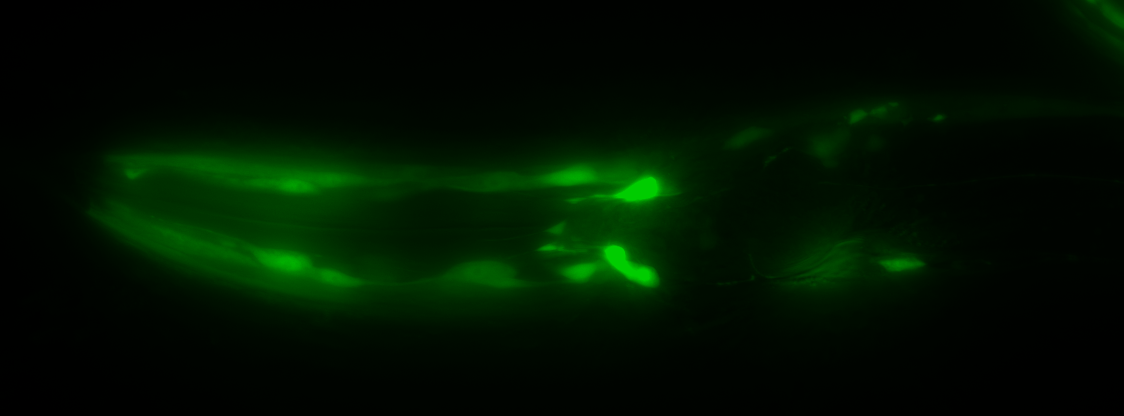

Supplement: Supplementary file 8 — Source Data Fig. 3 [file 44318_2024_49_MOESM8_ESM.zip › Figure 3/3F/right/right/3F right GFP.tif]

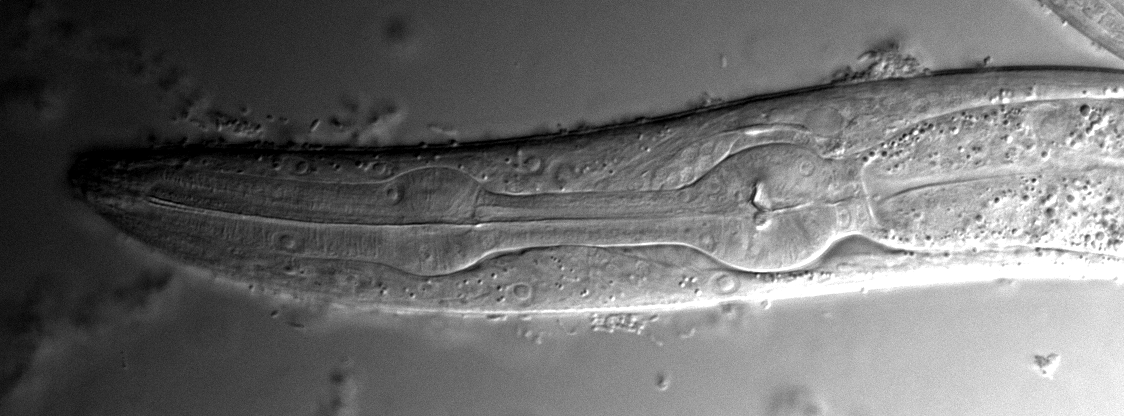

Supplement: Supplementary file 8 — Source Data Fig. 3 [file 44318_2024_49_MOESM8_ESM.zip › Figure 3/3F/right/right/3F right DIC.tif]

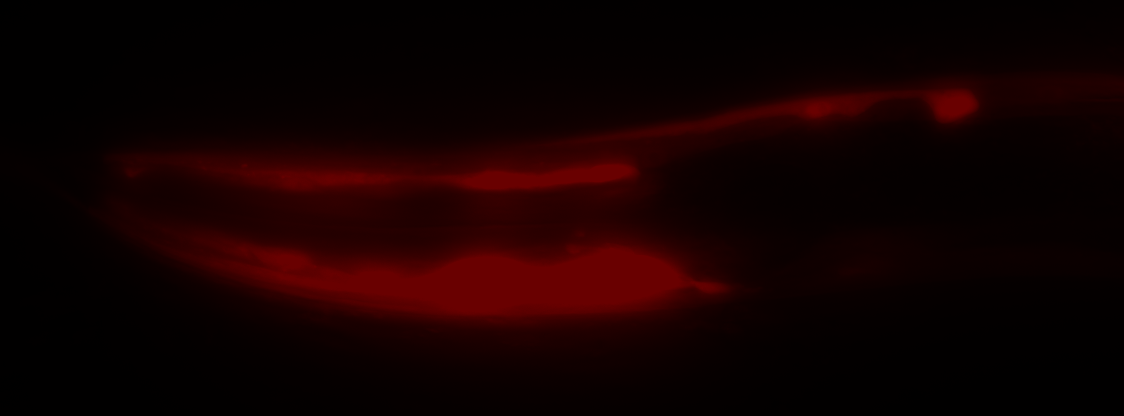

Supplement: Supplementary file 8 — Source Data Fig. 3 [file 44318_2024_49_MOESM8_ESM.zip › Figure 3/3F/right/right/3F right RFP (magenta).tif]

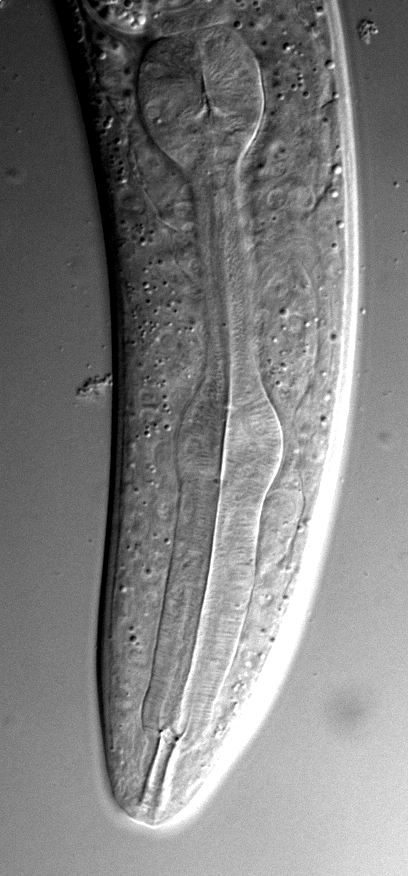

Supplement: Supplementary file 9 — Source Data Fig. 4 [file 44318_2024_49_MOESM9_ESM.zip › Figure 4/4C/+ K-NAA/DIC.tif]

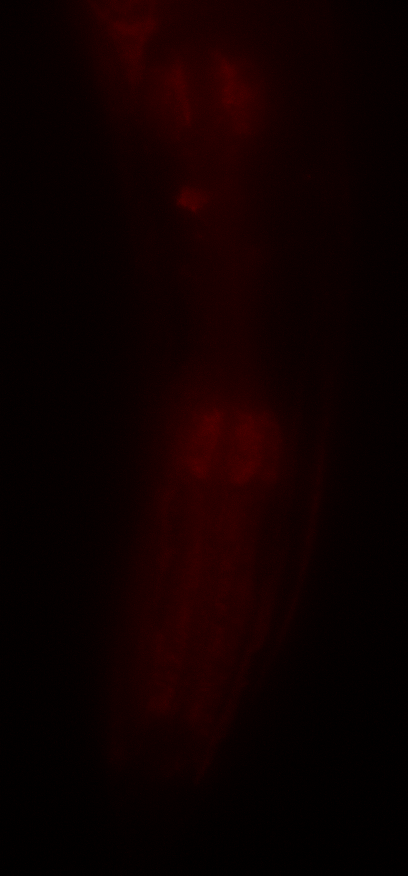

Supplement: Supplementary file 9 — Source Data Fig. 4 [file 44318_2024_49_MOESM9_ESM.zip › Figure 4/4C/+ K-NAA/RFP (magenta).tif]

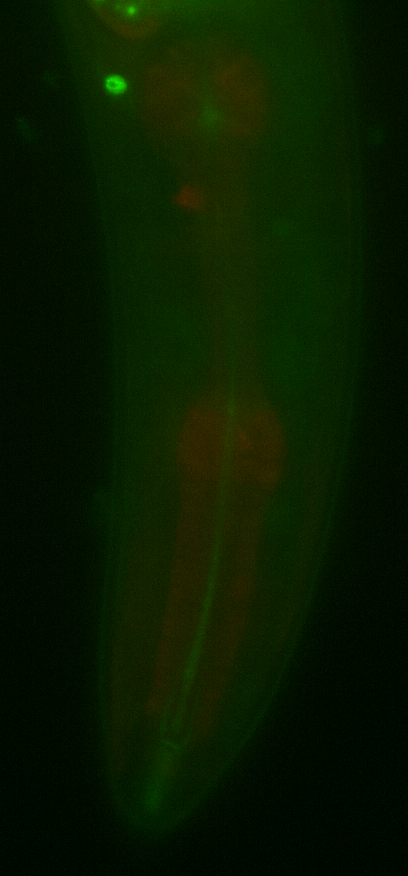

Supplement: Supplementary file 9 — Source Data Fig. 4 [file 44318_2024_49_MOESM9_ESM.zip › Figure 4/4C/+ K-NAA/GFP.tif]

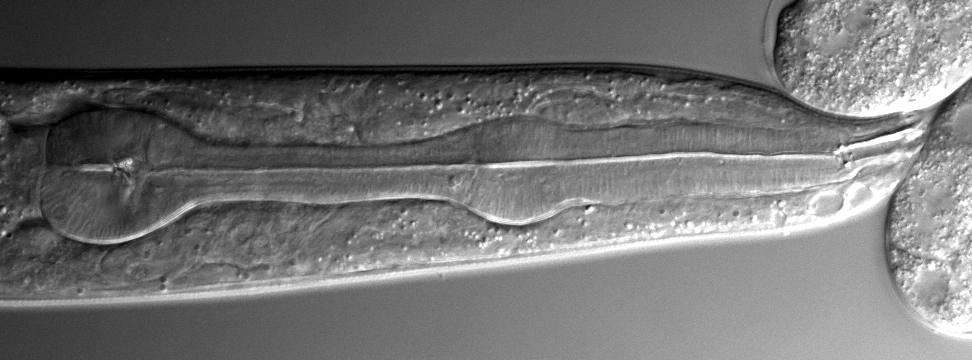

Supplement: Supplementary file 9 — Source Data Fig. 4 [file 44318_2024_49_MOESM9_ESM.zip › Figure 4/4C/no K-NAA/DIC.tif]

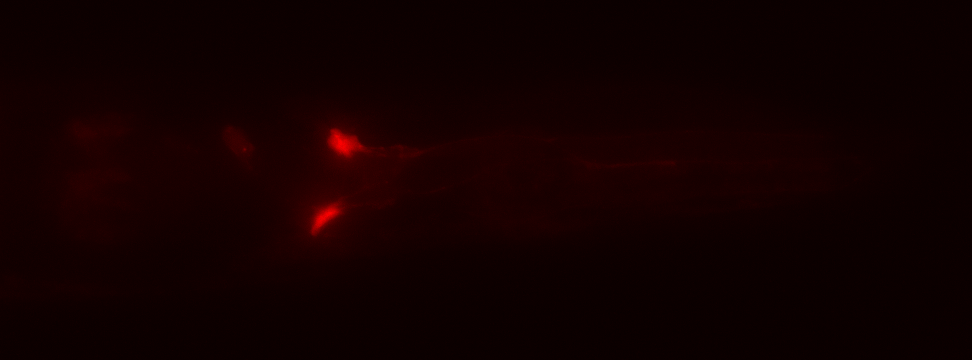

Supplement: Supplementary file 9 — Source Data Fig. 4 [file 44318_2024_49_MOESM9_ESM.zip › Figure 4/4C/no K-NAA/RFP (magenta).tif]

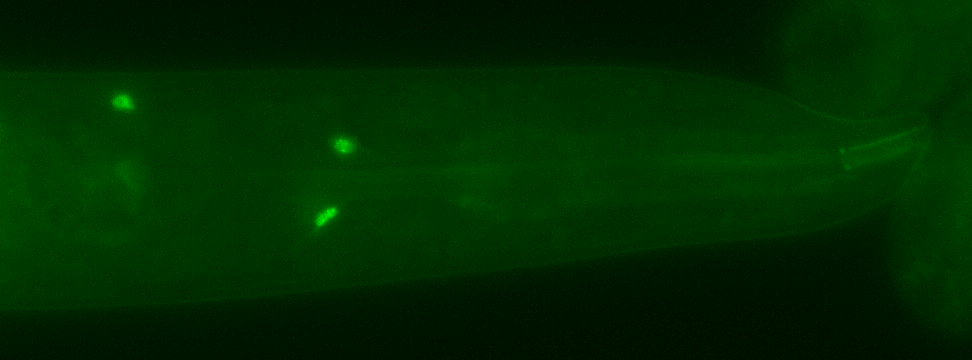

Supplement: Supplementary file 9 — Source Data Fig. 4 [file 44318_2024_49_MOESM9_ESM.zip › Figure 4/4C/no K-NAA/GFP.tif]

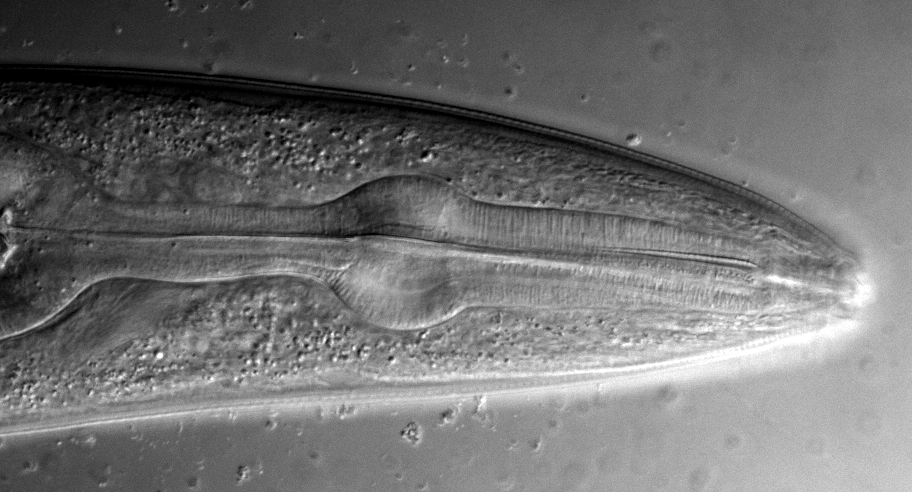

Supplement: Supplementary file 9 — Source Data Fig. 4 [file 44318_2024_49_MOESM9_ESM.zip › Figure 4/4D/+ K-NAA/DIC.tif]

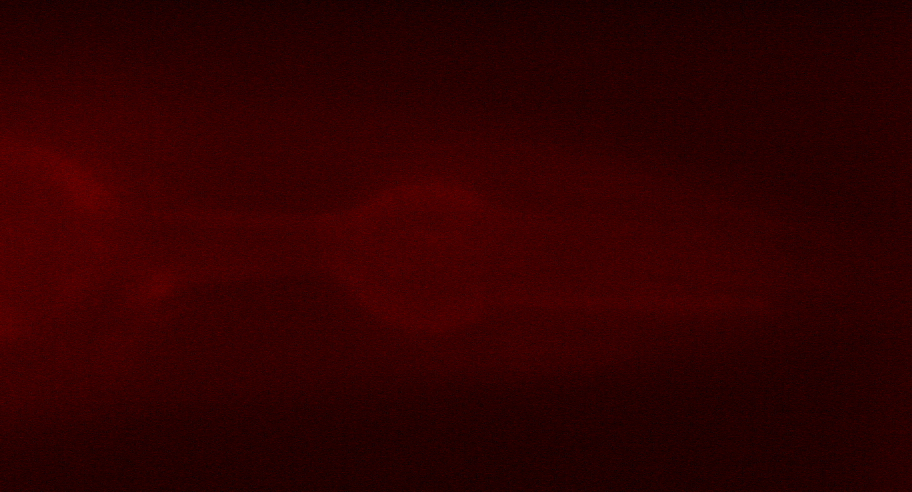

Supplement: Supplementary file 9 — Source Data Fig. 4 [file 44318_2024_49_MOESM9_ESM.zip › Figure 4/4D/+ K-NAA/RFP (magenta).tif]

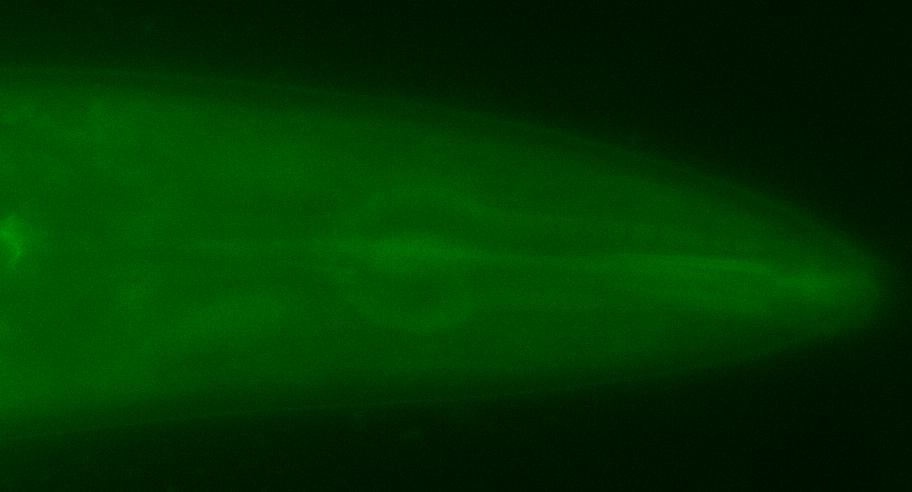

Supplement: Supplementary file 9 — Source Data Fig. 4 [file 44318_2024_49_MOESM9_ESM.zip › Figure 4/4D/+ K-NAA/GFP.tif]
