# Supplementary material for: LET-381/FoxF and its target UNC-30/Pitx2 specify and maintain the molecular identity of C. elegans mesodermal glia that regulate motor behavior
Source: EMBO J. 2024 Feb 15;43(6):4. doi: 10.1038/s44318-024-00049-w (PMC10943081; doi:10.1038/s44318-024-00049-w)
Supplement: Supplementary file 8 — Source Data Fig. 3 [file 44318_2024_49_MOESM8_ESM.zip › Figure 3/3D/right/README.rtf]

GFP channel is shown as red/magenta and RFP channel is shown as green for uniformity with the rest of the panels.
