# Supplementary material for: LET-381/FoxF and its target UNC-30/Pitx2 specify and maintain the molecular identity of C. elegans mesodermal glia that regulate motor behavior
Source: EMBO J. 2024 Feb 15;43(6):4. doi: 10.1038/s44318-024-00049-w (PMC10943081; doi:10.1038/s44318-024-00049-w)
Supplement: Supplementary file 8 — Source Data Fig. 3 [file 44318_2024_49_MOESM8_ESM.zip › Figure 3/3A/right/README.rtf]

The tail of a neighboring C. elegans animal (as clearly seen in the DIC channel) has very bright RFP expression, which is bleeding into the GFP channel. Therefore it was cropped out of the image shown on Figure 3A.
