# Supplementary figures and images for: LET-381/FoxF and its target UNC-30/Pitx2 specify and maintain the molecular identity of C. elegans mesodermal glia that regulate motor behavior (part 2 of 3)
Source: EMBO J. 2024 Feb 15;43(6):4. doi: 10.1038/s44318-024-00049-w (PMC10943081; doi:10.1038/s44318-024-00049-w)

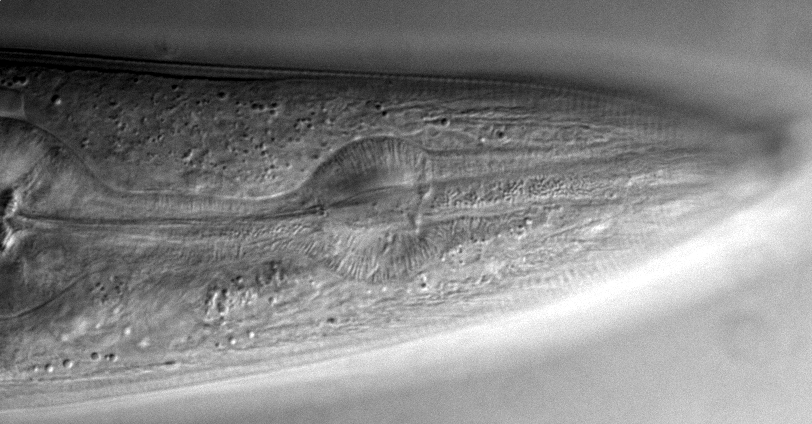

Supplement: Supplementary file 9 — Source Data Fig. 4 [file 44318_2024_49_MOESM9_ESM.zip › Figure 4/4D/no K-NAA/DIC.tif]

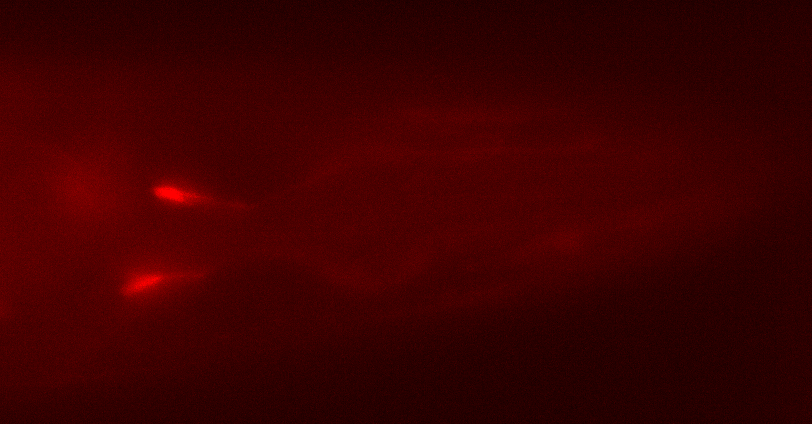

Supplement: Supplementary file 9 — Source Data Fig. 4 [file 44318_2024_49_MOESM9_ESM.zip › Figure 4/4D/no K-NAA/RFP (magenta).tif]

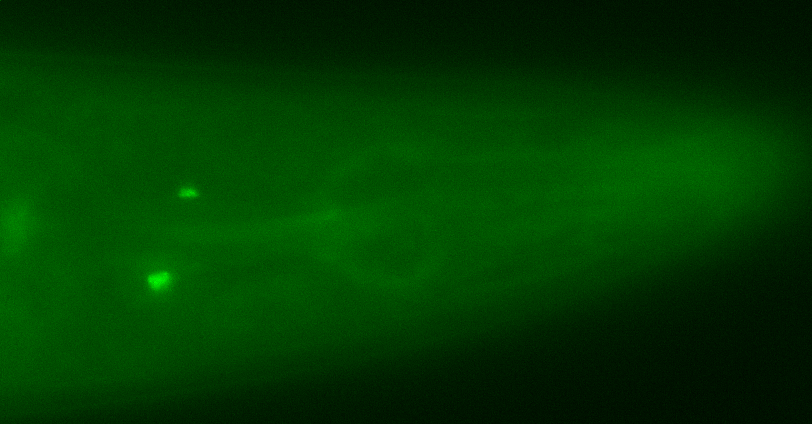

Supplement: Supplementary file 9 — Source Data Fig. 4 [file 44318_2024_49_MOESM9_ESM.zip › Figure 4/4D/no K-NAA/GFP.tif]

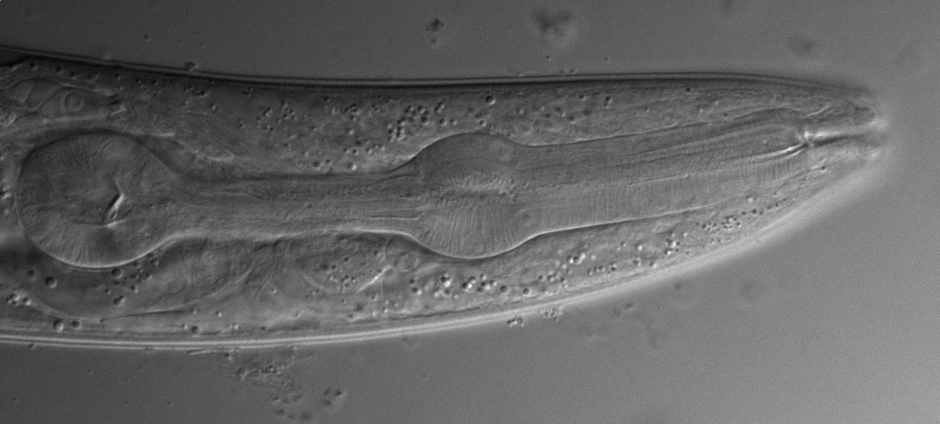

Supplement: Supplementary file 10 — Source Data Fig. 5 [file 44318_2024_49_MOESM10_ESM.zip › Figure 5/5A/left (wild type)/DIC.tif]

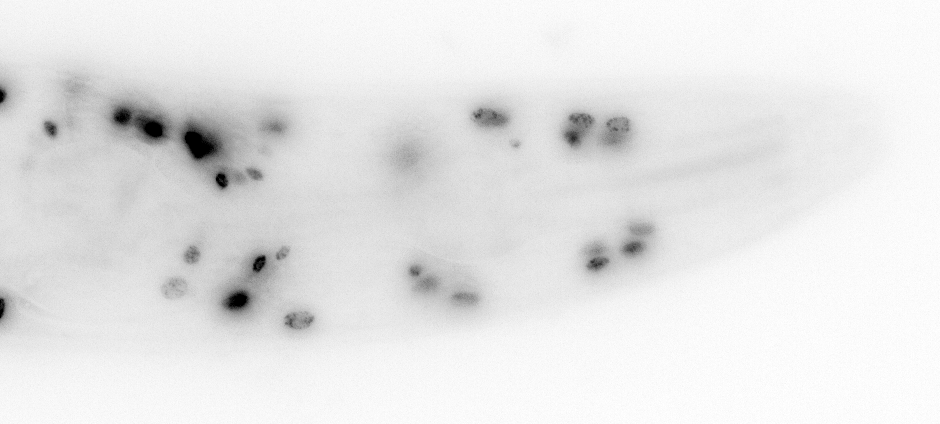

Supplement: Supplementary file 10 — Source Data Fig. 5 [file 44318_2024_49_MOESM10_ESM.zip › Figure 5/5A/left (wild type)/GFP.tif]

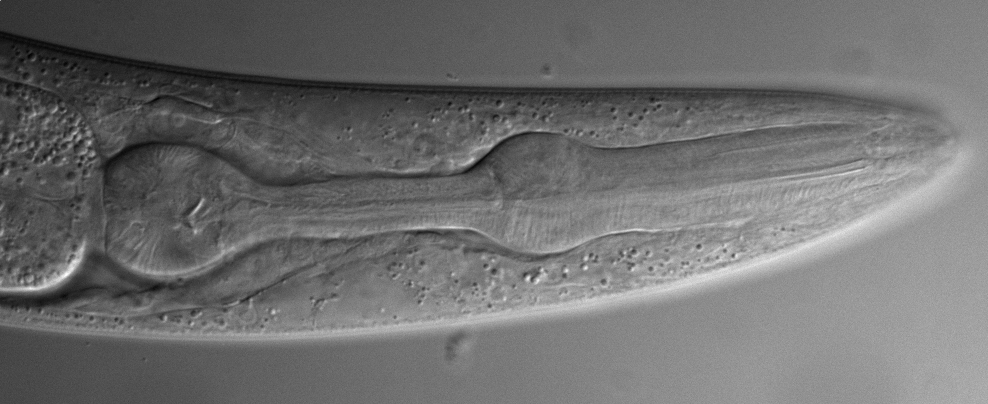

Supplement: Supplementary file 10 — Source Data Fig. 5 [file 44318_2024_49_MOESM10_ESM.zip › Figure 5/5A/right (let-381 motif mutated)/DIC.tif]

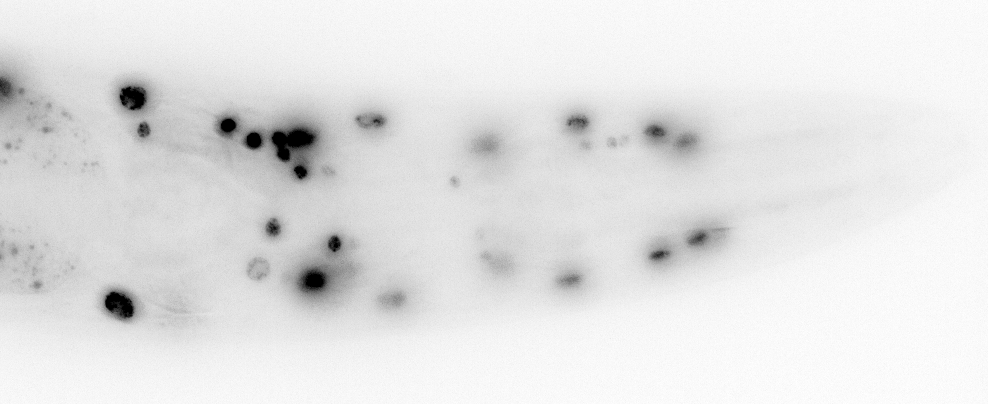

Supplement: Supplementary file 10 — Source Data Fig. 5 [file 44318_2024_49_MOESM10_ESM.zip › Figure 5/5A/right (let-381 motif mutated)/GFP.tif]

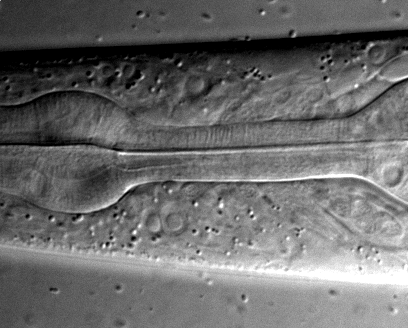

Supplement: Supplementary file 10 — Source Data Fig. 5 [file 44318_2024_49_MOESM10_ESM.zip › Figure 5/5C/left (wild type)/DIC.tif]

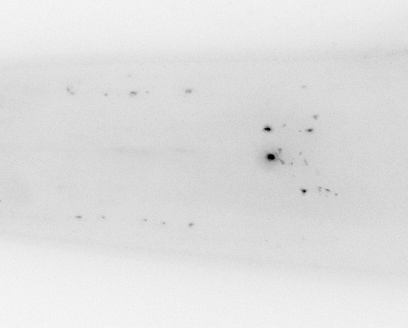

Supplement: Supplementary file 10 — Source Data Fig. 5 [file 44318_2024_49_MOESM10_ESM.zip › Figure 5/5C/left (wild type)/GFP.tif]

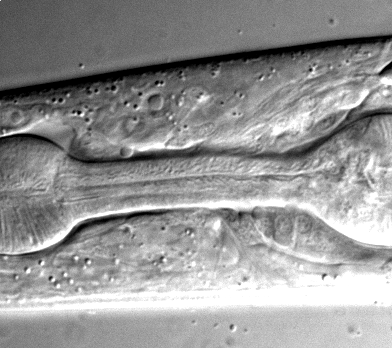

Supplement: Supplementary file 10 — Source Data Fig. 5 [file 44318_2024_49_MOESM10_ESM.zip › Figure 5/5C/right (let-381 motif mutated)/DIC.tif]

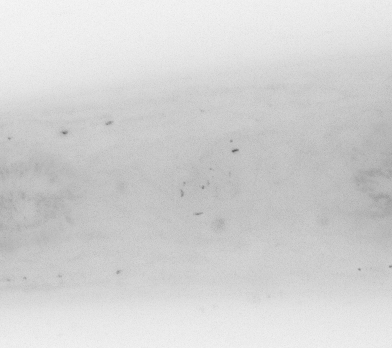

Supplement: Supplementary file 10 — Source Data Fig. 5 [file 44318_2024_49_MOESM10_ESM.zip › Figure 5/5C/right (let-381 motif mutated)/GFP.tif]

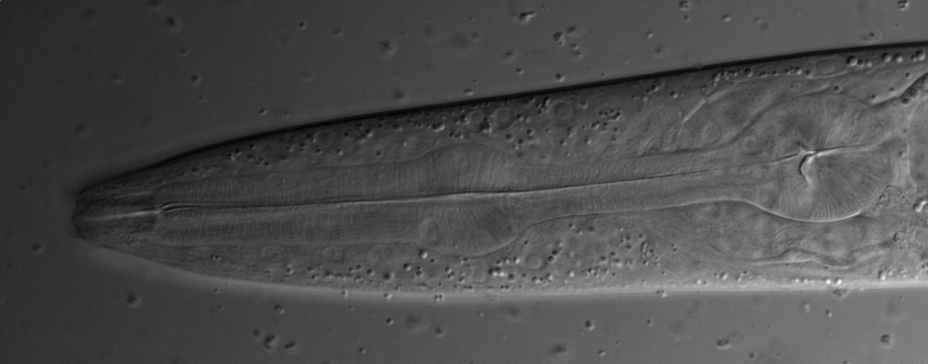

Supplement: Supplementary file 10 — Source Data Fig. 5 [file 44318_2024_49_MOESM10_ESM.zip › Figure 5/5D/bottom right (both let-381 motifs mutated)/DIC.tif]

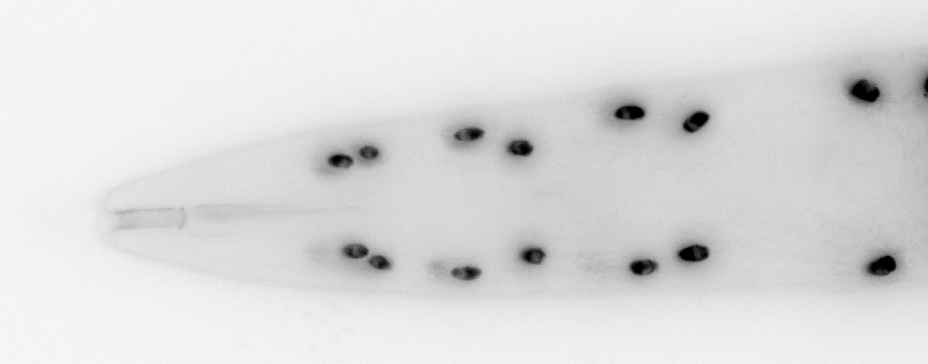

Supplement: Supplementary file 10 — Source Data Fig. 5 [file 44318_2024_49_MOESM10_ESM.zip › Figure 5/5D/bottom right (both let-381 motifs mutated)/GFP.tif]

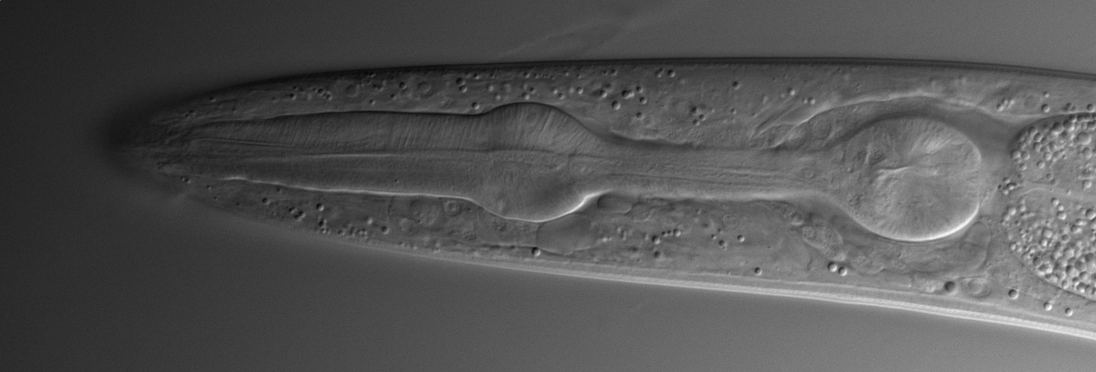

Supplement: Supplementary file 10 — Source Data Fig. 5 [file 44318_2024_49_MOESM10_ESM.zip › Figure 5/5D/bottom left (2nd let-381 motif mutated)/DIC.tif]

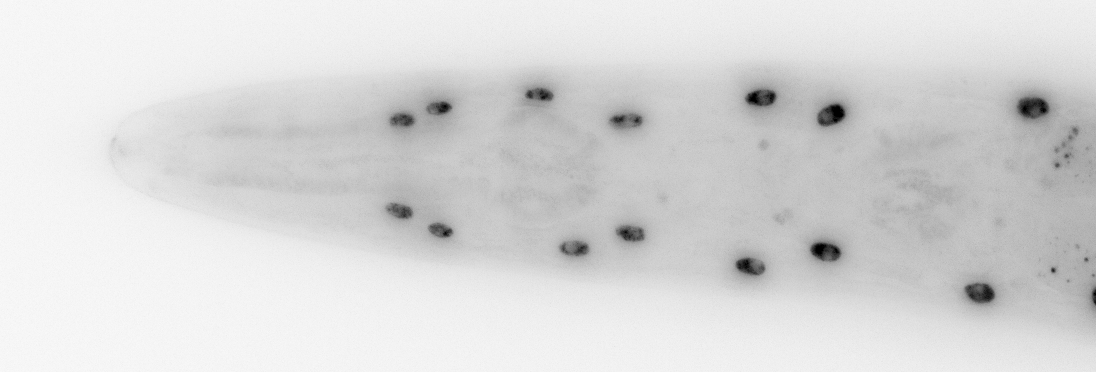

Supplement: Supplementary file 10 — Source Data Fig. 5 [file 44318_2024_49_MOESM10_ESM.zip › Figure 5/5D/bottom left (2nd let-381 motif mutated)/GFP.tif]

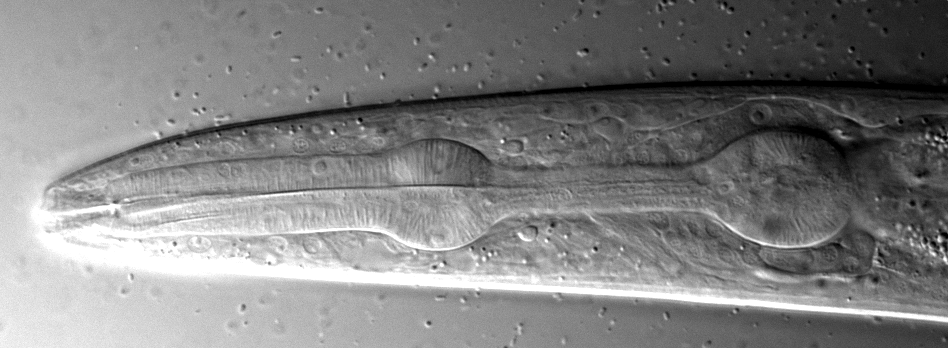

Supplement: Supplementary file 10 — Source Data Fig. 5 [file 44318_2024_49_MOESM10_ESM.zip › Figure 5/5D/left (wild type)/DIC.tif]

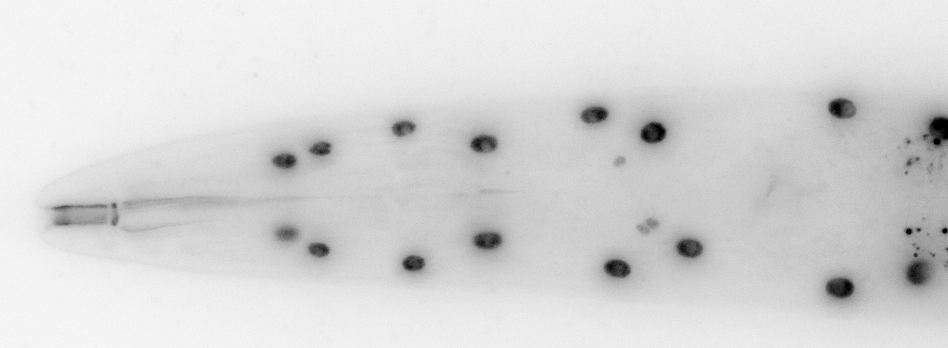

Supplement: Supplementary file 10 — Source Data Fig. 5 [file 44318_2024_49_MOESM10_ESM.zip › Figure 5/5D/left (wild type)/GFP.tif]

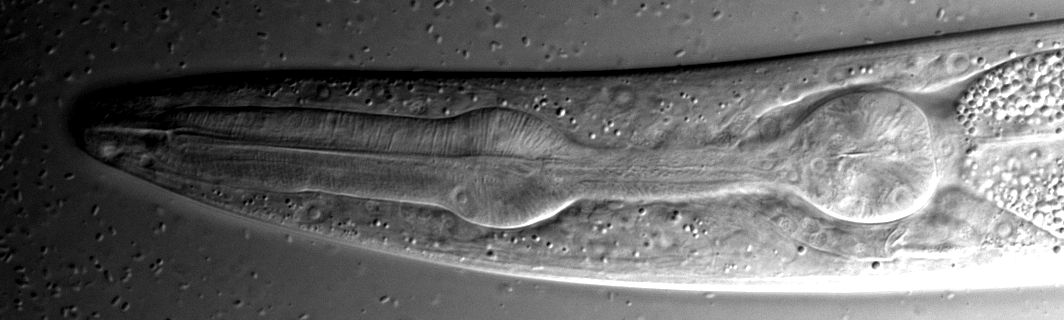

Supplement: Supplementary file 10 — Source Data Fig. 5 [file 44318_2024_49_MOESM10_ESM.zip › Figure 5/5D/top right (1st wild type motif mutated)/DIC.tif]

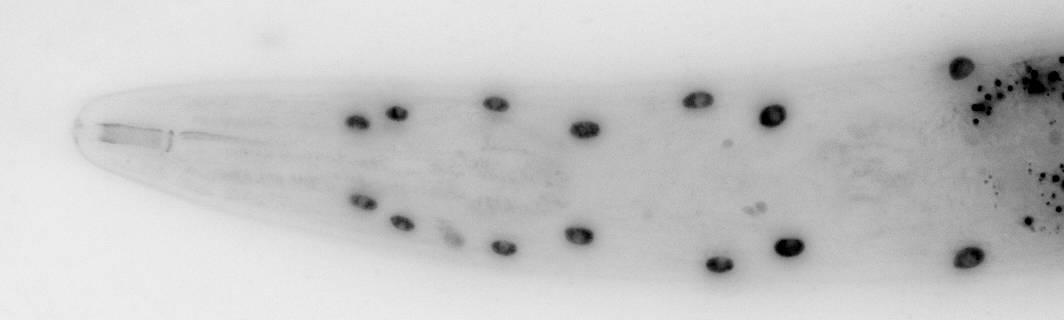

Supplement: Supplementary file 10 — Source Data Fig. 5 [file 44318_2024_49_MOESM10_ESM.zip › Figure 5/5D/top right (1st wild type motif mutated)/GFP.tif]

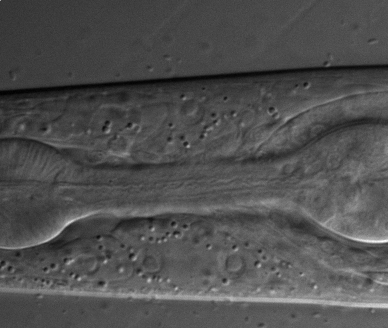

Supplement: Supplementary file 10 — Source Data Fig. 5 [file 44318_2024_49_MOESM10_ESM.zip › Figure 5/5B/left (wild type)/DIC.tif]

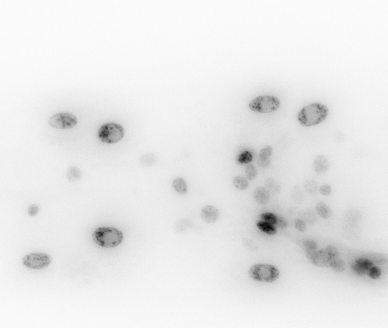

Supplement: Supplementary file 10 — Source Data Fig. 5 [file 44318_2024_49_MOESM10_ESM.zip › Figure 5/5B/left (wild type)/GFP.tif]

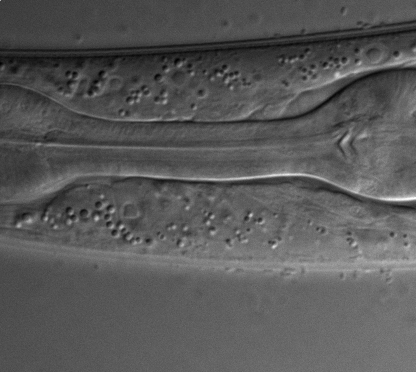

Supplement: Supplementary file 10 — Source Data Fig. 5 [file 44318_2024_49_MOESM10_ESM.zip › Figure 5/5B/right (let-381 motif mutated)/DIC.tif]

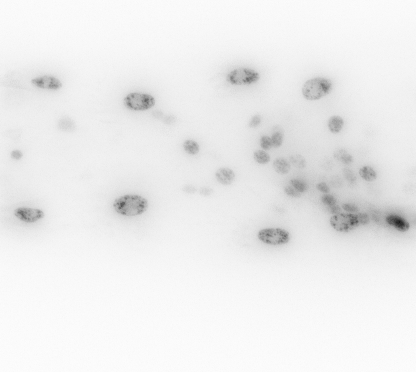

Supplement: Supplementary file 10 — Source Data Fig. 5 [file 44318_2024_49_MOESM10_ESM.zip › Figure 5/5B/right (let-381 motif mutated)/GFP.tif]

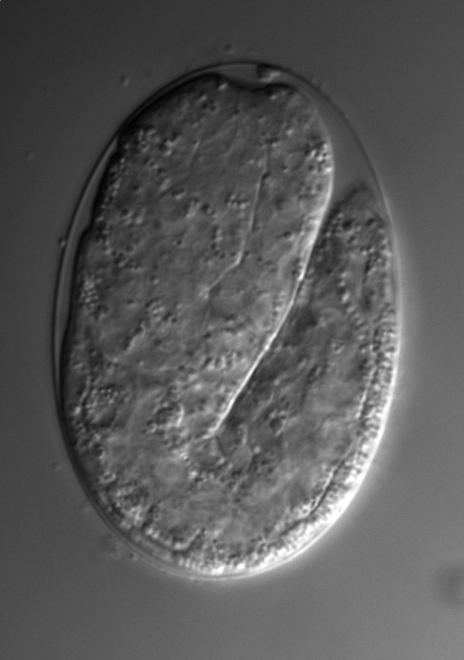

Supplement: Supplementary file 11 — Source Data Fig. 6 [file 44318_2024_49_MOESM11_ESM.zip › Figure 6/6A/Top Row/2fold/DIC.tif]

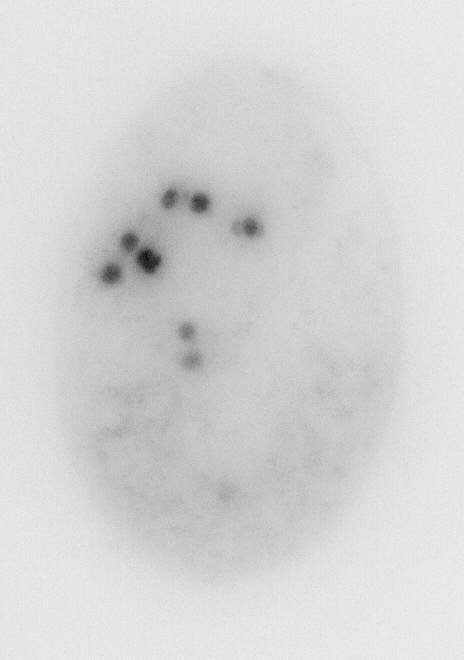

Supplement: Supplementary file 11 — Source Data Fig. 6 [file 44318_2024_49_MOESM11_ESM.zip › Figure 6/6A/Top Row/2fold/GFP.tif]

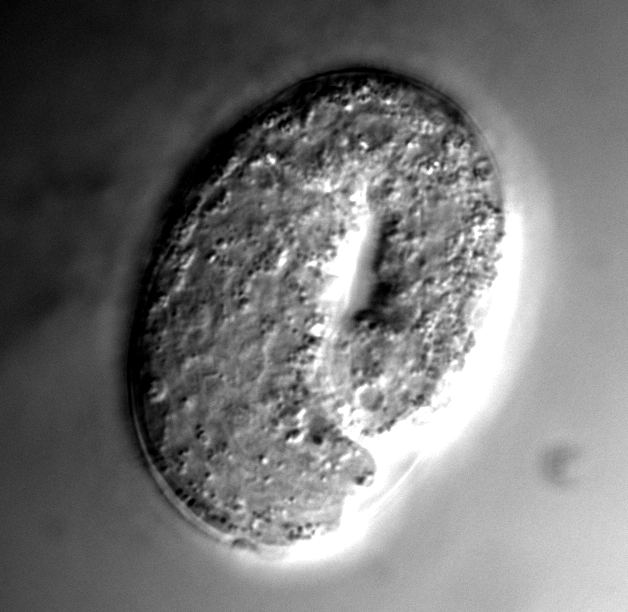

Supplement: Supplementary file 11 — Source Data Fig. 6 [file 44318_2024_49_MOESM11_ESM.zip › Figure 6/6A/Top Row/3fold/DIC.tif]

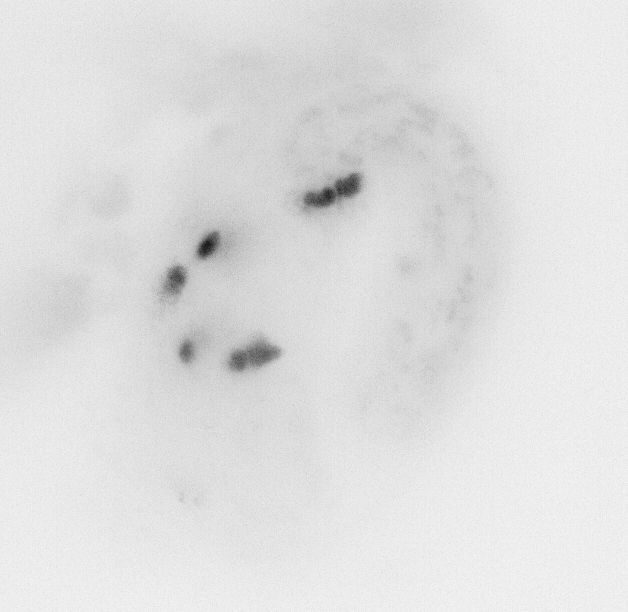

Supplement: Supplementary file 11 — Source Data Fig. 6 [file 44318_2024_49_MOESM11_ESM.zip › Figure 6/6A/Top Row/3fold/GFP.tif]

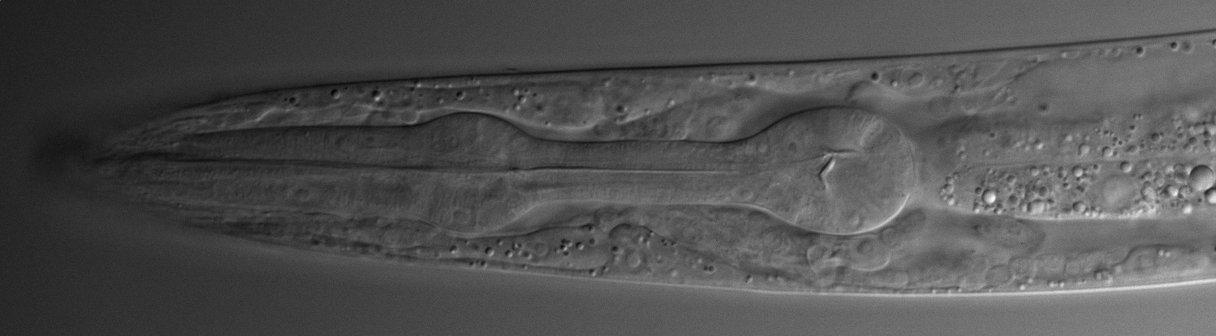

Supplement: Supplementary file 11 — Source Data Fig. 6 [file 44318_2024_49_MOESM11_ESM.zip › Figure 6/6A/Top Row/L4/DIC.tif]

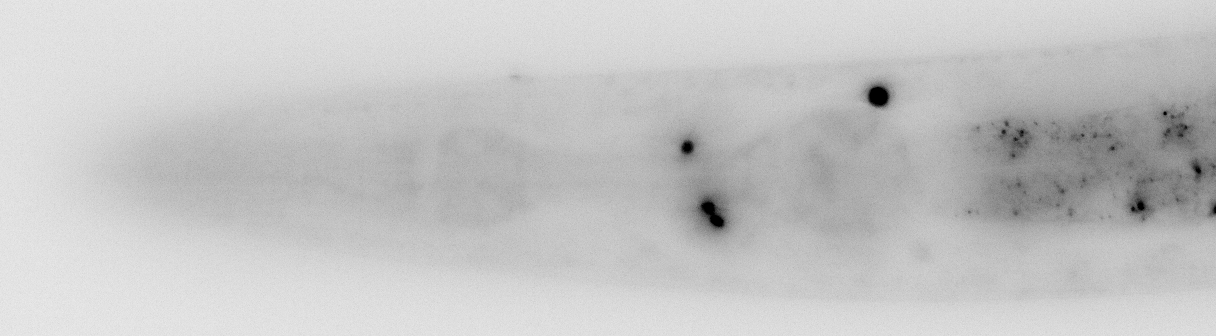

Supplement: Supplementary file 11 — Source Data Fig. 6 [file 44318_2024_49_MOESM11_ESM.zip › Figure 6/6A/Top Row/L4/GFP.tif]

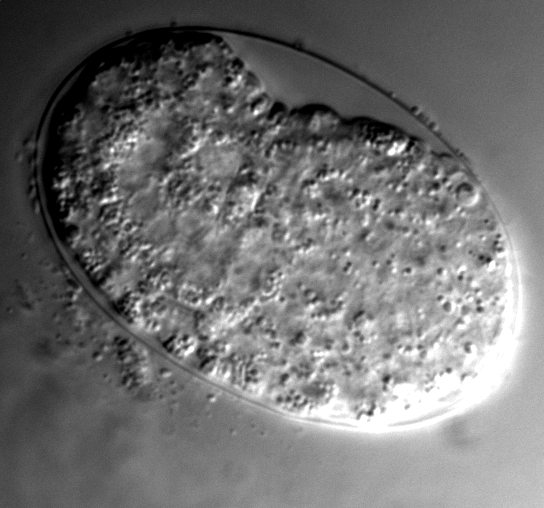

Supplement: Supplementary file 11 — Source Data Fig. 6 [file 44318_2024_49_MOESM11_ESM.zip › Figure 6/6A/Top Row/Bean/DIC.tif]

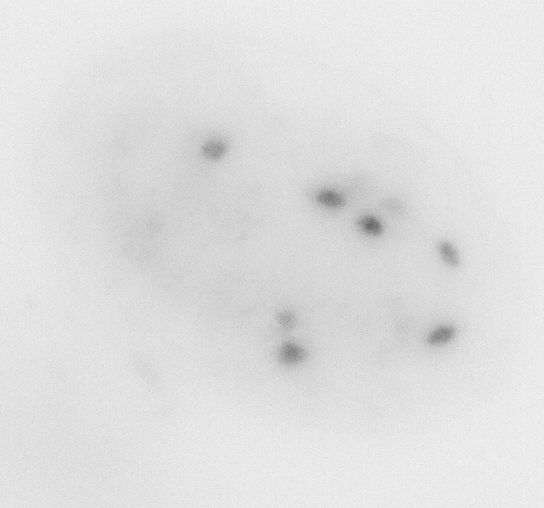

Supplement: Supplementary file 11 — Source Data Fig. 6 [file 44318_2024_49_MOESM11_ESM.zip › Figure 6/6A/Top Row/Bean/GFP.tif]

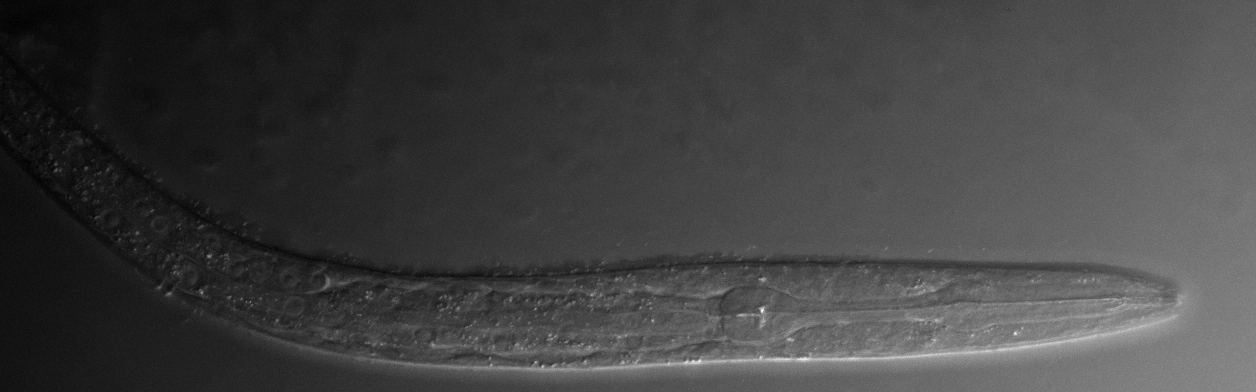

Supplement: Supplementary file 11 — Source Data Fig. 6 [file 44318_2024_49_MOESM11_ESM.zip › Figure 6/6A/Top Row/L1/DIC.tif]

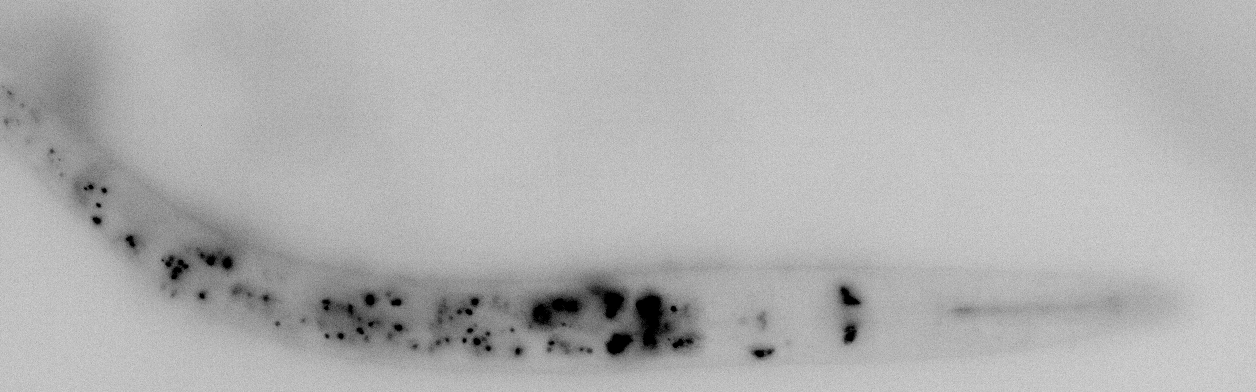

Supplement: Supplementary file 11 — Source Data Fig. 6 [file 44318_2024_49_MOESM11_ESM.zip › Figure 6/6A/Top Row/L1/GFP.tif]

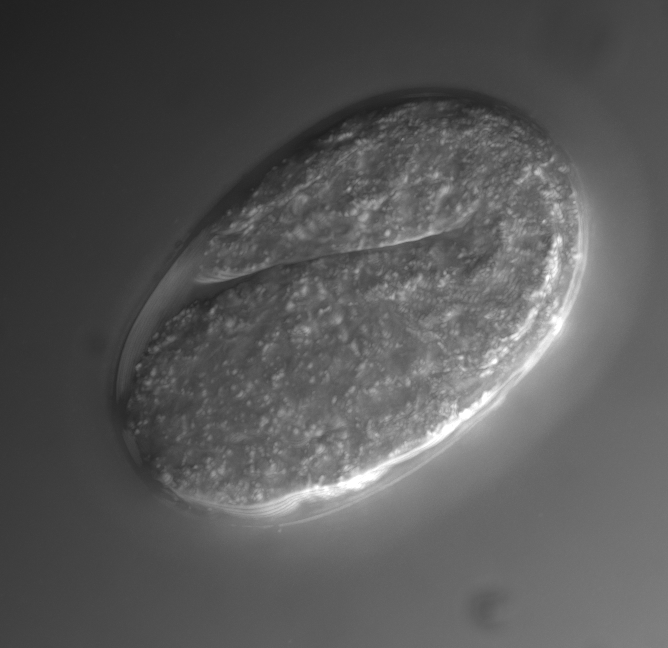

Supplement: Supplementary file 11 — Source Data Fig. 6 [file 44318_2024_49_MOESM11_ESM.zip › Figure 6/6A/Bottom Row/2fold/DIC.tif]

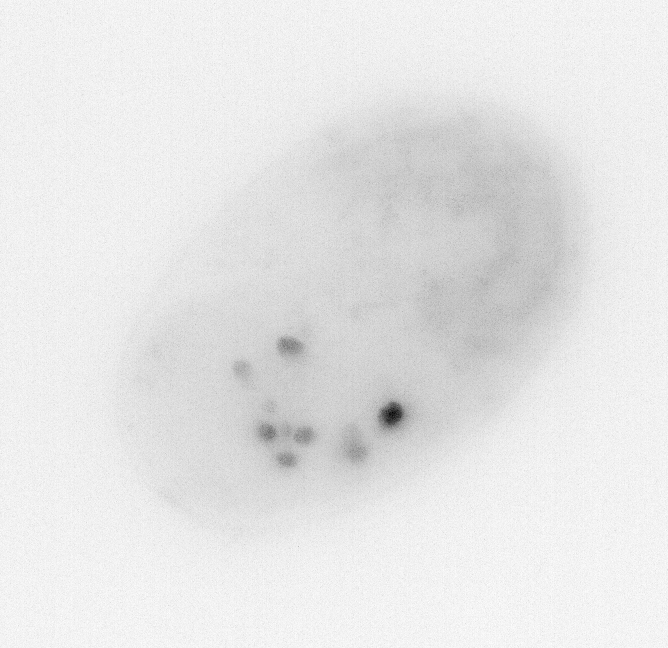

Supplement: Supplementary file 11 — Source Data Fig. 6 [file 44318_2024_49_MOESM11_ESM.zip › Figure 6/6A/Bottom Row/2fold/GFP.tif]

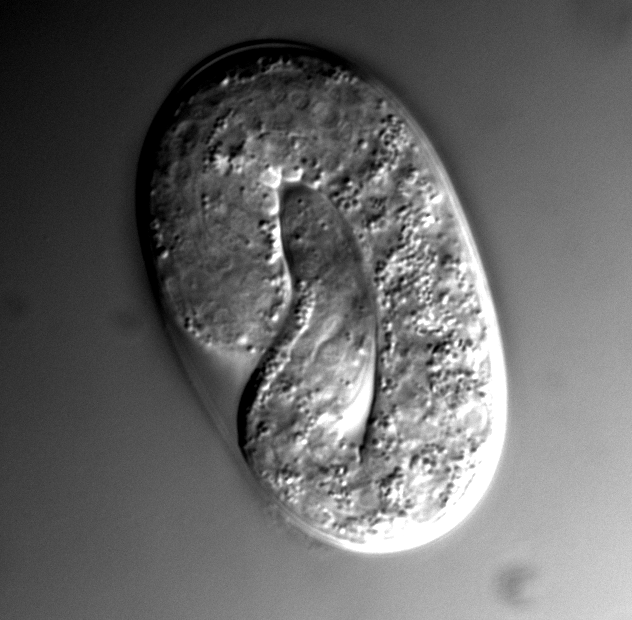

Supplement: Supplementary file 11 — Source Data Fig. 6 [file 44318_2024_49_MOESM11_ESM.zip › Figure 6/6A/Bottom Row/3fold/DIC.tif]

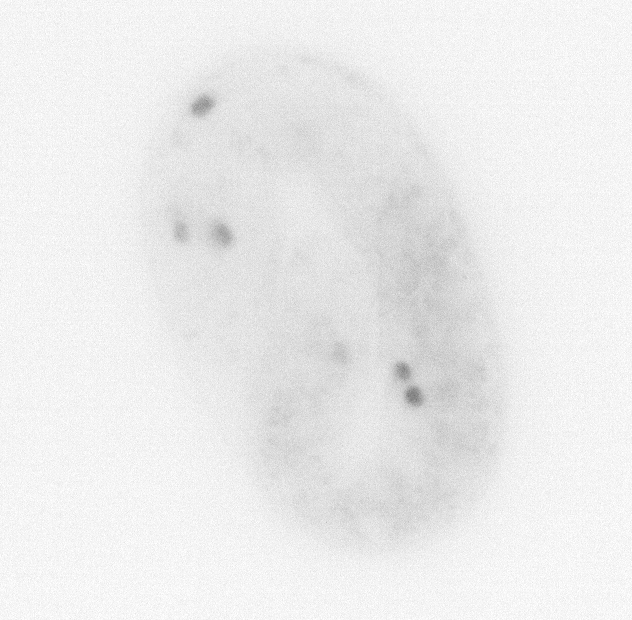

Supplement: Supplementary file 11 — Source Data Fig. 6 [file 44318_2024_49_MOESM11_ESM.zip › Figure 6/6A/Bottom Row/3fold/GFP.tif]

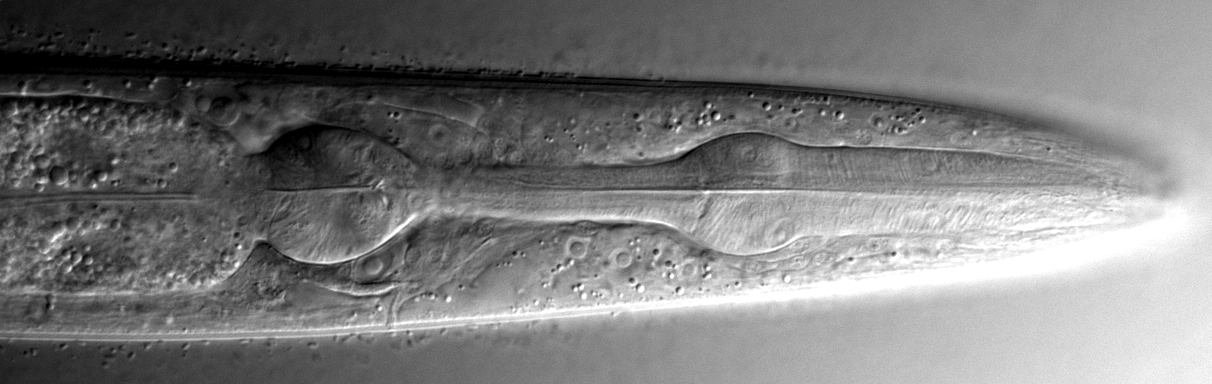

Supplement: Supplementary file 11 — Source Data Fig. 6 [file 44318_2024_49_MOESM11_ESM.zip › Figure 6/6A/Bottom Row/L4/DIC.tif]

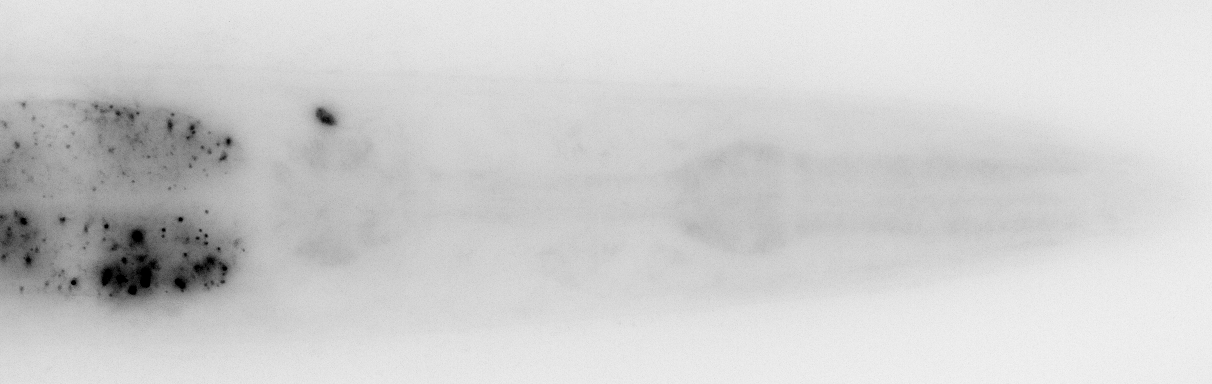

Supplement: Supplementary file 11 — Source Data Fig. 6 [file 44318_2024_49_MOESM11_ESM.zip › Figure 6/6A/Bottom Row/L4/GFP.tif]

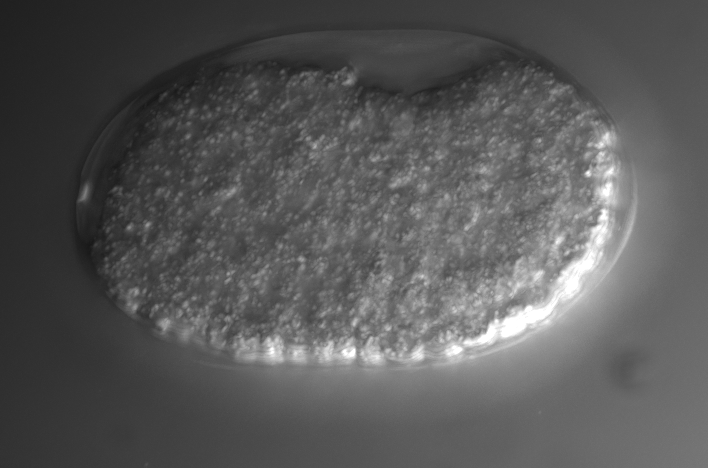

Supplement: Supplementary file 11 — Source Data Fig. 6 [file 44318_2024_49_MOESM11_ESM.zip › Figure 6/6A/Bottom Row/Bean/DIC.tif]

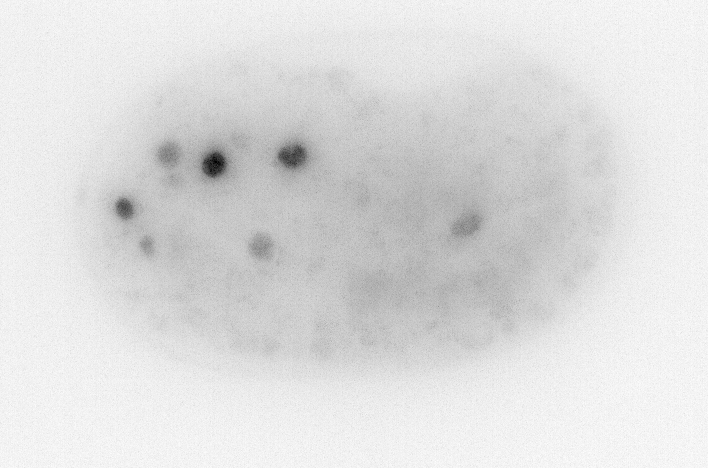

Supplement: Supplementary file 11 — Source Data Fig. 6 [file 44318_2024_49_MOESM11_ESM.zip › Figure 6/6A/Bottom Row/Bean/GFP.tif]

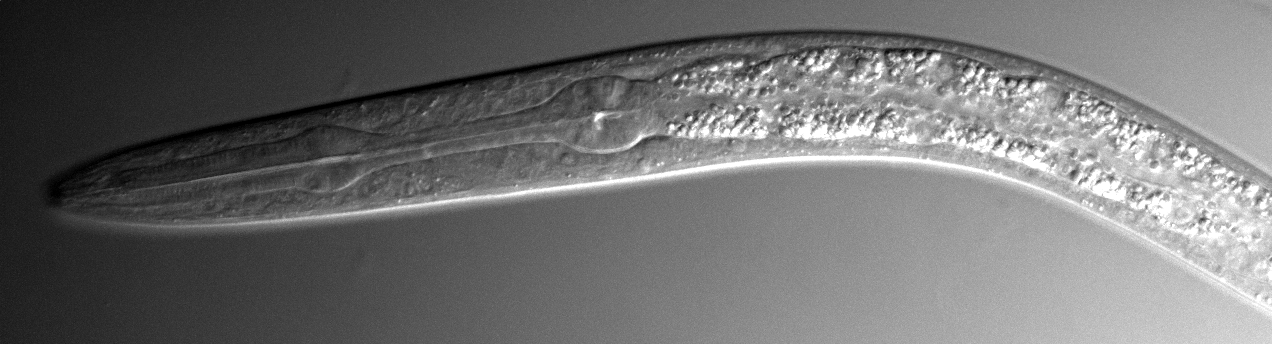

Supplement: Supplementary file 11 — Source Data Fig. 6 [file 44318_2024_49_MOESM11_ESM.zip › Figure 6/6A/Bottom Row/L1/DIC.tif]

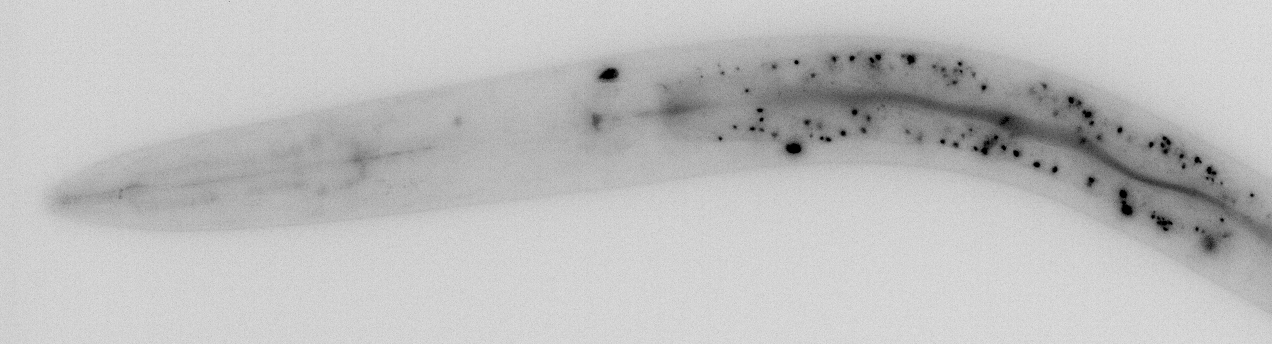

Supplement: Supplementary file 11 — Source Data Fig. 6 [file 44318_2024_49_MOESM11_ESM.zip › Figure 6/6A/Bottom Row/L1/GFP.tif]

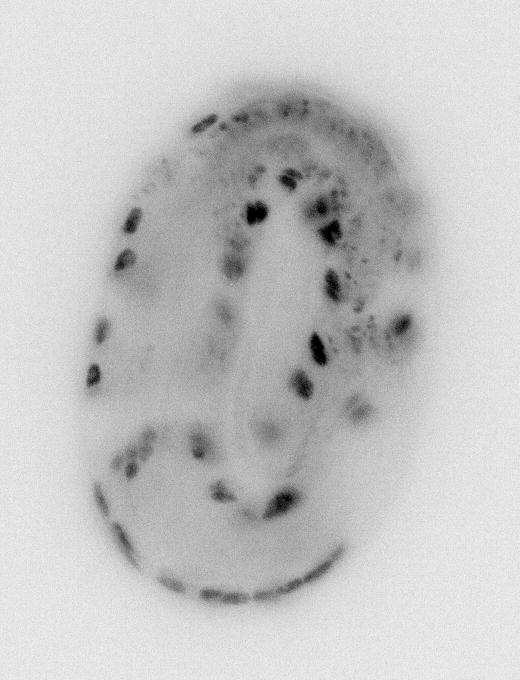

Supplement: Supplementary file 11 — Source Data Fig. 6 [file 44318_2024_49_MOESM11_ESM.zip › Figure 6/6B/Top Row/3fold/GFP.tif]

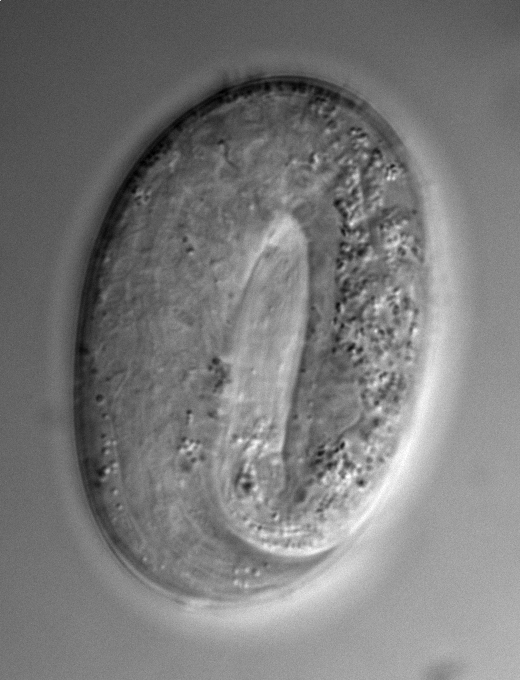

Supplement: Supplementary file 11 — Source Data Fig. 6 [file 44318_2024_49_MOESM11_ESM.zip › Figure 6/6B/Top Row/3fold/DIC copy.tif]

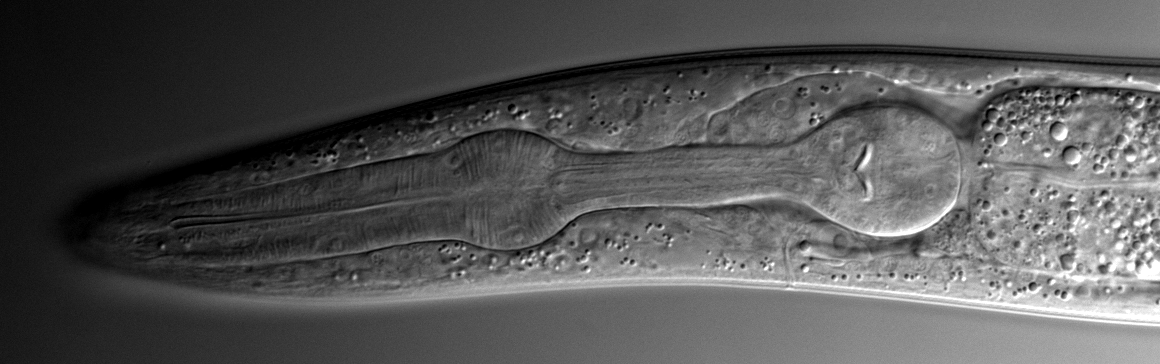

Supplement: Supplementary file 11 — Source Data Fig. 6 [file 44318_2024_49_MOESM11_ESM.zip › Figure 6/6B/Top Row/L4/DIC 1.tif]

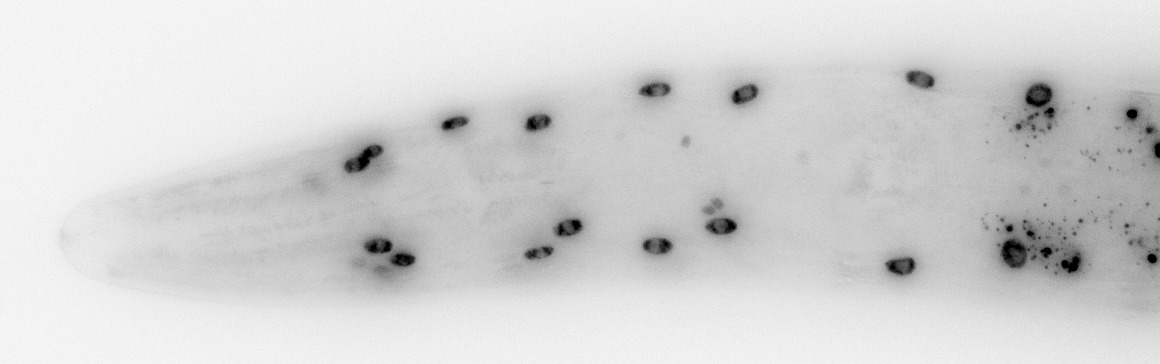

Supplement: Supplementary file 11 — Source Data Fig. 6 [file 44318_2024_49_MOESM11_ESM.zip › Figure 6/6B/Top Row/L4/GFP.tif]

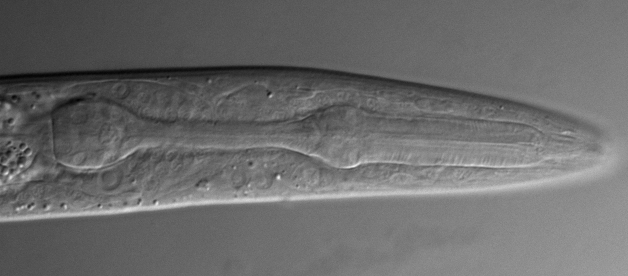

Supplement: Supplementary file 11 — Source Data Fig. 6 [file 44318_2024_49_MOESM11_ESM.zip › Figure 6/6B/Top Row/L1/DIC.tif]

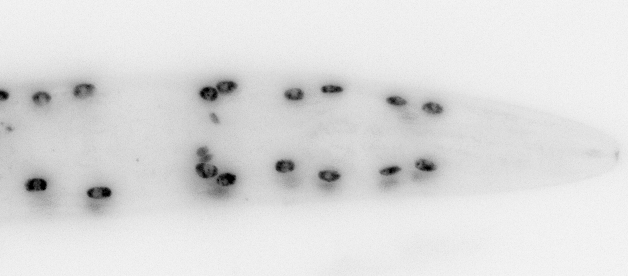

Supplement: Supplementary file 11 — Source Data Fig. 6 [file 44318_2024_49_MOESM11_ESM.zip › Figure 6/6B/Top Row/L1/GFP.tif]

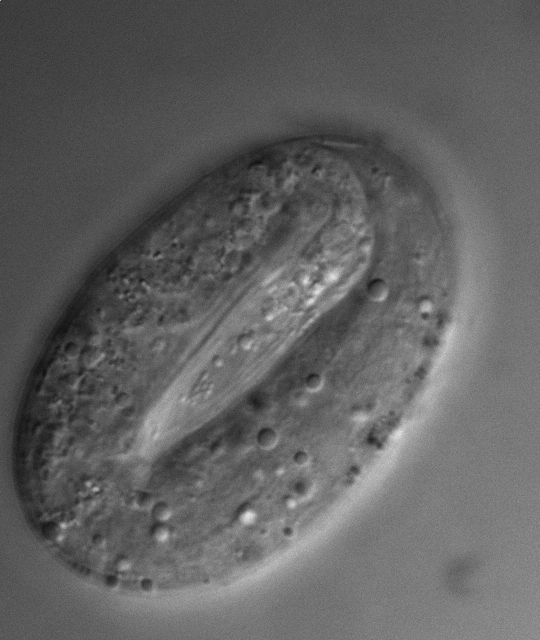

Supplement: Supplementary file 11 — Source Data Fig. 6 [file 44318_2024_49_MOESM11_ESM.zip › Figure 6/6B/Bottom Row/3fold/DIC.tif]

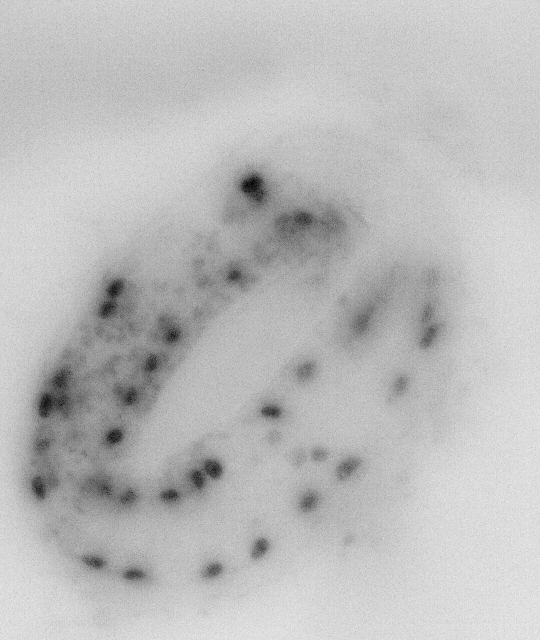

Supplement: Supplementary file 11 — Source Data Fig. 6 [file 44318_2024_49_MOESM11_ESM.zip › Figure 6/6B/Bottom Row/3fold/GFP.tif]

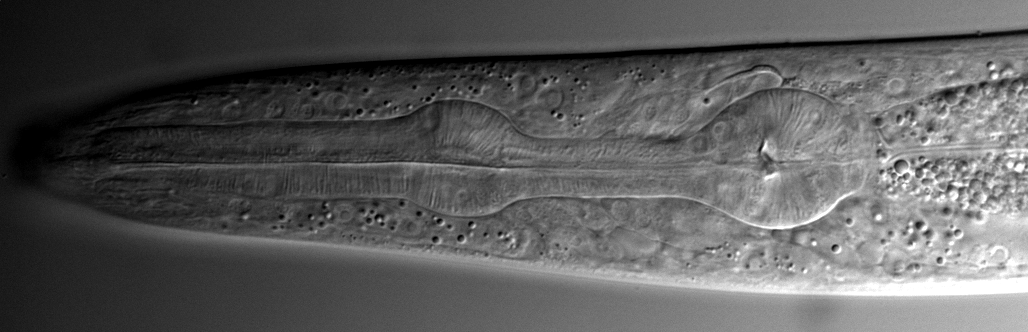

Supplement: Supplementary file 11 — Source Data Fig. 6 [file 44318_2024_49_MOESM11_ESM.zip › Figure 6/6B/Bottom Row/L4/DIC.tif]

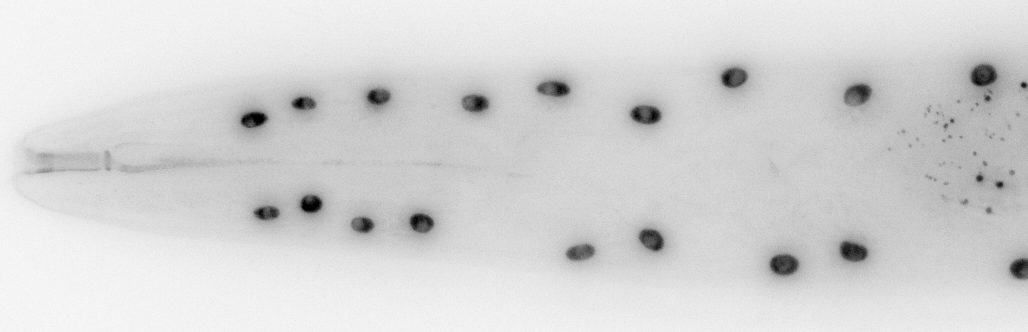

Supplement: Supplementary file 11 — Source Data Fig. 6 [file 44318_2024_49_MOESM11_ESM.zip › Figure 6/6B/Bottom Row/L4/GFP.tif]

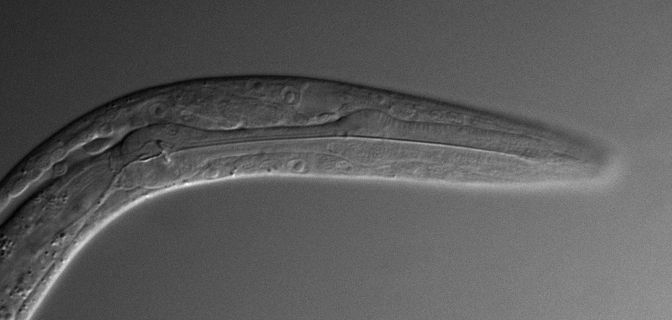

Supplement: Supplementary file 11 — Source Data Fig. 6 [file 44318_2024_49_MOESM11_ESM.zip › Figure 6/6B/Bottom Row/L1/DIC.tif]

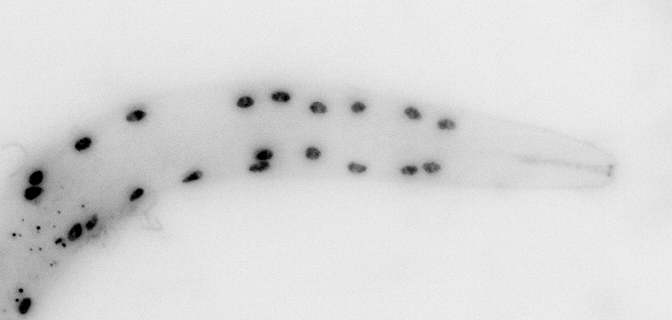

Supplement: Supplementary file 11 — Source Data Fig. 6 [file 44318_2024_49_MOESM11_ESM.zip › Figure 6/6B/Bottom Row/L1/GFP.tif]

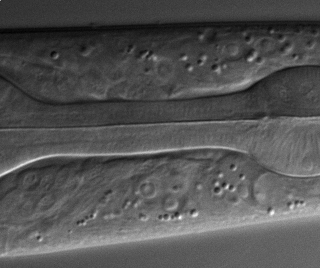

Supplement: Supplementary file 11 — Source Data Fig. 6 [file 44318_2024_49_MOESM11_ESM.zip › Figure 6/6E/Top Row/L4/DIC.tif]

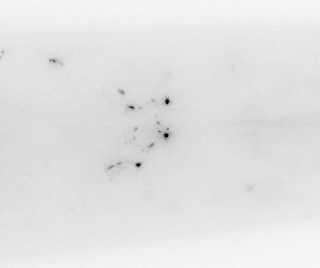

Supplement: Supplementary file 11 — Source Data Fig. 6 [file 44318_2024_49_MOESM11_ESM.zip › Figure 6/6E/Top Row/L4/GFP.tif]

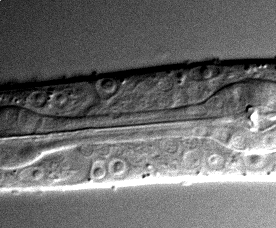

Supplement: Supplementary file 11 — Source Data Fig. 6 [file 44318_2024_49_MOESM11_ESM.zip › Figure 6/6E/Top Row/L1/DIC.tif]

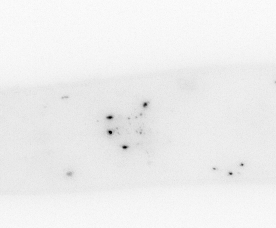

Supplement: Supplementary file 11 — Source Data Fig. 6 [file 44318_2024_49_MOESM11_ESM.zip › Figure 6/6E/Top Row/L1/GFP.tif]

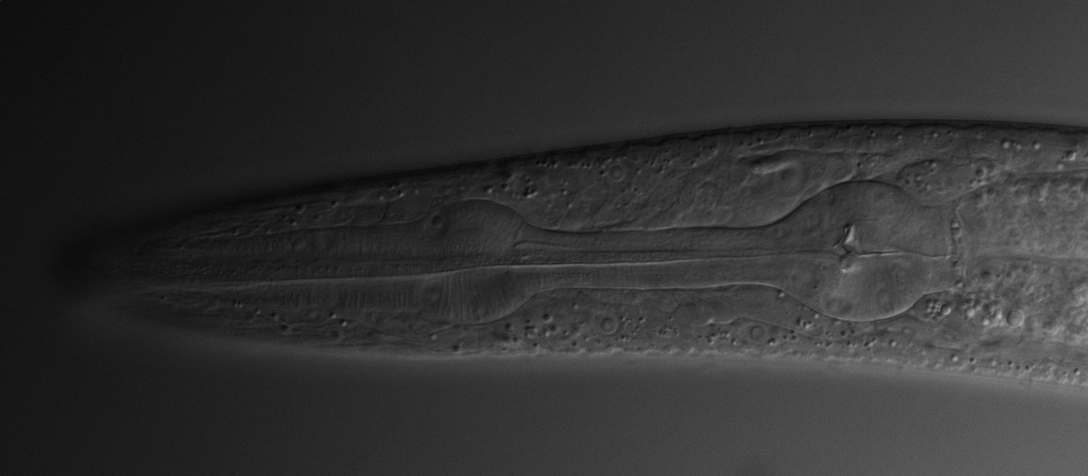

Supplement: Supplementary file 11 — Source Data Fig. 6 [file 44318_2024_49_MOESM11_ESM.zip › Figure 6/6E/Bottom Row/L4/DIC.tif]

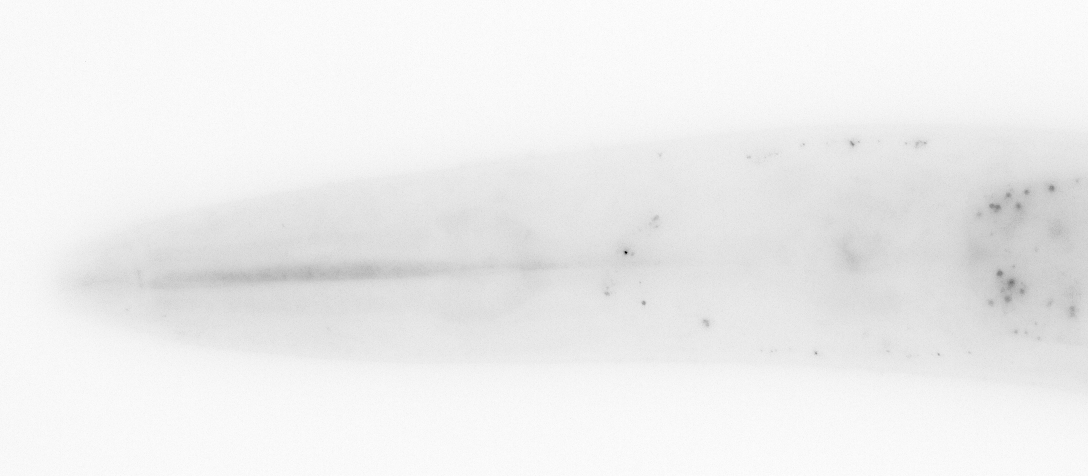

Supplement: Supplementary file 11 — Source Data Fig. 6 [file 44318_2024_49_MOESM11_ESM.zip › Figure 6/6E/Bottom Row/L4/GFP.tif]

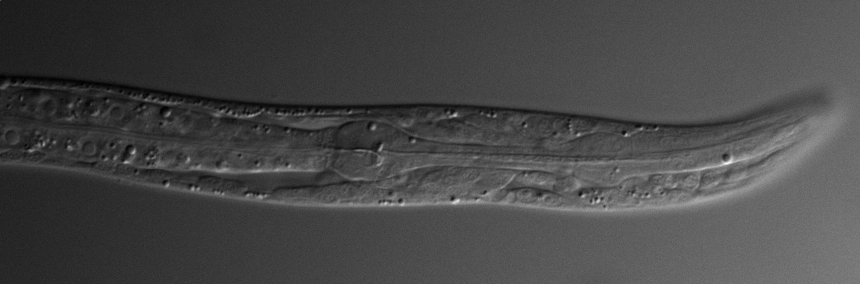

Supplement: Supplementary file 11 — Source Data Fig. 6 [file 44318_2024_49_MOESM11_ESM.zip › Figure 6/6E/Bottom Row/L1/DIC.tif]

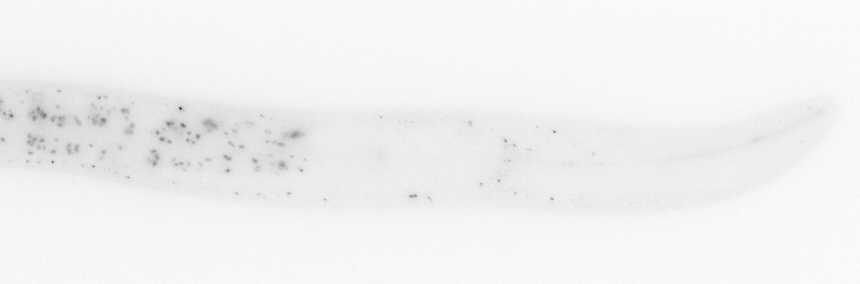

Supplement: Supplementary file 11 — Source Data Fig. 6 [file 44318_2024_49_MOESM11_ESM.zip › Figure 6/6E/Bottom Row/L1/GFP.tif]

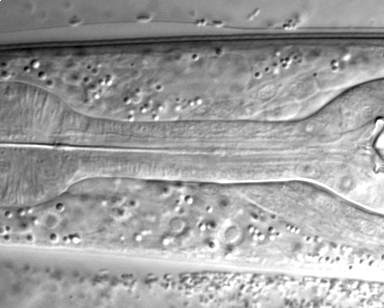

Supplement: Supplementary file 11 — Source Data Fig. 6 [file 44318_2024_49_MOESM11_ESM.zip › Figure 6/6D/Top Row/L4/DIC.tif]

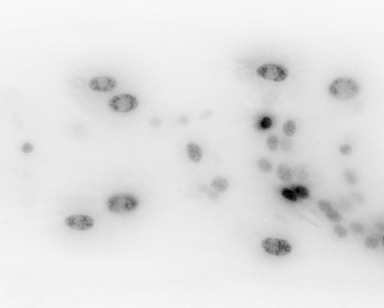

Supplement: Supplementary file 11 — Source Data Fig. 6 [file 44318_2024_49_MOESM11_ESM.zip › Figure 6/6D/Top Row/L4/GFP.tif]

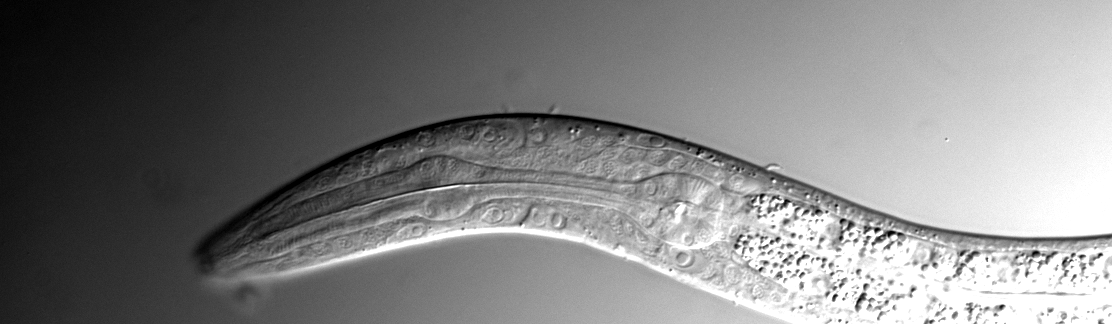

Supplement: Supplementary file 11 — Source Data Fig. 6 [file 44318_2024_49_MOESM11_ESM.zip › Figure 6/6D/Top Row/L1/DIC.tif]

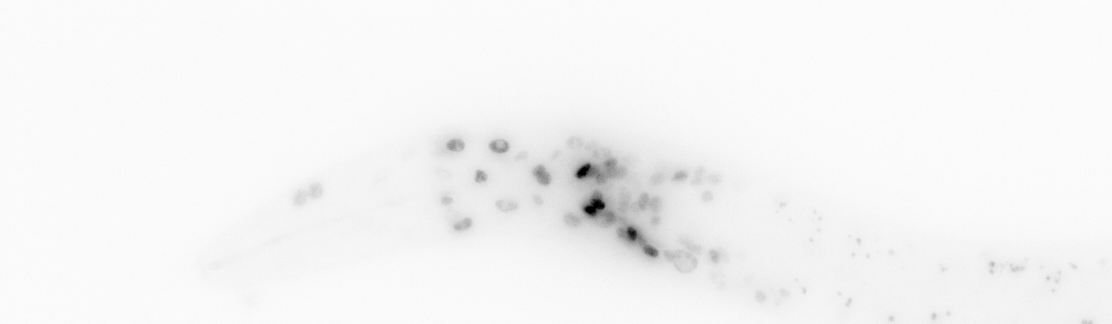

Supplement: Supplementary file 11 — Source Data Fig. 6 [file 44318_2024_49_MOESM11_ESM.zip › Figure 6/6D/Top Row/L1/GFP.tif]

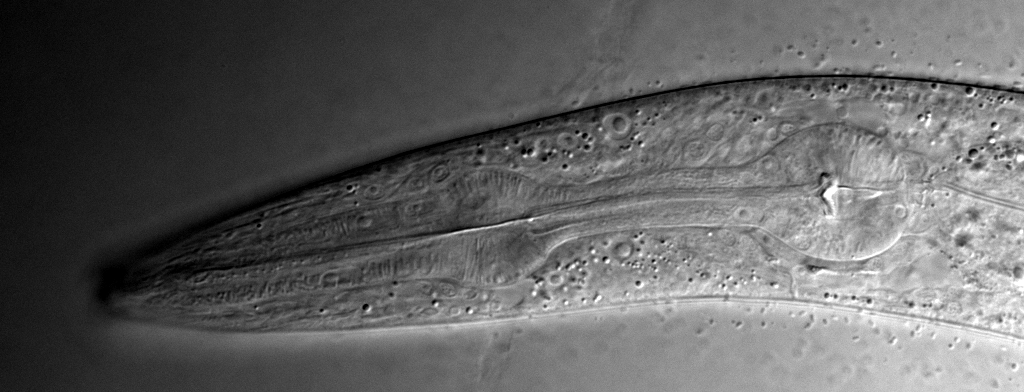

Supplement: Supplementary file 11 — Source Data Fig. 6 [file 44318_2024_49_MOESM11_ESM.zip › Figure 6/6D/Bottom Row/L4/DIC.tif]

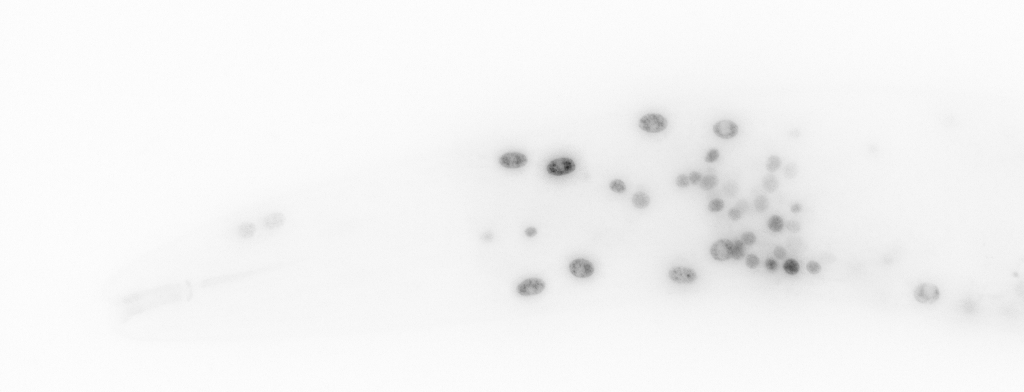

Supplement: Supplementary file 11 — Source Data Fig. 6 [file 44318_2024_49_MOESM11_ESM.zip › Figure 6/6D/Bottom Row/L4/GFP.tif]

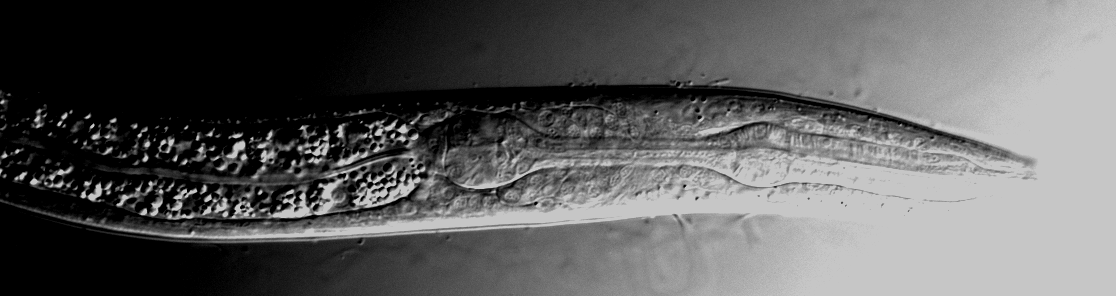

Supplement: Supplementary file 11 — Source Data Fig. 6 [file 44318_2024_49_MOESM11_ESM.zip › Figure 6/6D/Bottom Row/L1/DIC.tif]

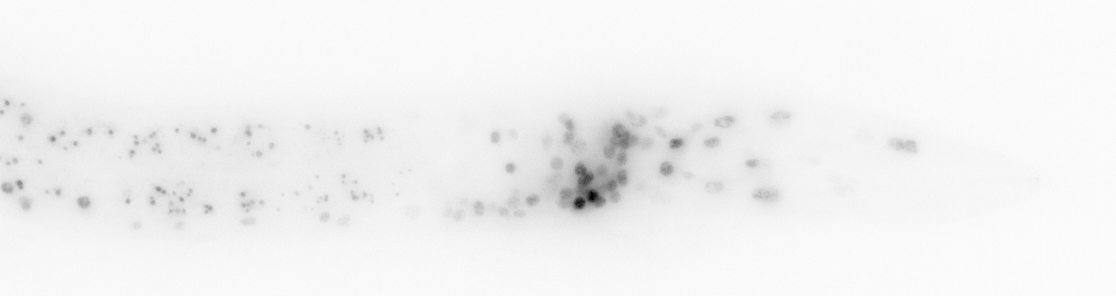

Supplement: Supplementary file 11 — Source Data Fig. 6 [file 44318_2024_49_MOESM11_ESM.zip › Figure 6/6D/Bottom Row/L1/GFP.tif]

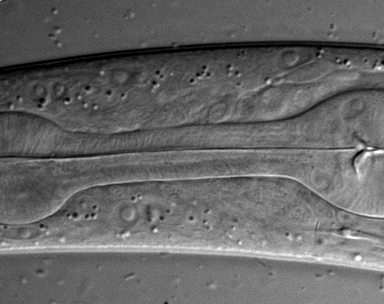

Supplement: Supplementary file 11 — Source Data Fig. 6 [file 44318_2024_49_MOESM11_ESM.zip › Figure 6/6C/Top Row/L4/DIC.tif]

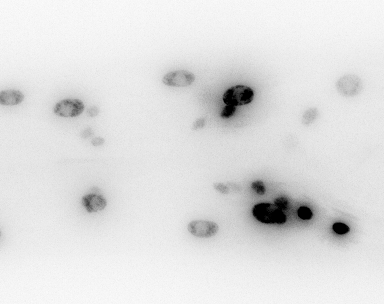

Supplement: Supplementary file 11 — Source Data Fig. 6 [file 44318_2024_49_MOESM11_ESM.zip › Figure 6/6C/Top Row/L4/GFP.tif]

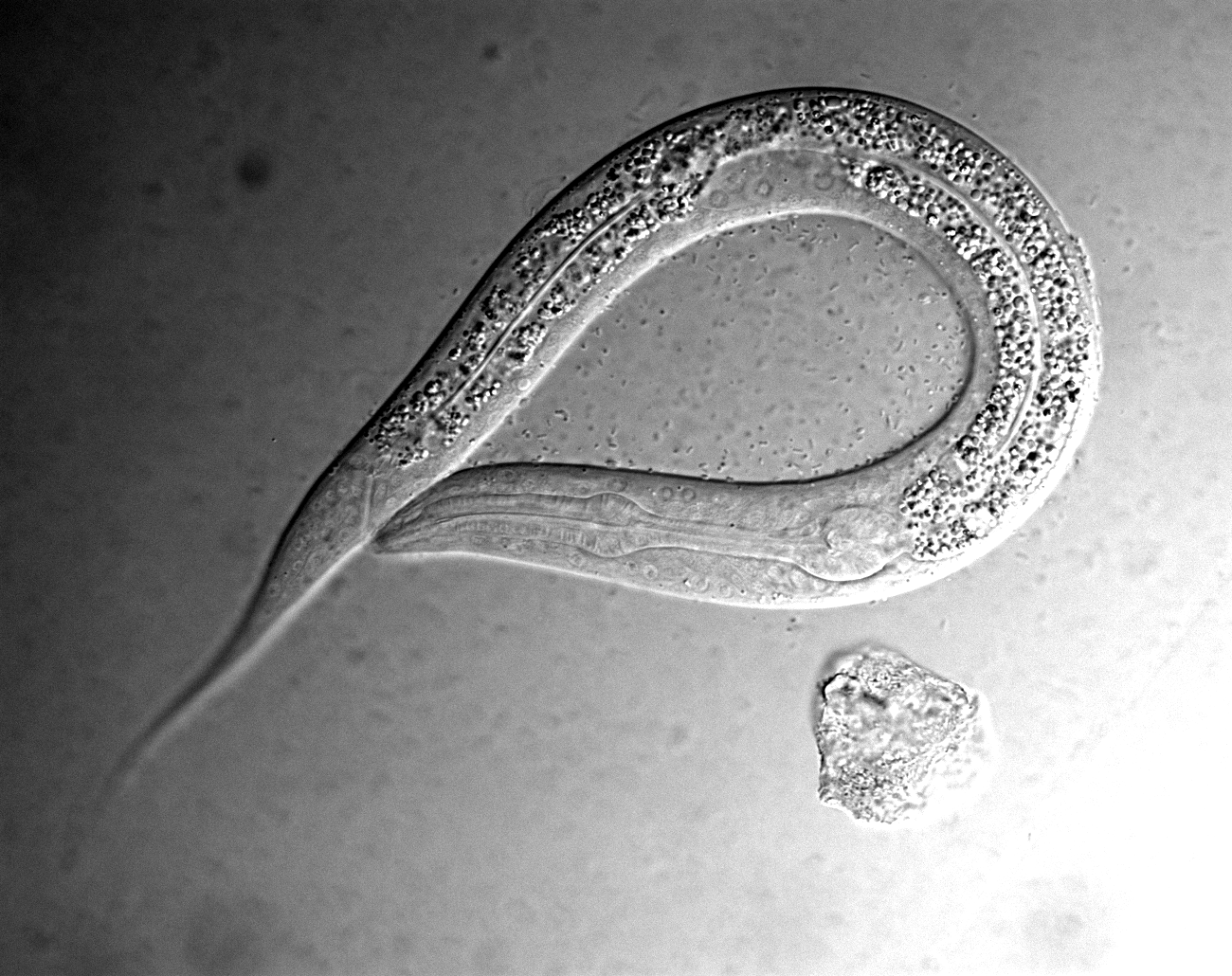

Supplement: Supplementary file 11 — Source Data Fig. 6 [file 44318_2024_49_MOESM11_ESM.zip › Figure 6/6C/Top Row/L1/DIC.tif]

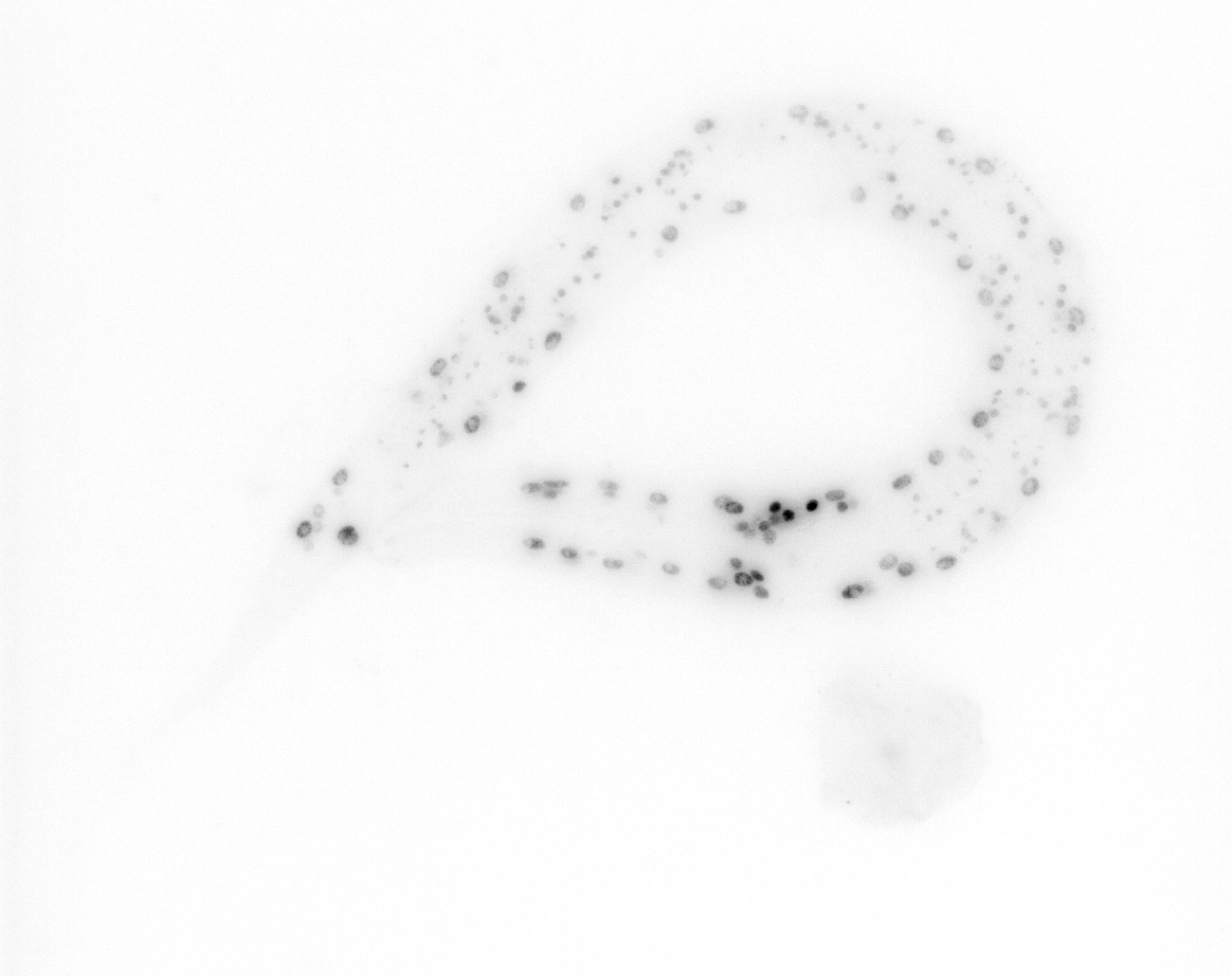

Supplement: Supplementary file 11 — Source Data Fig. 6 [file 44318_2024_49_MOESM11_ESM.zip › Figure 6/6C/Top Row/L1/GFP.tif]

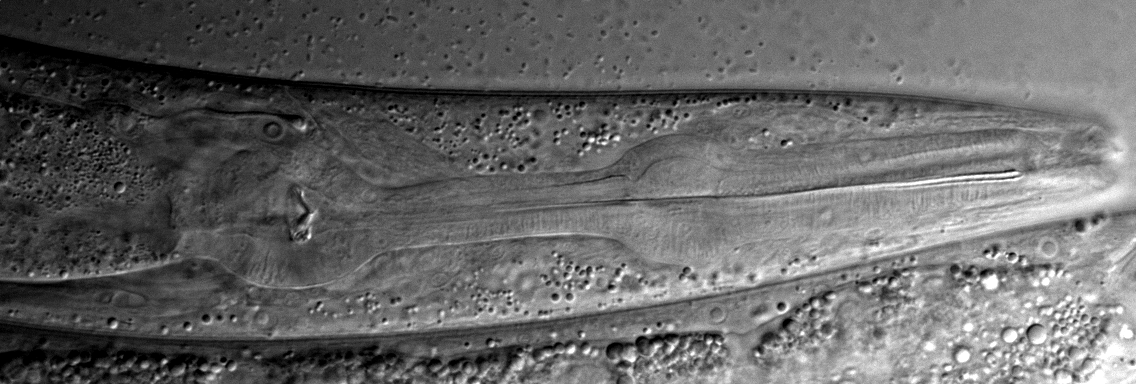

Supplement: Supplementary file 11 — Source Data Fig. 6 [file 44318_2024_49_MOESM11_ESM.zip › Figure 6/6C/Bottom Row/L4/DIC.tif]

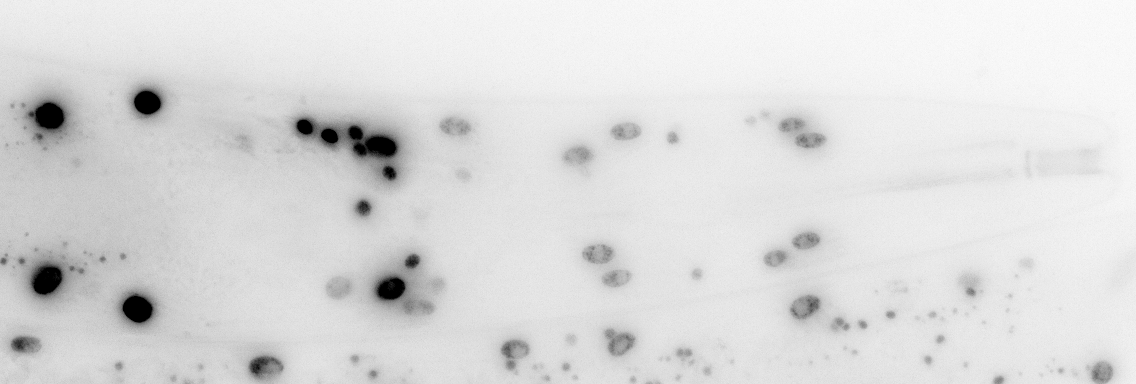

Supplement: Supplementary file 11 — Source Data Fig. 6 [file 44318_2024_49_MOESM11_ESM.zip › Figure 6/6C/Bottom Row/L4/GFP.tif]

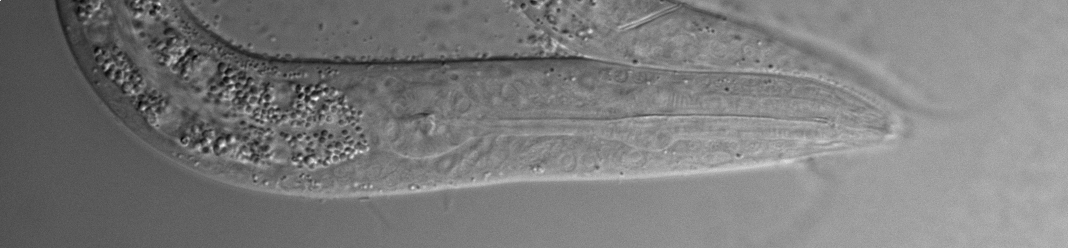

Supplement: Supplementary file 11 — Source Data Fig. 6 [file 44318_2024_49_MOESM11_ESM.zip › Figure 6/6C/Bottom Row/L1/DIC.tif]

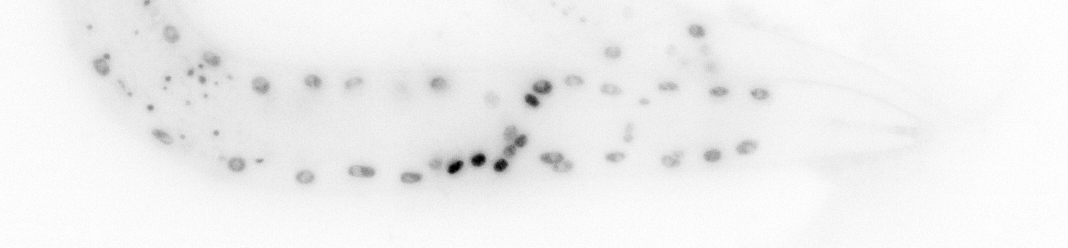

Supplement: Supplementary file 11 — Source Data Fig. 6 [file 44318_2024_49_MOESM11_ESM.zip › Figure 6/6C/Bottom Row/L1/GFP.tif]

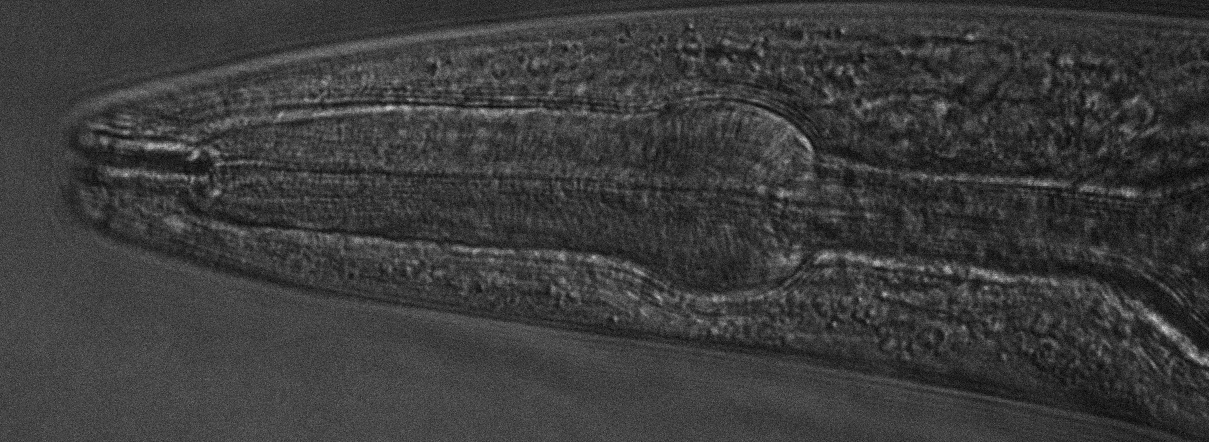

Supplement: Supplementary file 12 — Source Data Fig. 7 [file 44318_2024_49_MOESM12_ESM.zip › Figure 7/7C/left (wild type)/DIC.tif]

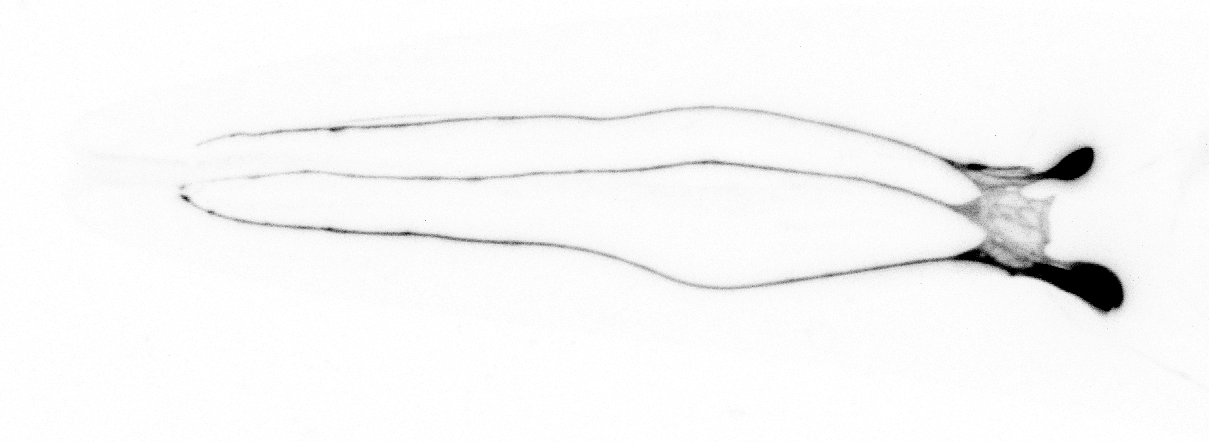

Supplement: Supplementary file 12 — Source Data Fig. 7 [file 44318_2024_49_MOESM12_ESM.zip › Figure 7/7C/left (wild type)/GFP.tif]

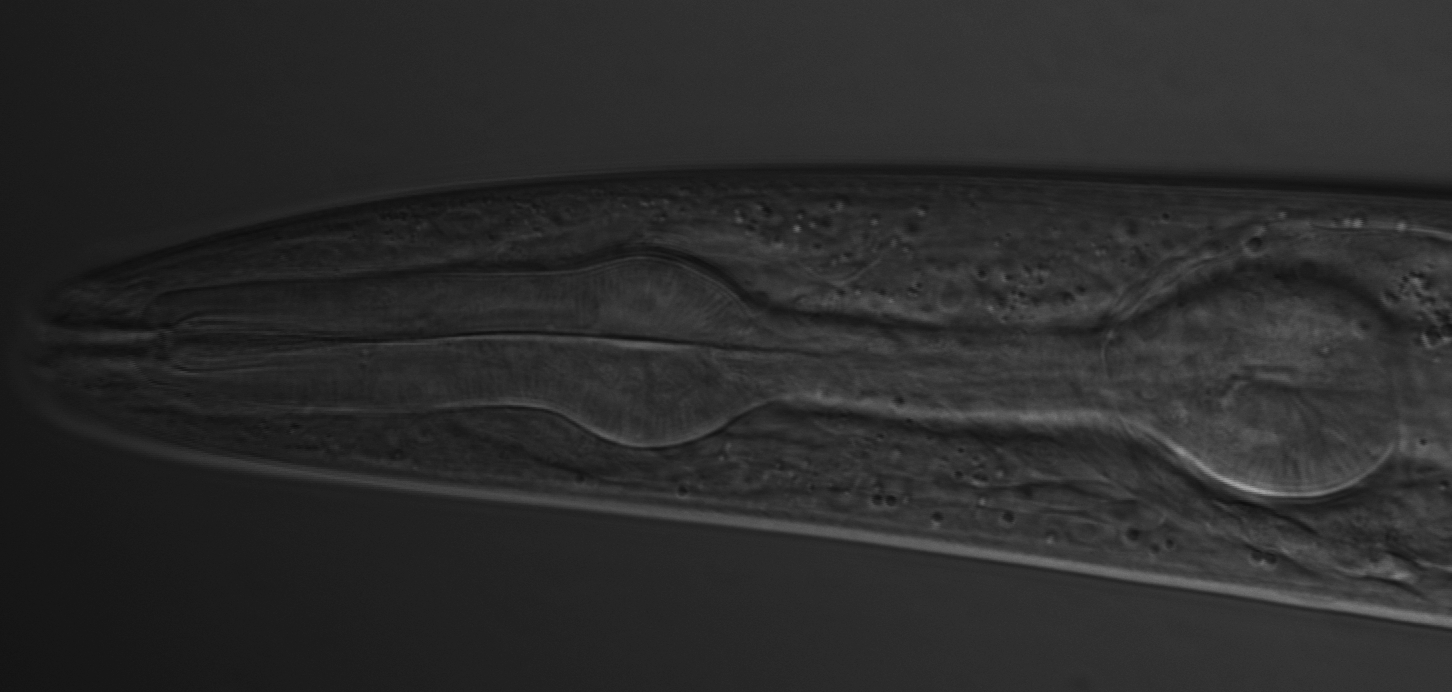

Supplement: Supplementary file 12 — Source Data Fig. 7 [file 44318_2024_49_MOESM12_ESM.zip › Figure 7/7C/right [unc-30(e191)]/DIC.tif]

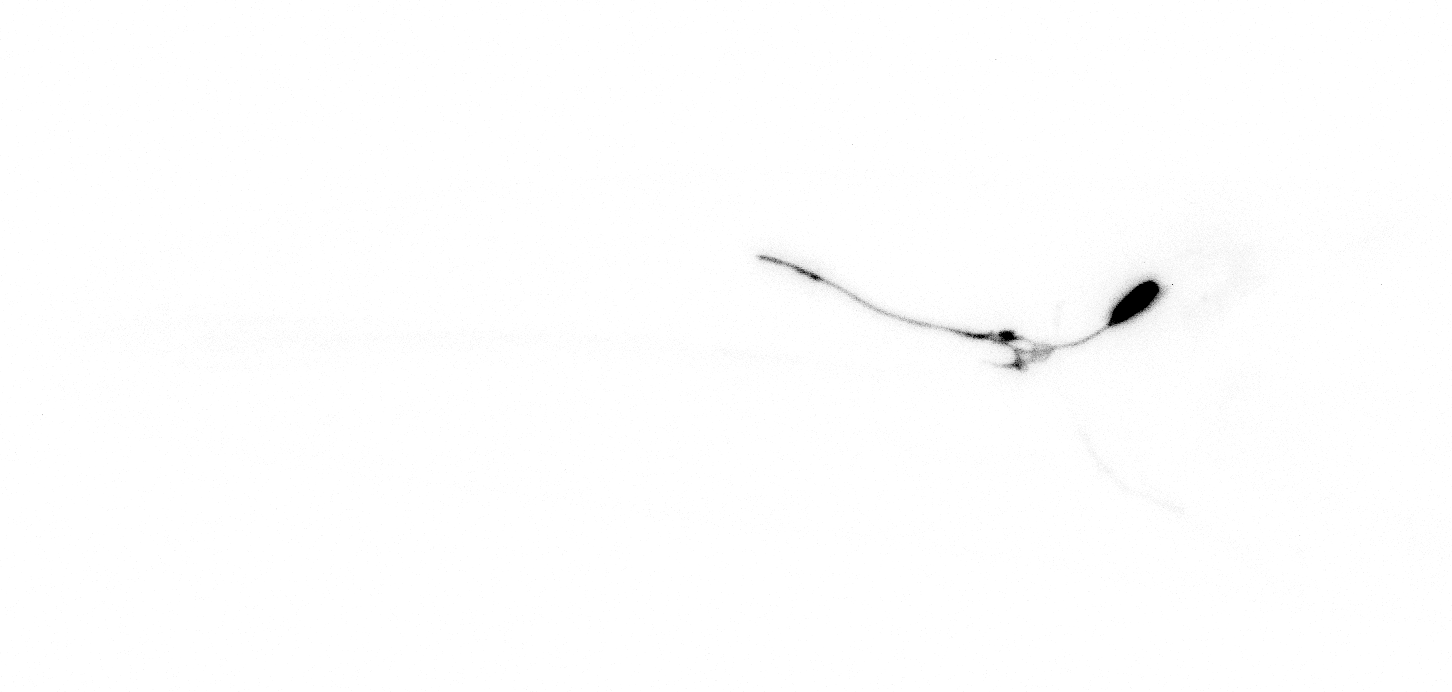

Supplement: Supplementary file 12 — Source Data Fig. 7 [file 44318_2024_49_MOESM12_ESM.zip › Figure 7/7C/right [unc-30(e191)]/GFP.tif]

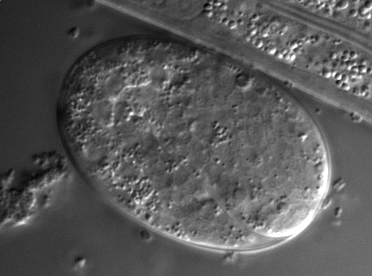

Supplement: Supplementary file 12 — Source Data Fig. 7 [file 44318_2024_49_MOESM12_ESM.zip › Figure 7/7B/2fold/DIC.tif]

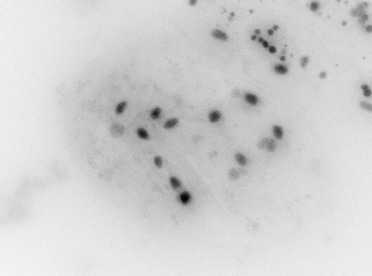

Supplement: Supplementary file 12 — Source Data Fig. 7 [file 44318_2024_49_MOESM12_ESM.zip › Figure 7/7B/2fold/GFP.tif]

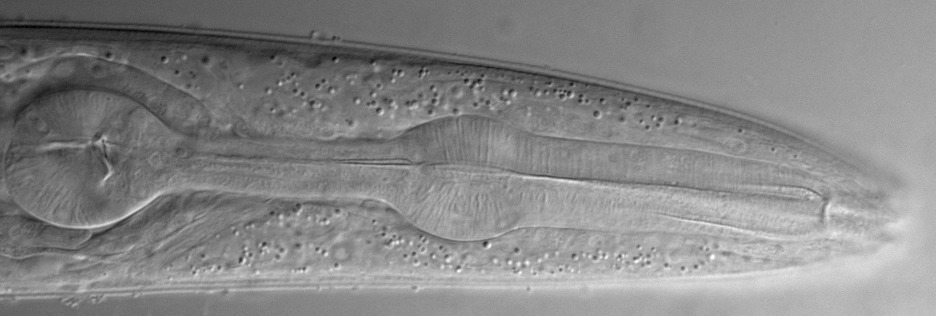

Supplement: Supplementary file 12 — Source Data Fig. 7 [file 44318_2024_49_MOESM12_ESM.zip › Figure 7/7B/young adult/DIC.tif]

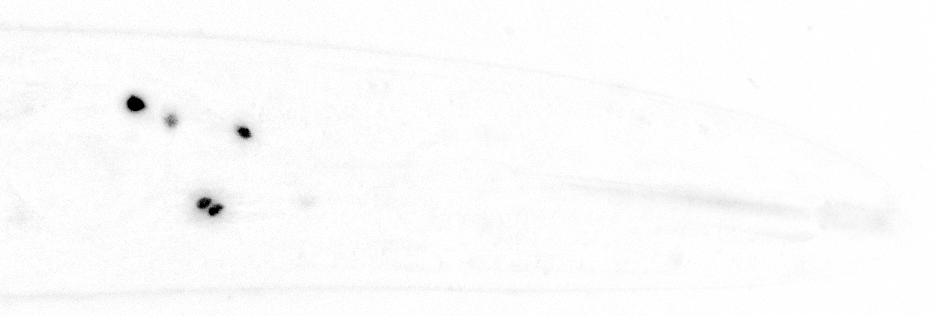

Supplement: Supplementary file 12 — Source Data Fig. 7 [file 44318_2024_49_MOESM12_ESM.zip › Figure 7/7B/young adult/GFP.tif]

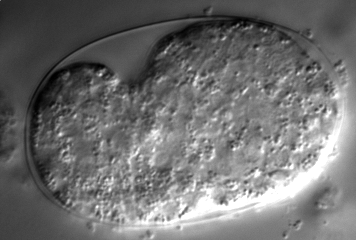

Supplement: Supplementary file 12 — Source Data Fig. 7 [file 44318_2024_49_MOESM12_ESM.zip › Figure 7/7B/bean/DIC.tif]

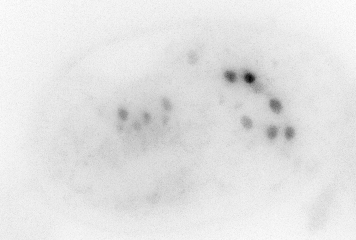

Supplement: Supplementary file 12 — Source Data Fig. 7 [file 44318_2024_49_MOESM12_ESM.zip › Figure 7/7B/bean/GFP.tif]

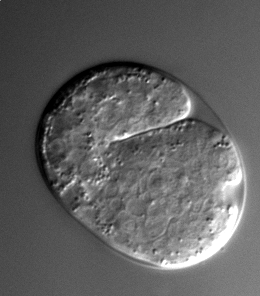

Supplement: Supplementary file 12 — Source Data Fig. 7 [file 44318_2024_49_MOESM12_ESM.zip › Figure 7/7B/comma/DIC.tif]

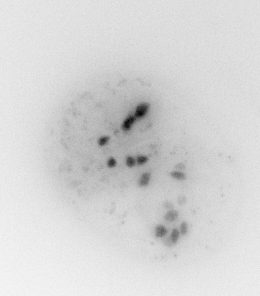

Supplement: Supplementary file 12 — Source Data Fig. 7 [file 44318_2024_49_MOESM12_ESM.zip › Figure 7/7B/comma/GFP.tif]

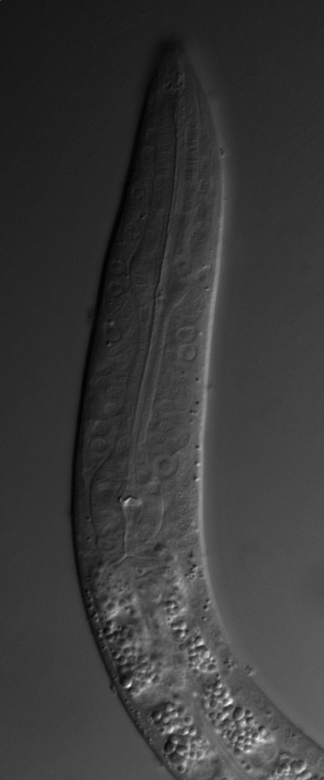

Supplement: Supplementary file 12 — Source Data Fig. 7 [file 44318_2024_49_MOESM12_ESM.zip › Figure 7/7B/L1/DIC.tif]

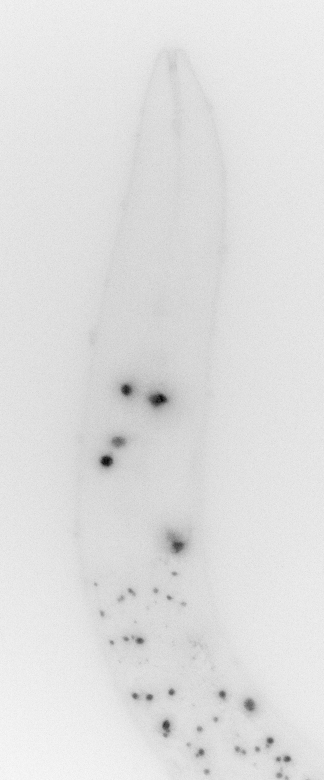

Supplement: Supplementary file 12 — Source Data Fig. 7 [file 44318_2024_49_MOESM12_ESM.zip › Figure 7/7B/L1/GFP.tif]

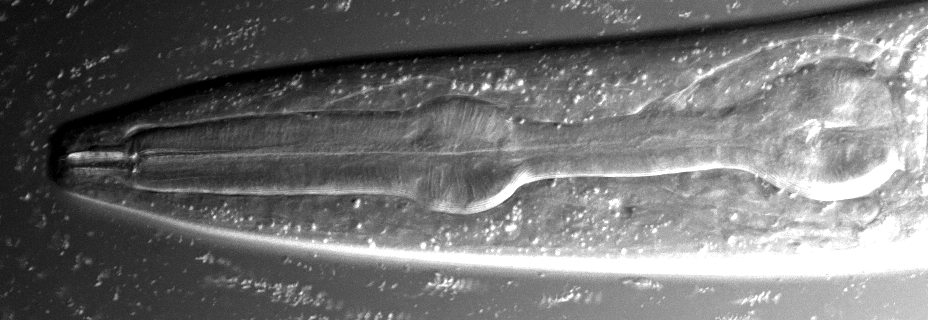

Supplement: Supplementary file 12 — Source Data Fig. 7 [file 44318_2024_49_MOESM12_ESM.zip › Figure 7/7F/1st let-381 motif mutation/DIC.tif]

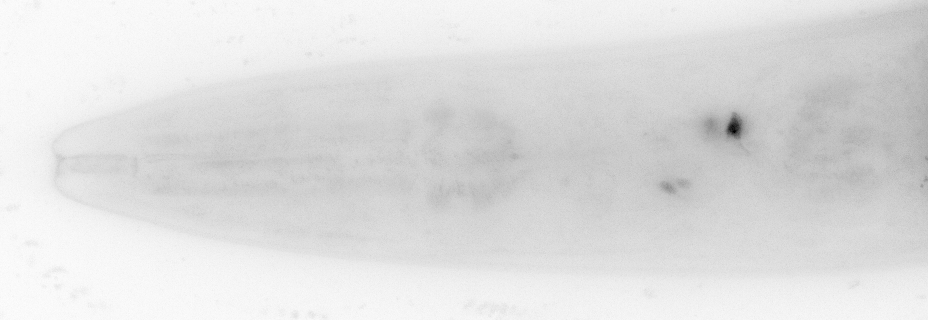

Supplement: Supplementary file 12 — Source Data Fig. 7 [file 44318_2024_49_MOESM12_ESM.zip › Figure 7/7F/1st let-381 motif mutation/GFP.tif]

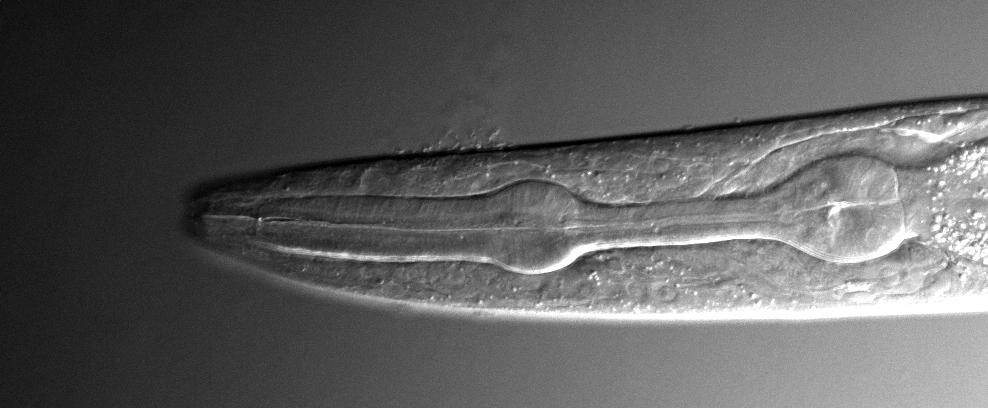

Supplement: Supplementary file 12 — Source Data Fig. 7 [file 44318_2024_49_MOESM12_ESM.zip › Figure 7/7F/wild type/DIC.tif]

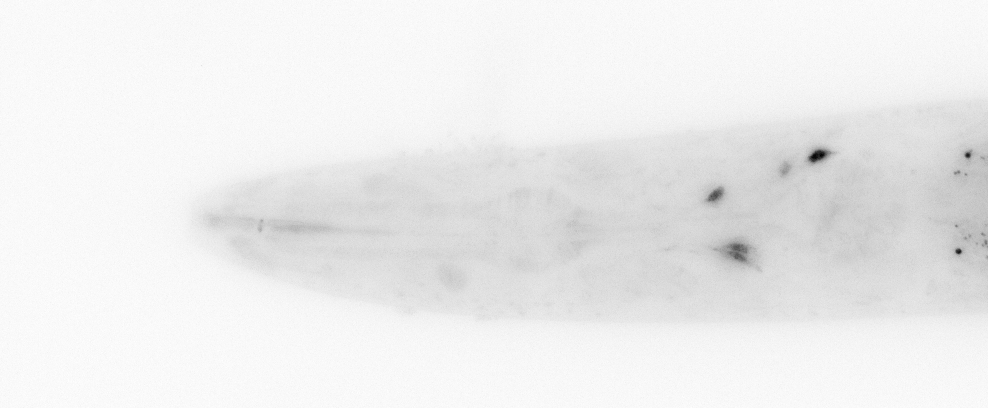

Supplement: Supplementary file 12 — Source Data Fig. 7 [file 44318_2024_49_MOESM12_ESM.zip › Figure 7/7F/wild type/GFP.tif]

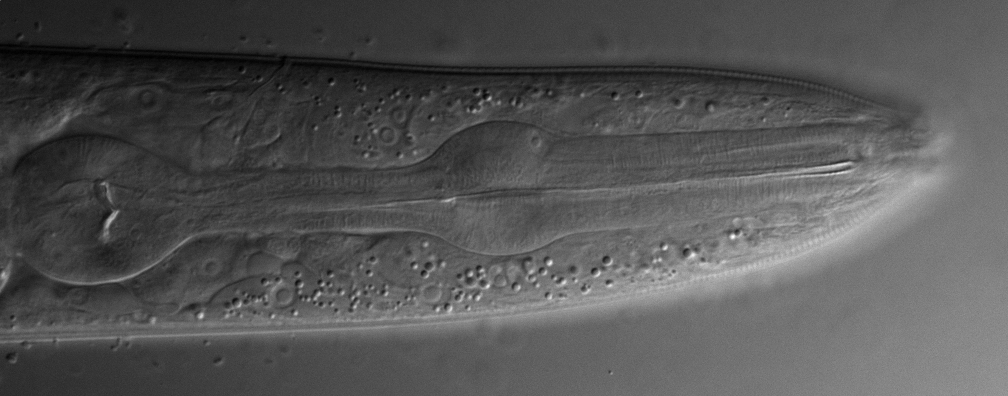

Supplement: Supplementary file 12 — Source Data Fig. 7 [file 44318_2024_49_MOESM12_ESM.zip › Figure 7/7F/169bp deletion of all 3 motifs unc-30(ns998)/DIC.tif]

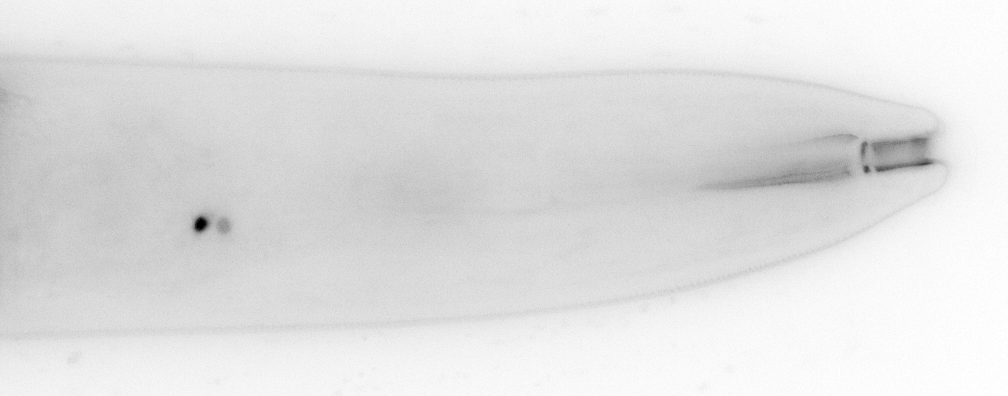

Supplement: Supplementary file 12 — Source Data Fig. 7 [file 44318_2024_49_MOESM12_ESM.zip › Figure 7/7F/169bp deletion of all 3 motifs unc-30(ns998)/GFP.tif]

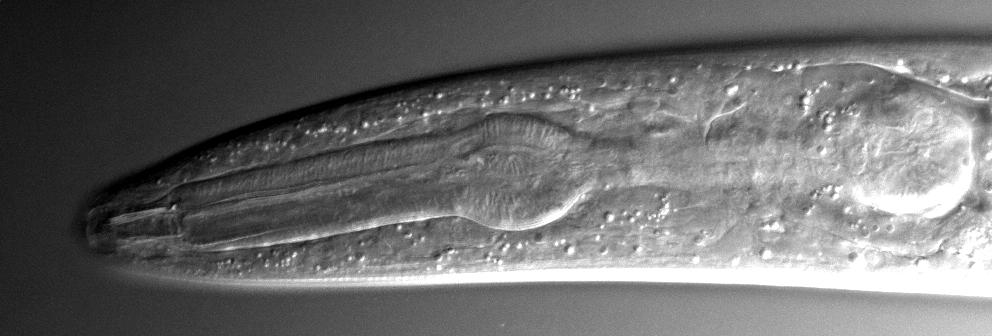

Supplement: Supplementary file 12 — Source Data Fig. 7 [file 44318_2024_49_MOESM12_ESM.zip › Figure 7/7F/3rd let-381 motif mutation/DIC.tif]
