# Supplementary figures and images for: LET-381/FoxF and its target UNC-30/Pitx2 specify and maintain the molecular identity of C. elegans mesodermal glia that regulate motor behavior (part 3 of 3)
Source: EMBO J. 2024 Feb 15;43(6):4. doi: 10.1038/s44318-024-00049-w (PMC10943081; doi:10.1038/s44318-024-00049-w)

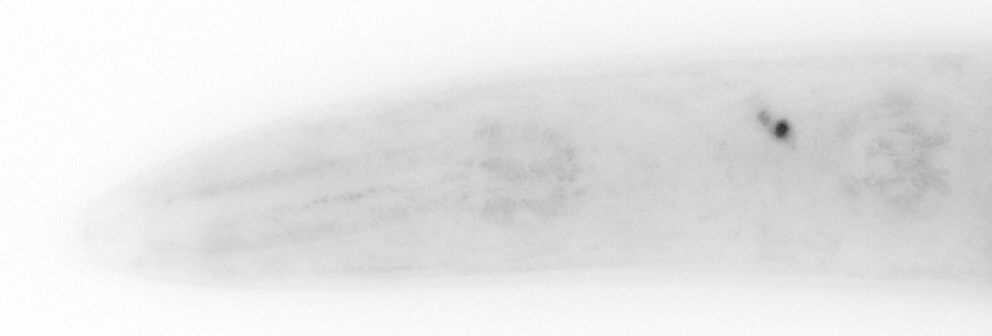

Supplement: Supplementary file 12 — Source Data Fig. 7 [file 44318_2024_49_MOESM12_ESM.zip › Figure 7/7F/3rd let-381 motif mutation/GFP.tif]

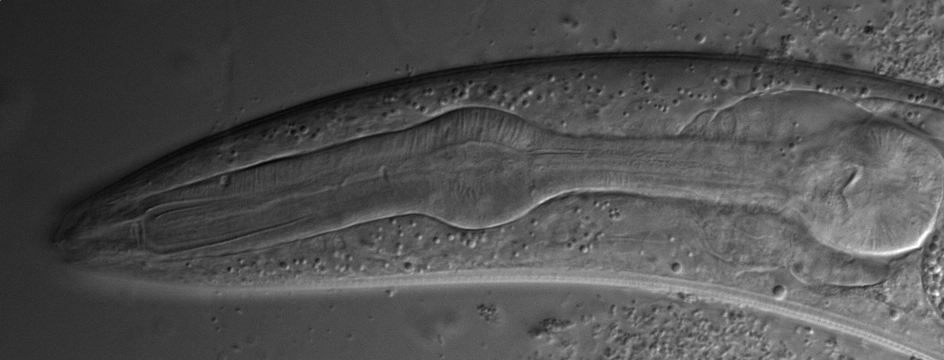

Supplement: Supplementary file 12 — Source Data Fig. 7 [file 44318_2024_49_MOESM12_ESM.zip › Figure 7/7F/1st + 2nd let-381 motif mutation/DIC.tif]

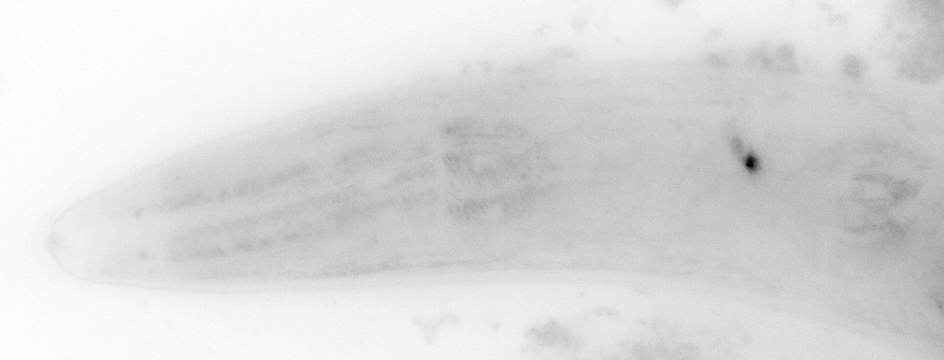

Supplement: Supplementary file 12 — Source Data Fig. 7 [file 44318_2024_49_MOESM12_ESM.zip › Figure 7/7F/1st + 2nd let-381 motif mutation/GFP.tif]

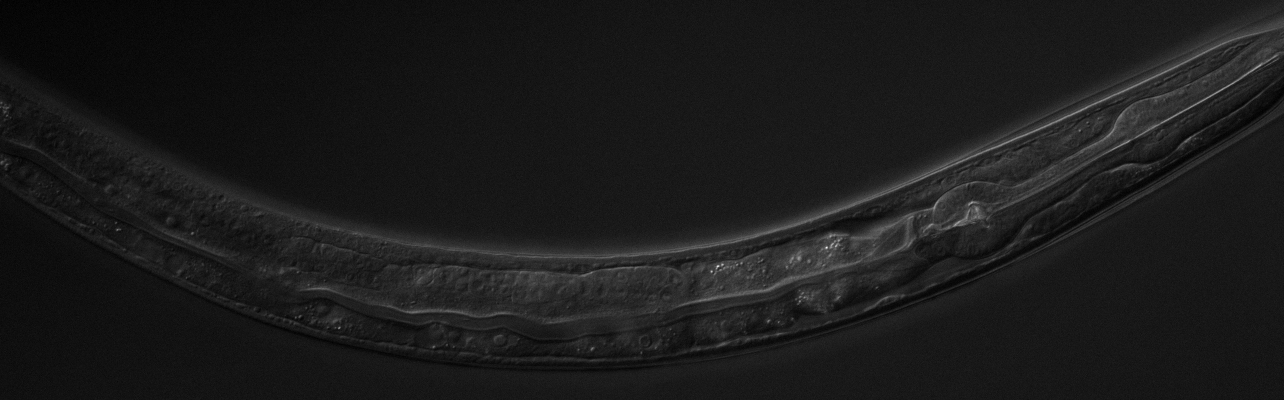

Supplement: Supplementary file 13 — Source Data Fig. 8 [file 44318_2024_49_MOESM13_ESM.zip › Figure 8/8G/Top/DIC.tif]

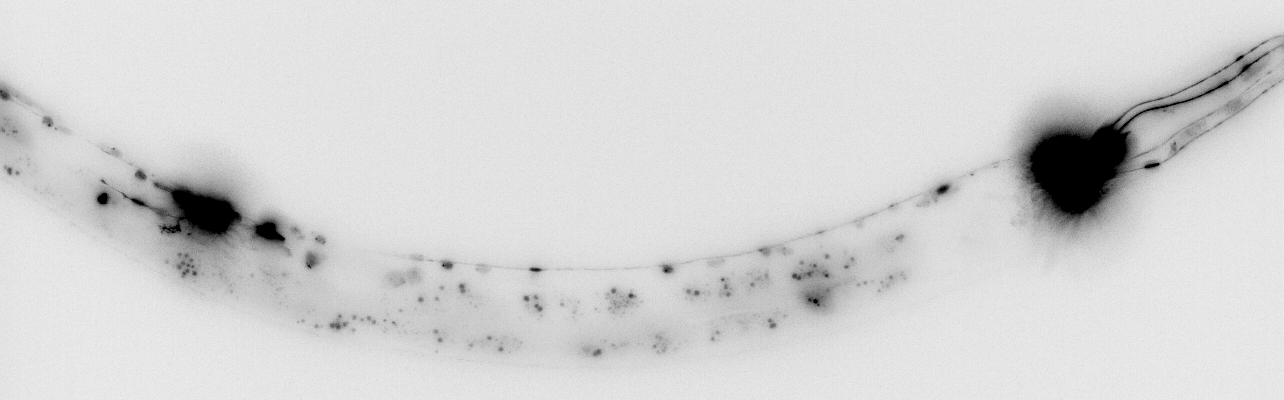

Supplement: Supplementary file 13 — Source Data Fig. 8 [file 44318_2024_49_MOESM13_ESM.zip › Figure 8/8G/Top/RFP.tif]

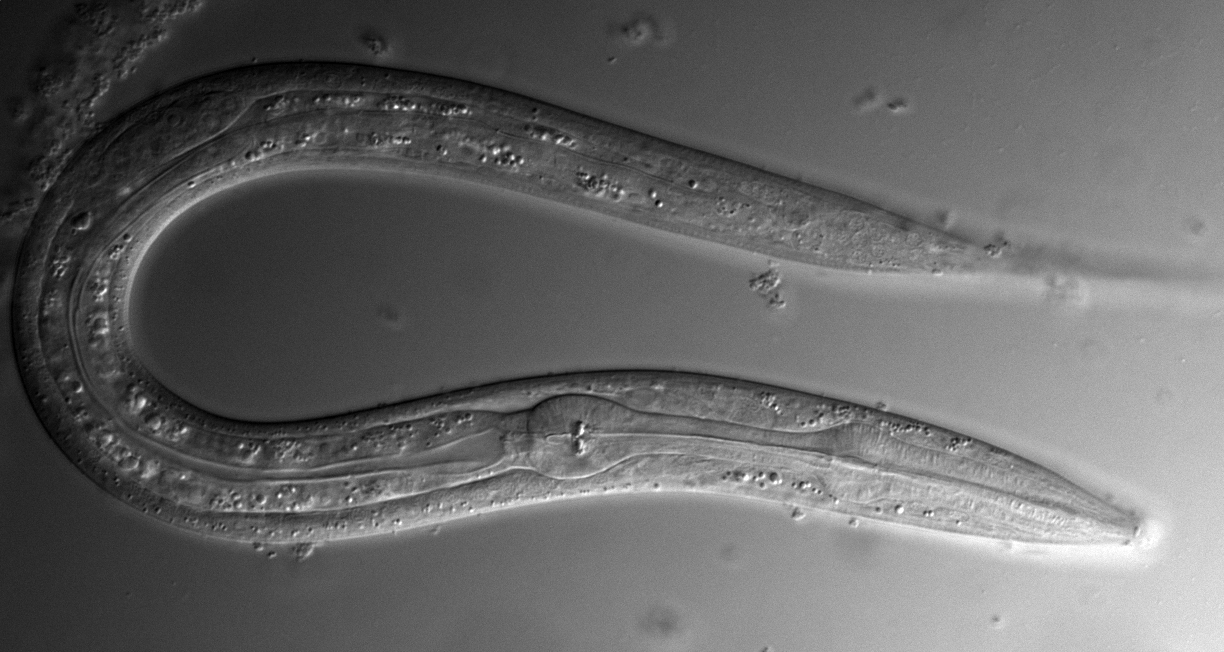

Supplement: Supplementary file 13 — Source Data Fig. 8 [file 44318_2024_49_MOESM13_ESM.zip › Figure 8/8G/Bottom/DIC.tif]

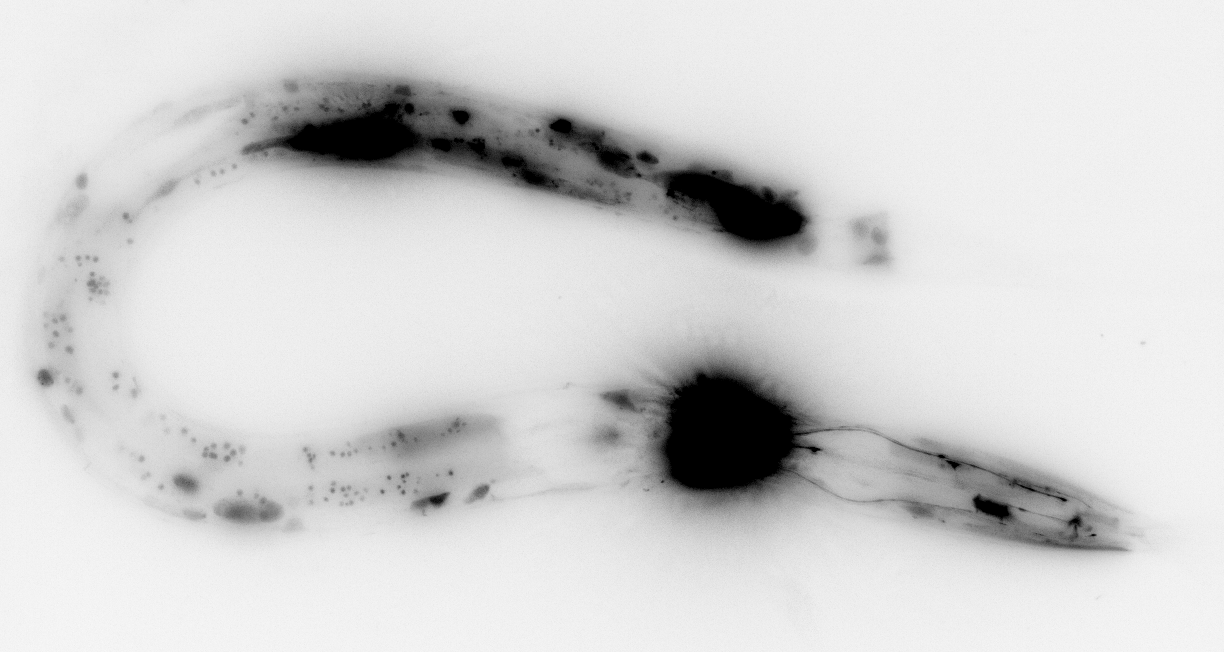

Supplement: Supplementary file 13 — Source Data Fig. 8 [file 44318_2024_49_MOESM13_ESM.zip › Figure 8/8G/Bottom/RFP.tif]

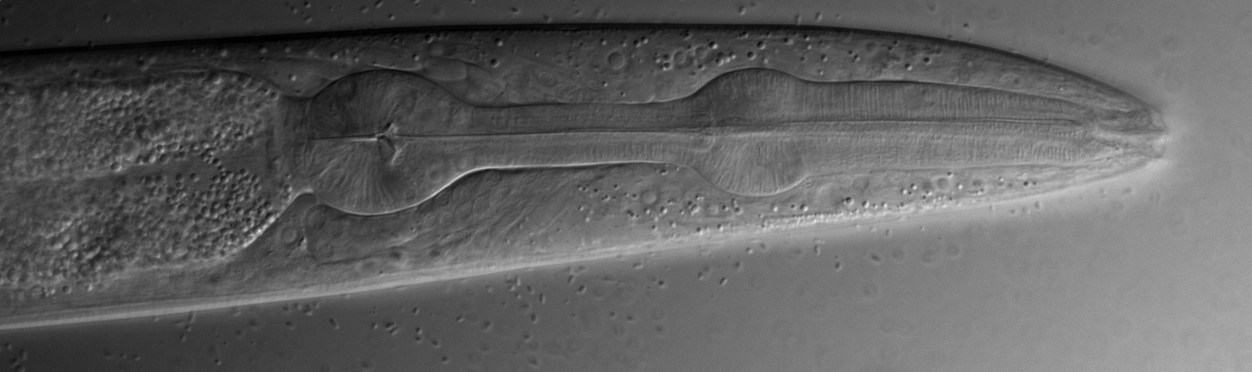

Supplement: Supplementary file 13 — Source Data Fig. 8 [file 44318_2024_49_MOESM13_ESM.zip › Figure 8/8A/left (wild type)/DIC.tif]

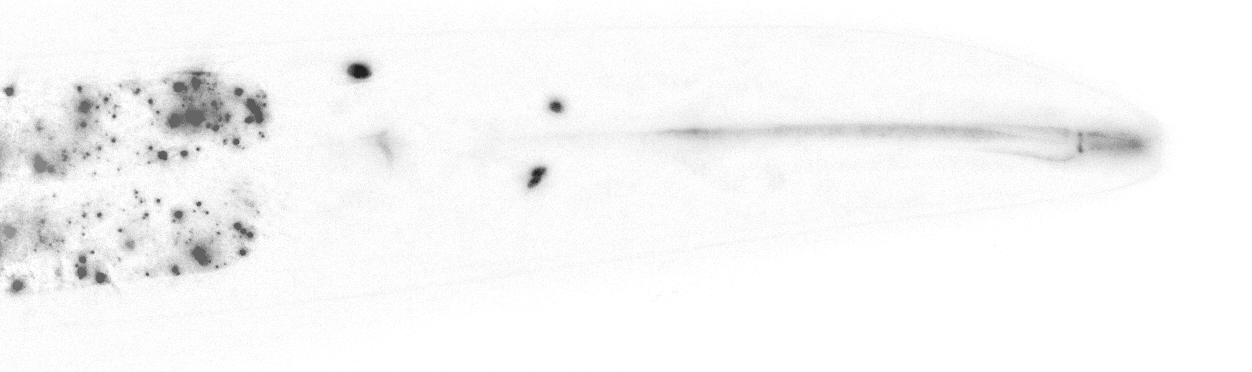

Supplement: Supplementary file 13 — Source Data Fig. 8 [file 44318_2024_49_MOESM13_ESM.zip › Figure 8/8A/left (wild type)/GFP.tif]

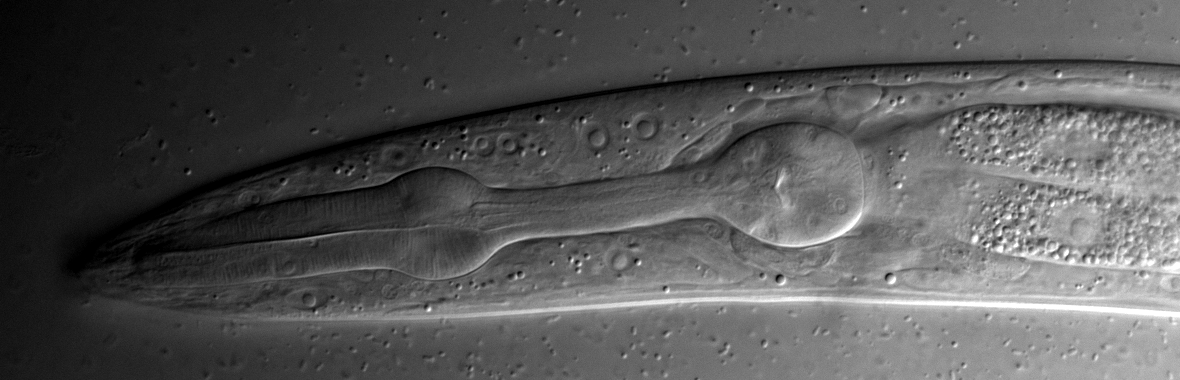

Supplement: Supplementary file 13 — Source Data Fig. 8 [file 44318_2024_49_MOESM13_ESM.zip › Figure 8/8A/right [unc-30(e191)]/DIC.tif]

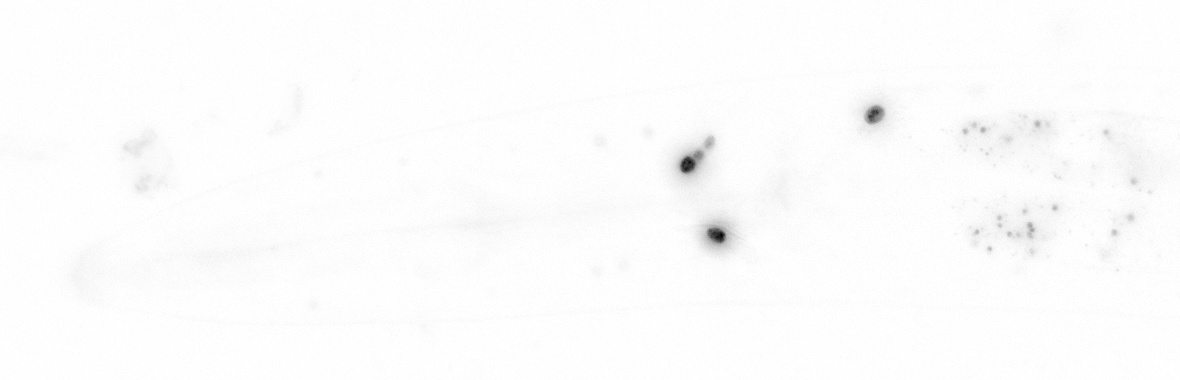

Supplement: Supplementary file 13 — Source Data Fig. 8 [file 44318_2024_49_MOESM13_ESM.zip › Figure 8/8A/right [unc-30(e191)]/GFP.tif]

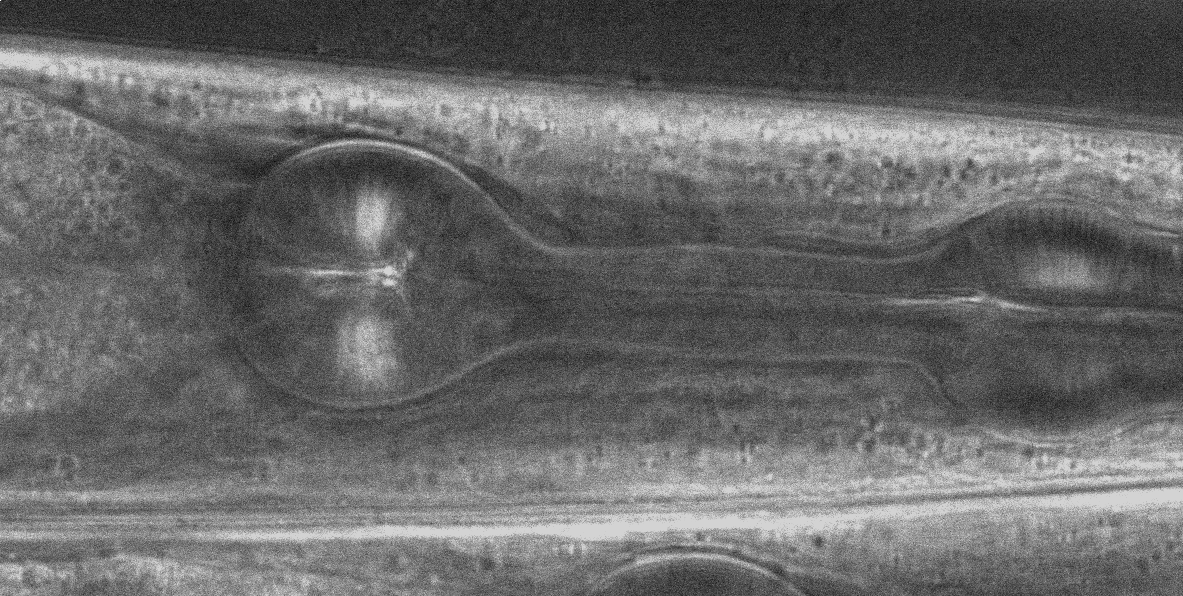

Supplement: Supplementary file 13 — Source Data Fig. 8 [file 44318_2024_49_MOESM13_ESM.zip › Figure 8/8C/Top (wild type)/DIC.tif]

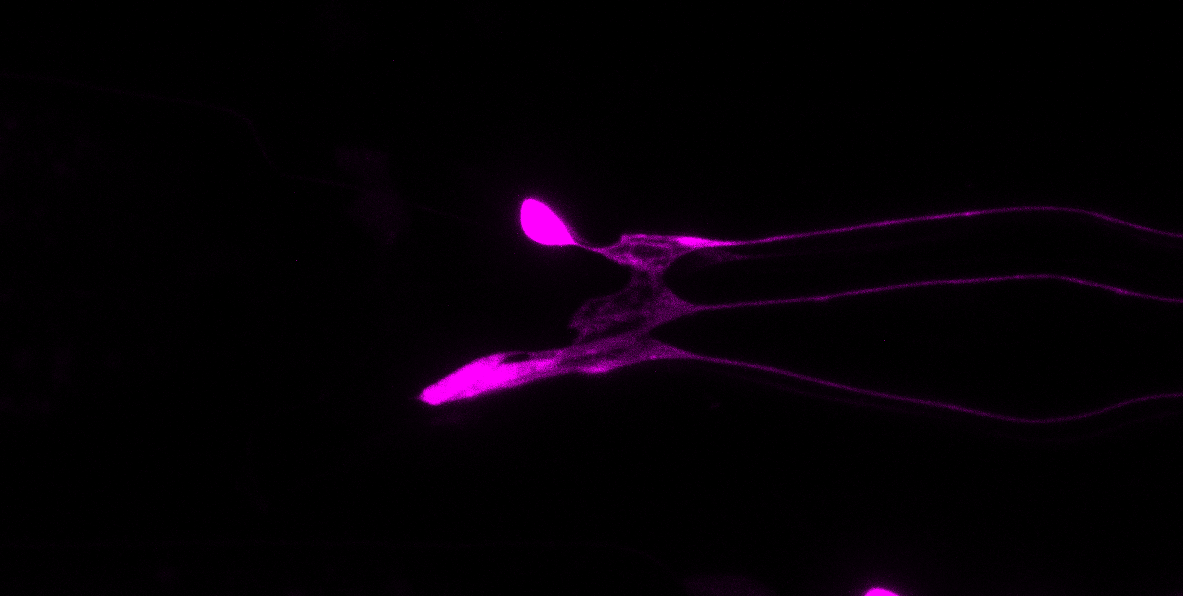

Supplement: Supplementary file 13 — Source Data Fig. 8 [file 44318_2024_49_MOESM13_ESM.zip › Figure 8/8C/Top (wild type)/RFP.tif]

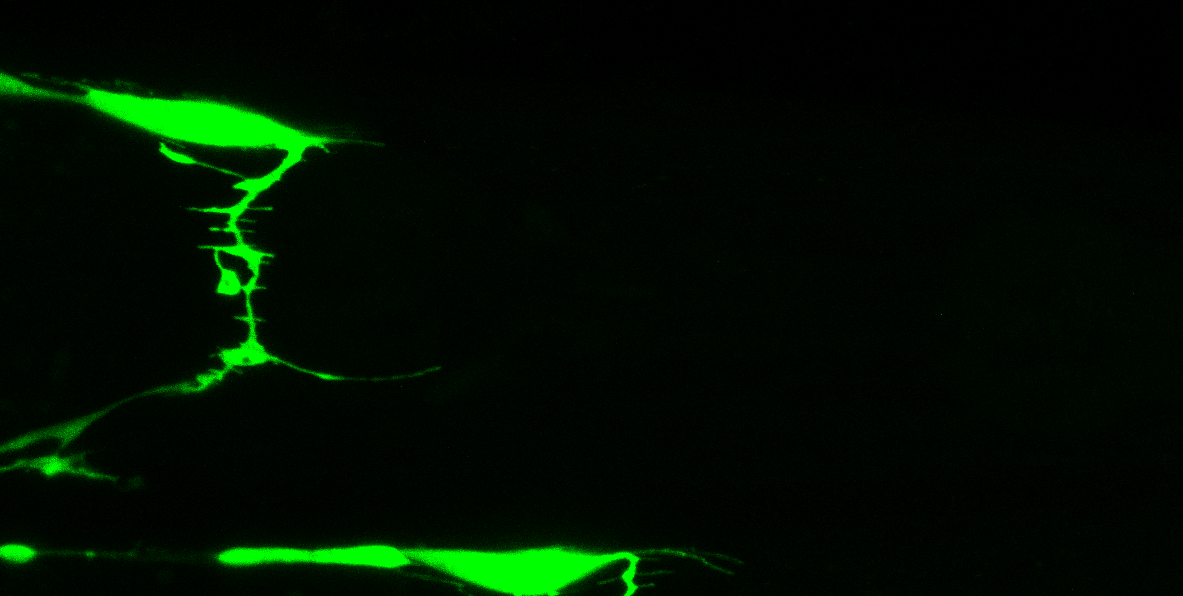

Supplement: Supplementary file 13 — Source Data Fig. 8 [file 44318_2024_49_MOESM13_ESM.zip › Figure 8/8C/Top (wild type)/GFP.tif]

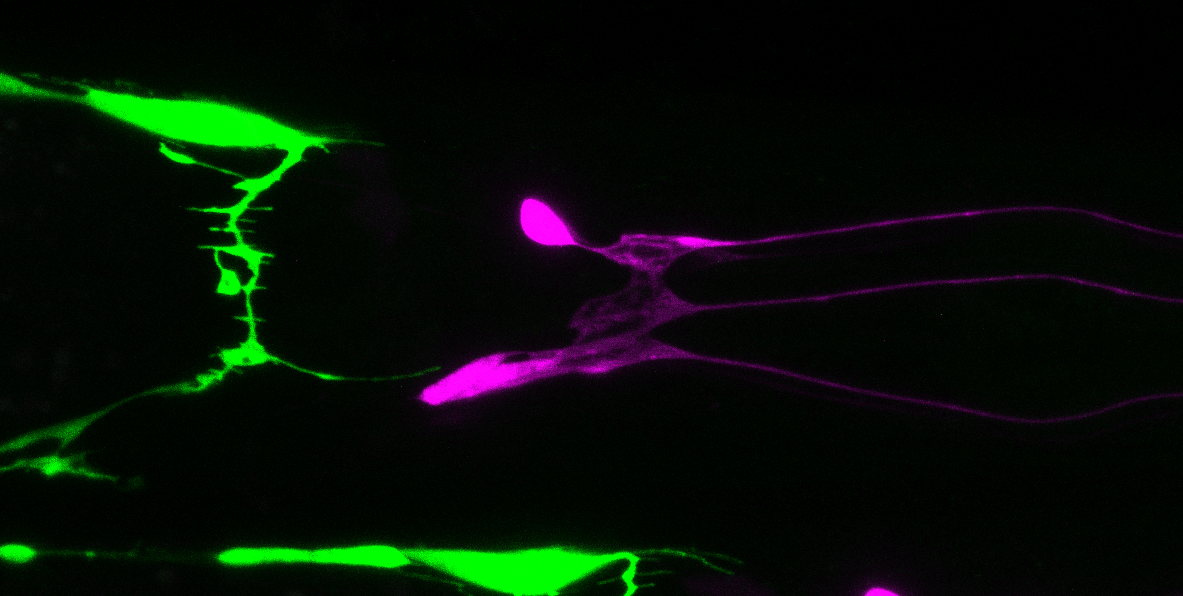

Supplement: Supplementary file 13 — Source Data Fig. 8 [file 44318_2024_49_MOESM13_ESM.zip › Figure 8/8C/Top (wild type)/merge.tif]

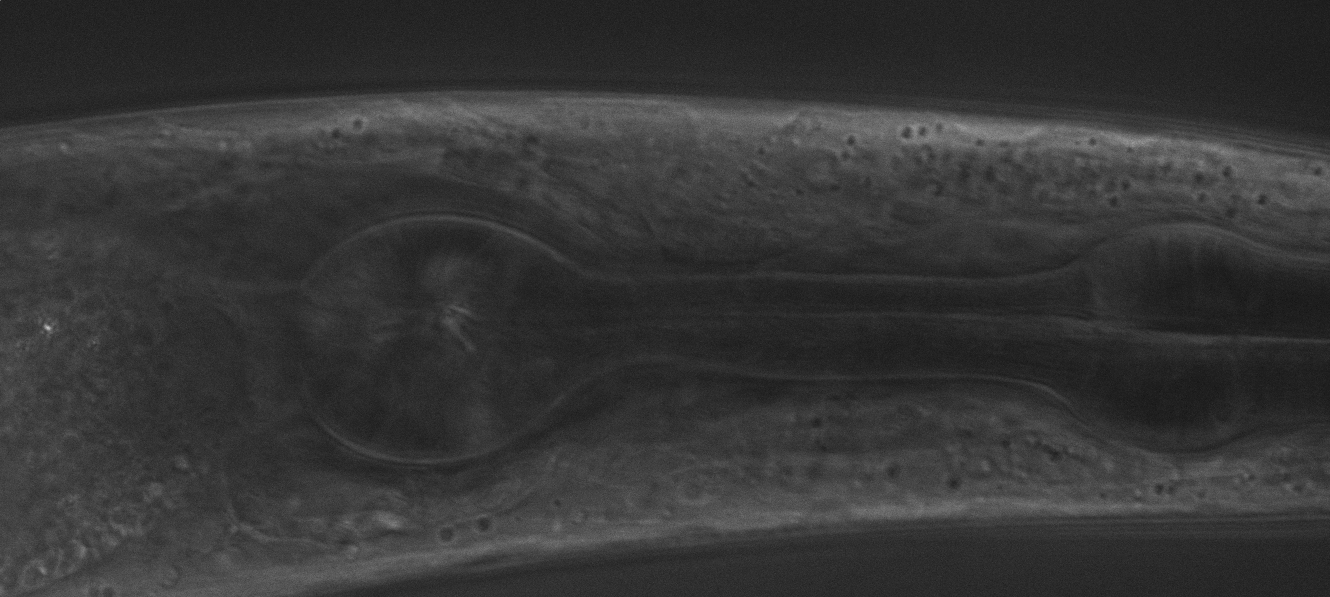

Supplement: Supplementary file 13 — Source Data Fig. 8 [file 44318_2024_49_MOESM13_ESM.zip › Figure 8/8C/Bottom [unc-30(e191)]/DIC.tif]

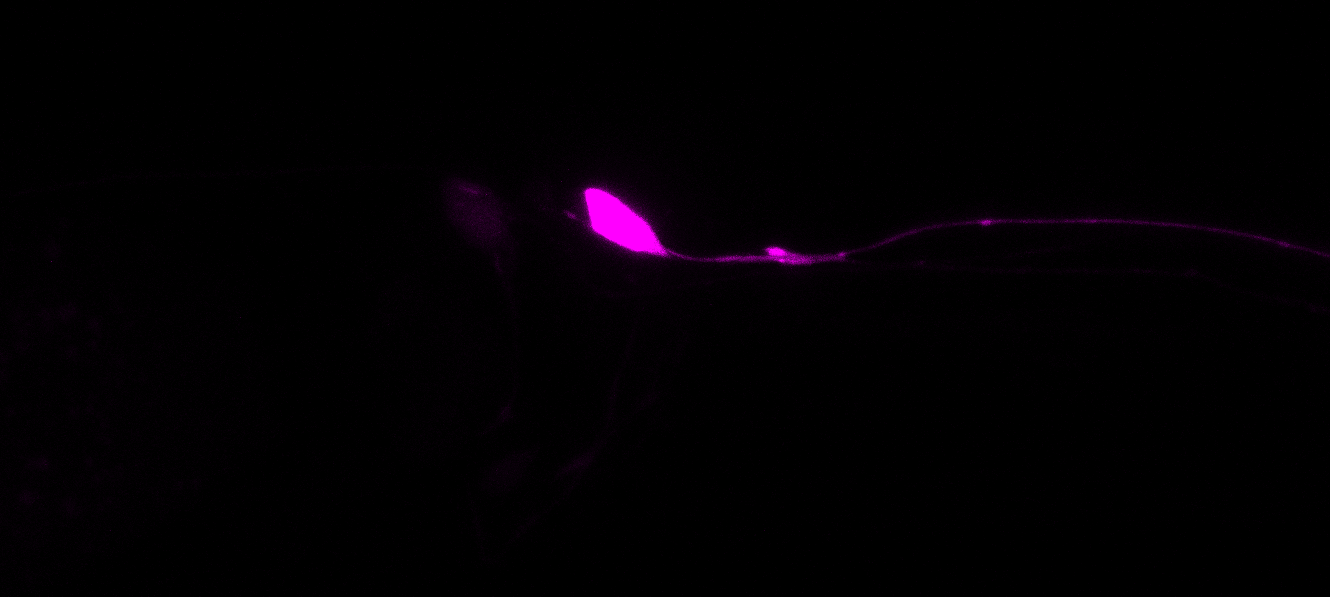

Supplement: Supplementary file 13 — Source Data Fig. 8 [file 44318_2024_49_MOESM13_ESM.zip › Figure 8/8C/Bottom [unc-30(e191)]/RFP.tif]

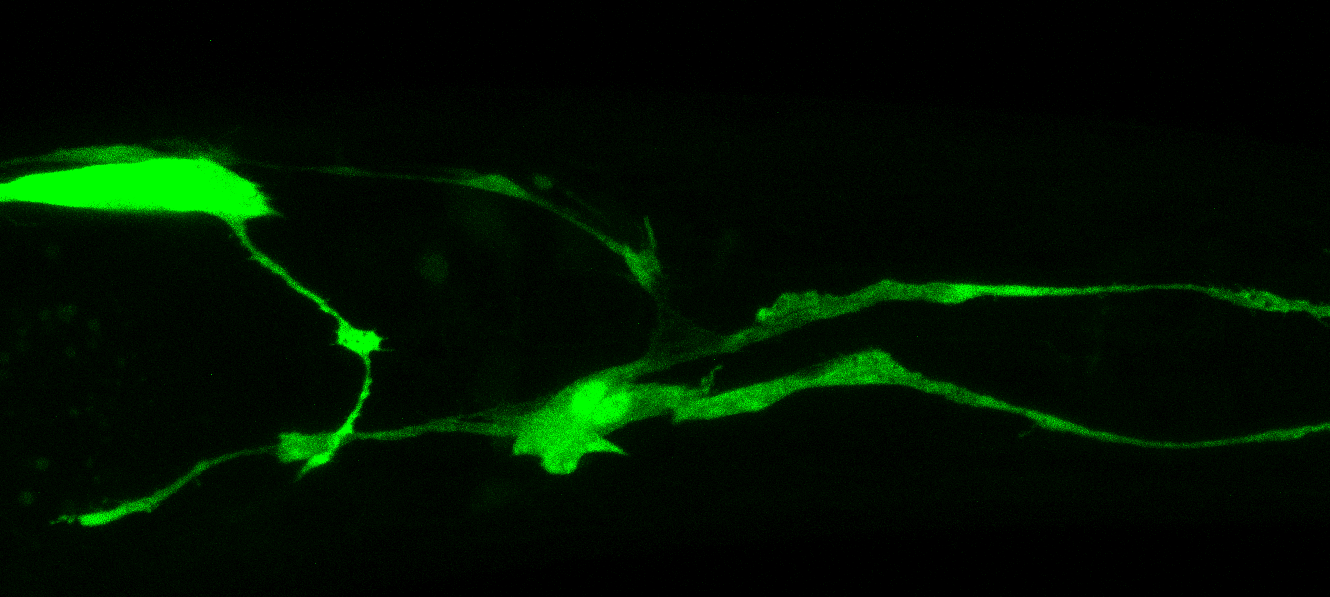

Supplement: Supplementary file 13 — Source Data Fig. 8 [file 44318_2024_49_MOESM13_ESM.zip › Figure 8/8C/Bottom [unc-30(e191)]/GFP.tif]

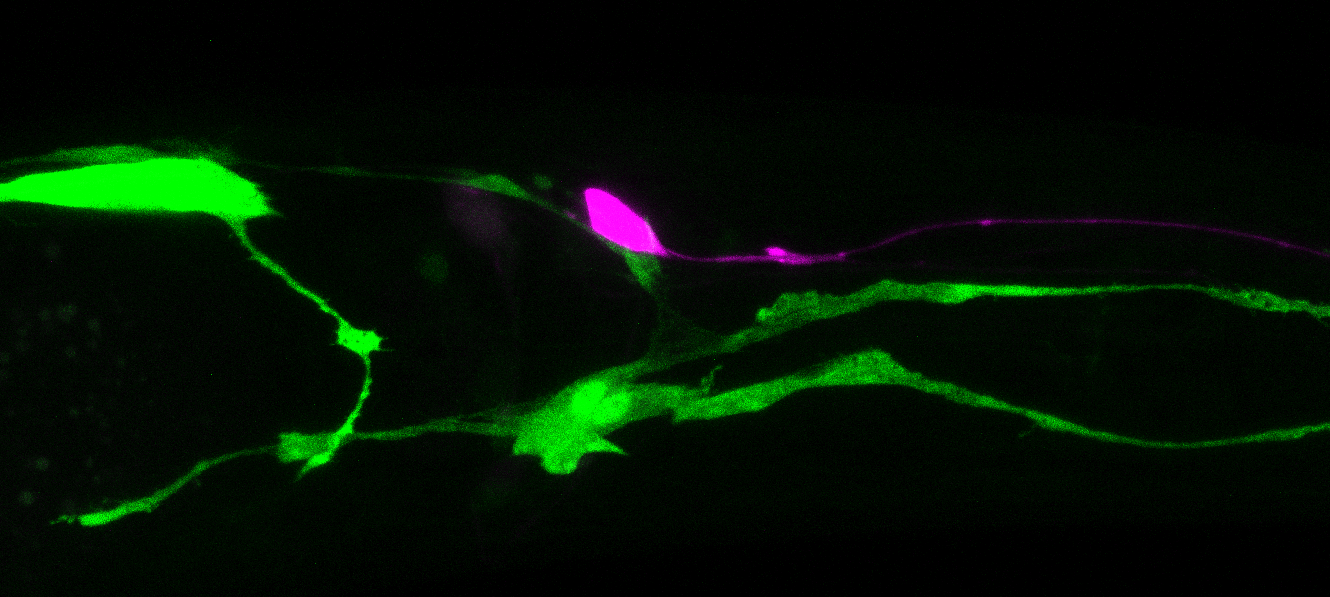

Supplement: Supplementary file 13 — Source Data Fig. 8 [file 44318_2024_49_MOESM13_ESM.zip › Figure 8/8C/Bottom [unc-30(e191)]/merge.tif]

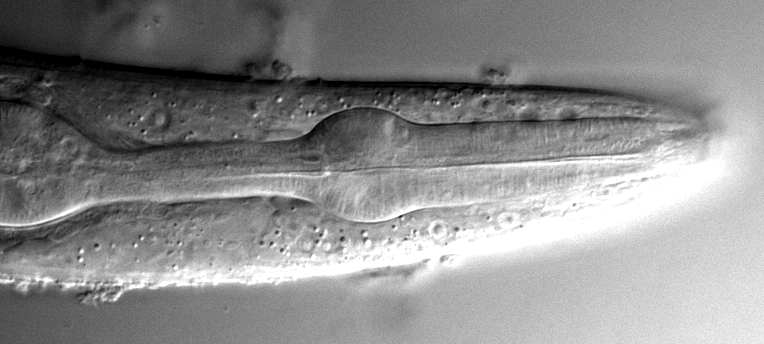

Supplement: Supplementary file 13 — Source Data Fig. 8 [file 44318_2024_49_MOESM13_ESM.zip › Figure 8/8E/left [unc-30(ns998)]/DIC.tif]

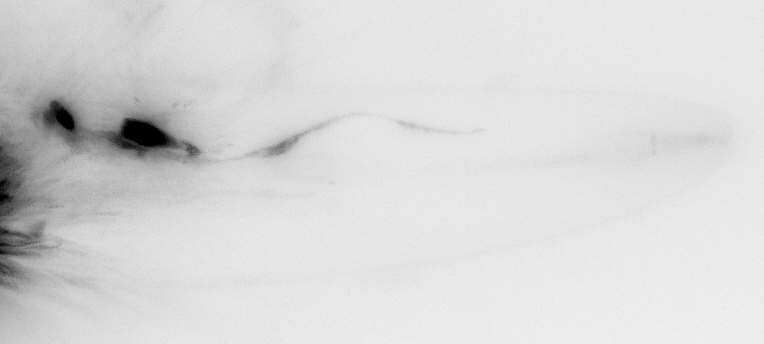

Supplement: Supplementary file 13 — Source Data Fig. 8 [file 44318_2024_49_MOESM13_ESM.zip › Figure 8/8E/left [unc-30(ns998)]/GFP.tif]

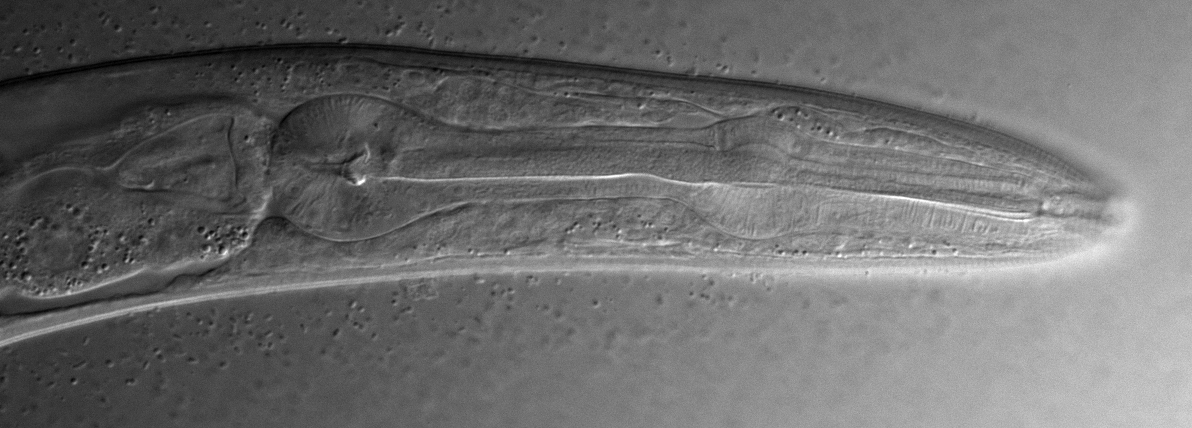

Supplement: Supplementary file 13 — Source Data Fig. 8 [file 44318_2024_49_MOESM13_ESM.zip › Figure 8/8E/right [unc-30(ns998) ; dmd-4(ot993)]/DIC.tif]

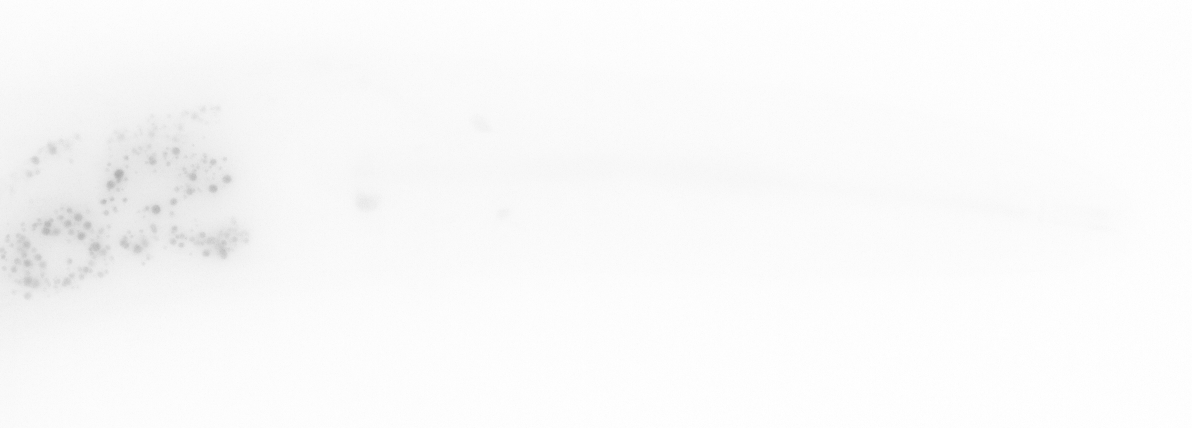

Supplement: Supplementary file 13 — Source Data Fig. 8 [file 44318_2024_49_MOESM13_ESM.zip › Figure 8/8E/right [unc-30(ns998) ; dmd-4(ot993)]/GFP.tif]

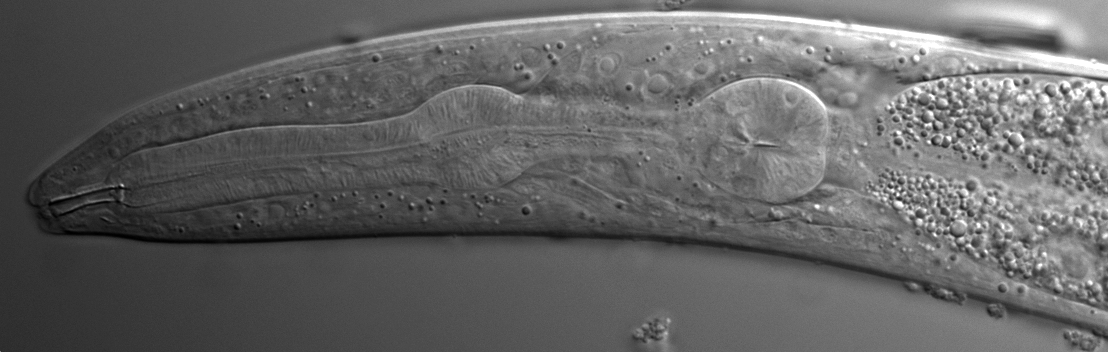

Supplement: Supplementary file 14 — Source Data Fig. 9 [file 44318_2024_49_MOESM14_ESM.zip › Figure 9/9B/DIC.tif]

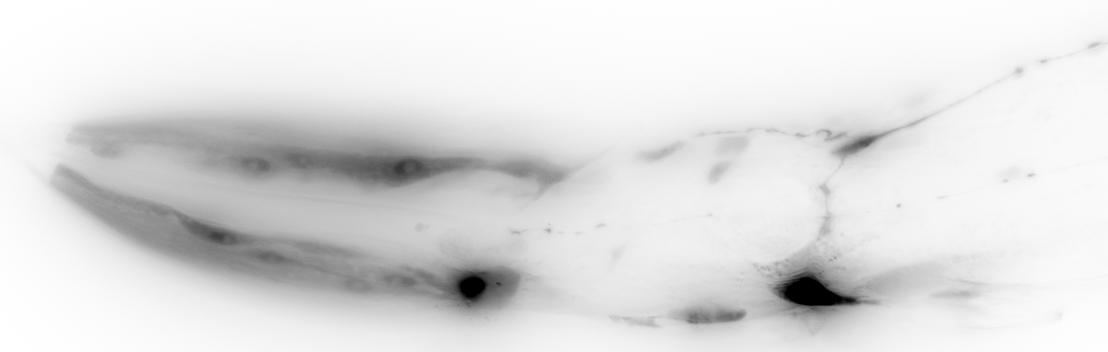

Supplement: Supplementary file 14 — Source Data Fig. 9 [file 44318_2024_49_MOESM14_ESM.zip › Figure 9/9B/GFP.tif]

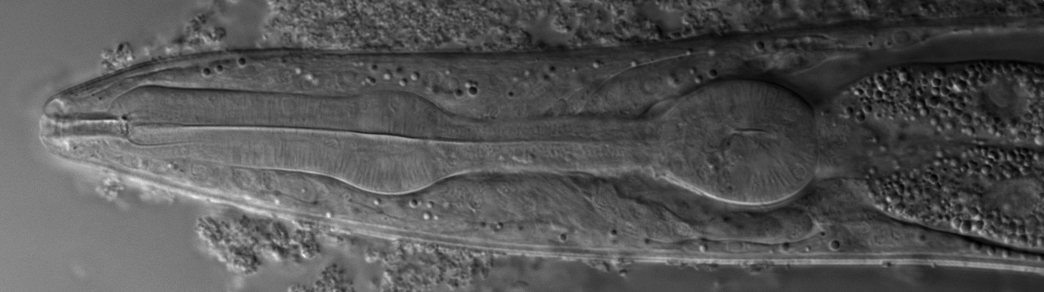

Supplement: Supplementary file 14 — Source Data Fig. 9 [file 44318_2024_49_MOESM14_ESM.zip › Figure 9/9C/DIC.tif]

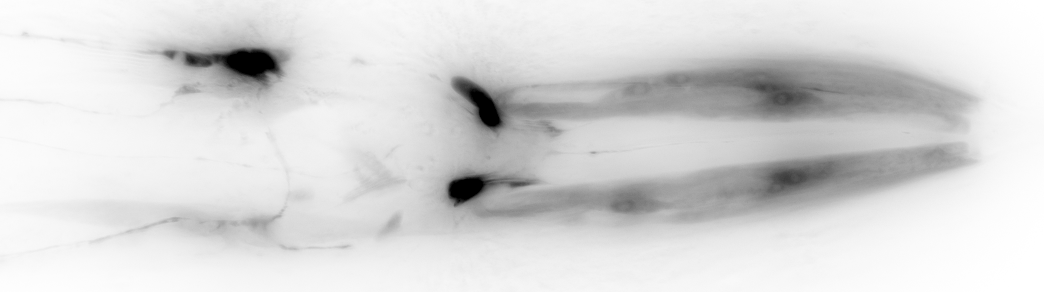

Supplement: Supplementary file 14 — Source Data Fig. 9 [file 44318_2024_49_MOESM14_ESM.zip › Figure 9/9C/GFP.tif]

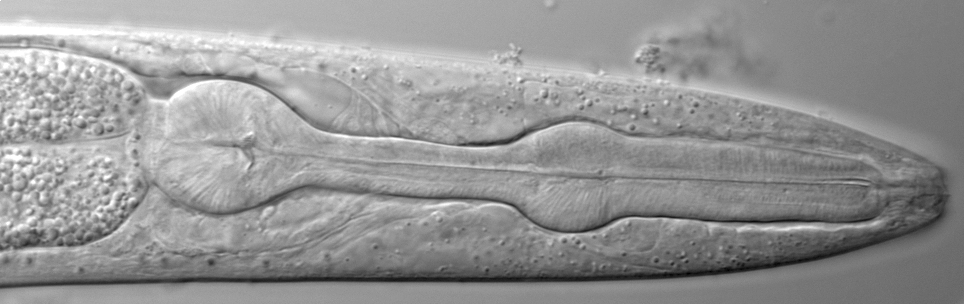

Supplement: Supplementary file 14 — Source Data Fig. 9 [file 44318_2024_49_MOESM14_ESM.zip › Figure 9/9A/DIC.tif]

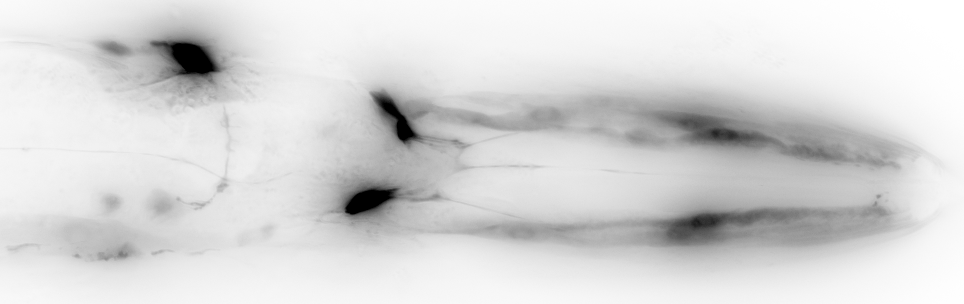

Supplement: Supplementary file 14 — Source Data Fig. 9 [file 44318_2024_49_MOESM14_ESM.zip › Figure 9/9A/GFP.tif]
