# Supplementary material for: LET-381/FoxF and its target UNC-30/Pitx2 specify and maintain the molecular identity of C. elegans mesodermal glia that regulate motor behavior
Source: EMBO J. 2024 Feb 15;43(6):4. doi: 10.1038/s44318-024-00049-w (PMC10943081; doi:10.1038/s44318-024-00049-w)
Supplement: Supplementary file 13 — Source Data Fig. 8 [file 44318_2024_49_MOESM13_ESM.zip › Figure 8/8C/README.rtf]

RFP is shown as magenta on the Figure panels because a green/magenta combination is better for colour-blind readers.
