# Supplementary material for: LET-381/FoxF and its target UNC-30/Pitx2 specify and maintain the molecular identity of C. elegans mesodermal glia that regulate motor behavior
Source: EMBO J. 2024 Feb 15;43(6):4. doi: 10.1038/s44318-024-00049-w (PMC10943081; doi:10.1038/s44318-024-00049-w)
Supplement: Supplementary file 16 — Expanded View Figures [file 44318_2024_49_MOESM16_ESM.pdf]

Expanded View Figures

Figure EV1. *let-381* motifs are required for endogenous GLR gene expression and *let-381* autoregulation in GLR glia.

(**A**) *let-381* motif identified in this study. (**B**) Motif of the *let-381* ortholog *foxf* from (Peterson et al, 1997). (**C**) *let-381* motif from (Narasimhan et al, 2015). Similarities between the three motifs are apparent. (**D**) Locations (distances from start codons) of *let-381* motifs of genes whose expression in GLR is downregulated in *let-381* mutants (either GLR-specific *let-381* RNAi and/or the *let-381* autoregulatory allele). (**E**) Minimal promoter *hlh-1prom1* was one of the promoters used in MEME to identify common motifs present in GLR glia genes. The *let-381* motif identified by MEME is highlighted in dark red. A *let-381* motif with slightly altered sequence (light red) was identified manually later and is required, together with the first motif, to control *hlh-1* expression in GLR glia. (**F**) Schematics showing details on endogenous *gfp*-based tags, location of *let-381* motifs and their mutation for *nep-2*, *pll-1*, *hlh-1* and *inx-18* genes. Red bars represent *let-381* motifs. Distance from ATG is indicated above each motif. Nucleotide changes for each motif mutation is shown below the motifs.

▶

© The Author(s)

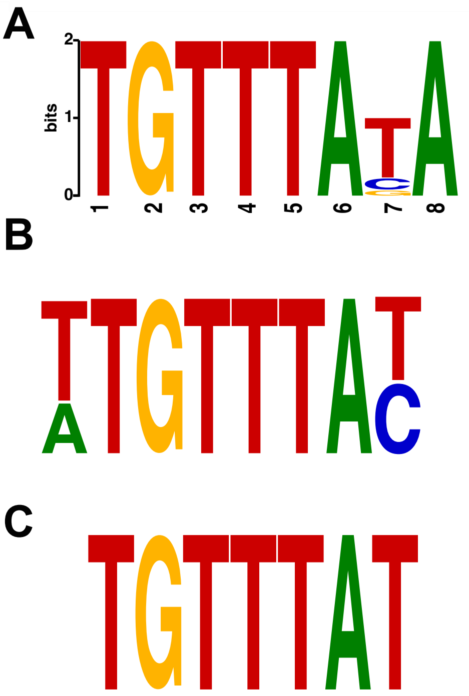

D

gene name	location of *let-381* motif(s) in base pairs from start codon
nep-2	–1133
gly-18	–147
hlh-1	+726 and +765
inx-18	+364
hot-5	–135 and –122
pll-1	–100
lgc-55	–3867
unc-46	–921
snf-11	–840
gbb-2	–388

E

***hlh-1p1* 270bp**

ggttataatgagcaccagatgaggctatttgttctgtacaggagcctgg
cggctaggctttttgcatgctattgattaataggggaatgcggggatgg
aaaaatcgaagtagtagggaaaggggagaagagaatttatgaaatgg
gtcatgggaatagtaaagggaggggggtgtttacattgtgcaaacttg
ggccttttaatccatttagtttatttcctttttcttttcaattcttgaaaaattc
gtagactgggttaacccgttg

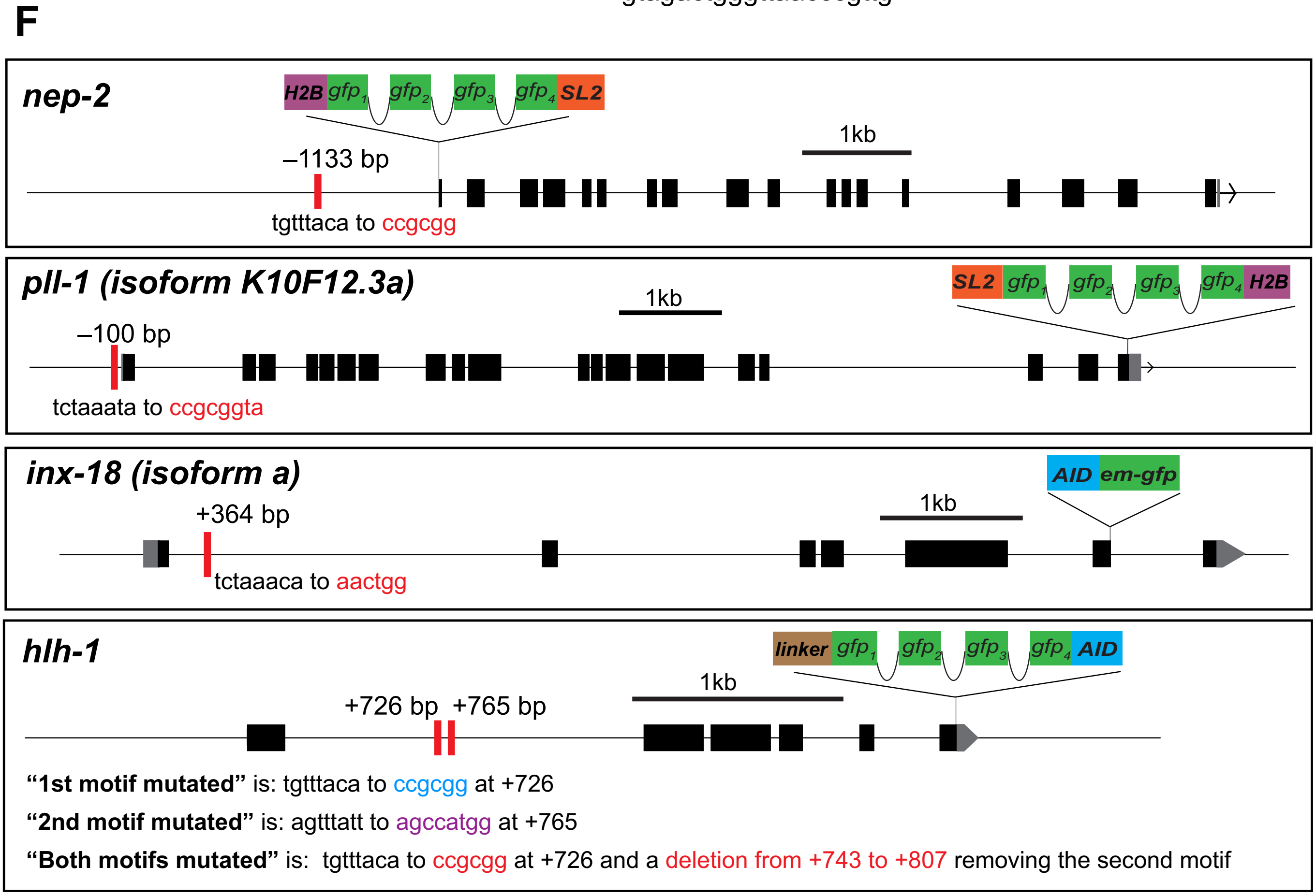

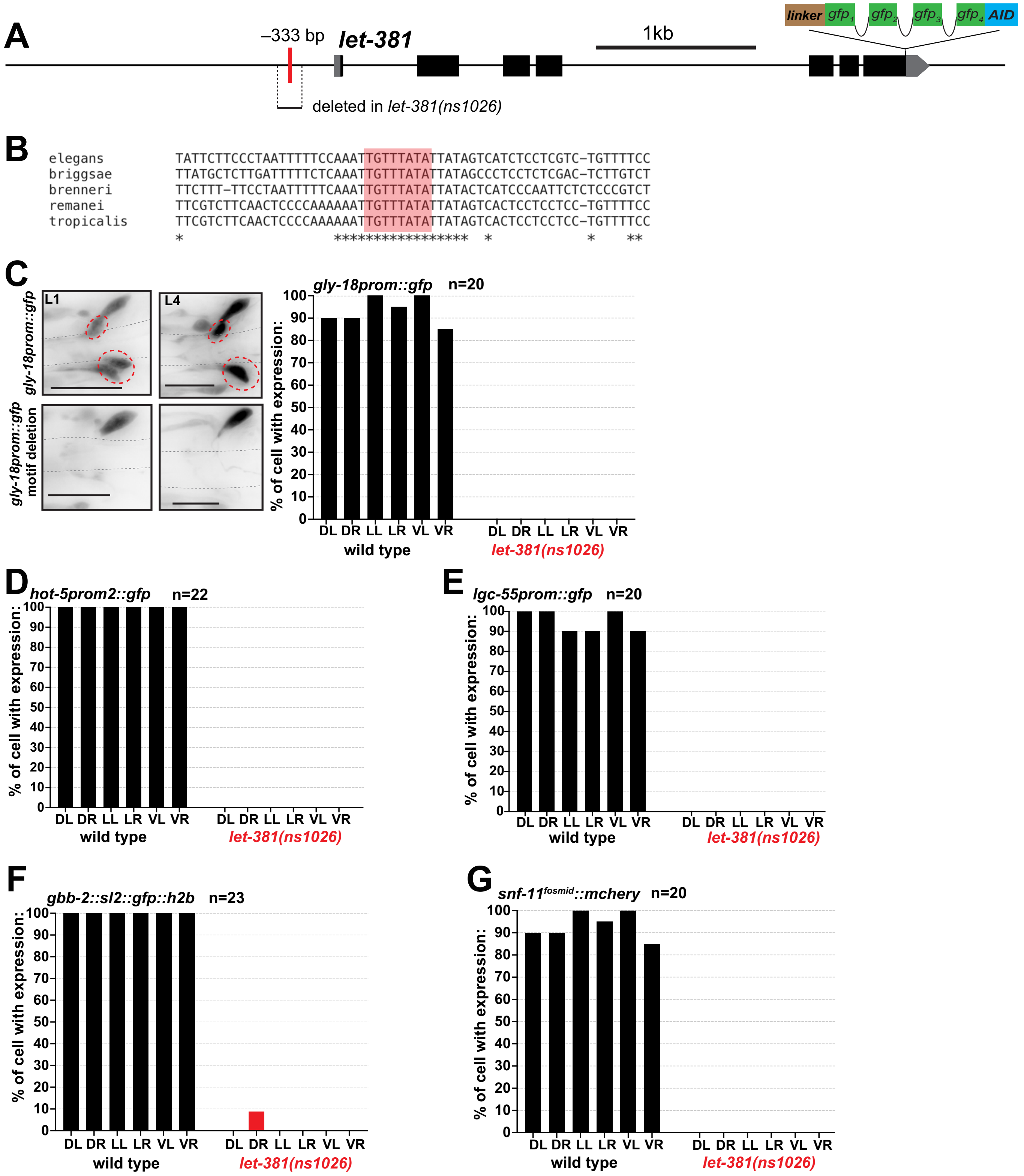
A
–333 bp
let-381
1kb
linker
gfp1
gfp2
gfp3
gfp4
AID
deleted in let-381(ns1026)
B
elegans TATTCTTCCCTAATTTTTCCAAATTGTTTATATTATAGTCATCTCCTCGTC-TGTTTTCC
briggsae TTATGCTCTTGATTTTTCTCAAATTGTTTATATTATAGCCCTCCTCTCGAC-TCTTGTCT
brenneri TTCTTT-TTCCTAATTTTTCAAATTGTTTATATTATACTCATCCCAATTCTCTCCCGTCT
remanei TTCGTCTTCAACTCCCCAAAAAATTGTTTATATTATAGTCACTCCTCCTCC-TGTTTTCC
tropicalis TTCGTCTTCAACTCCCCAAAAAATTGTTTATATTATAGTCACTCCTCCTCC-TGTTTTCC
C
gly-18prom::gfp
gly-18prom::gfp motif deletion
L1
L4
gly-18prom::gfp n=20
% of cell with expression:
DL DR LL LR VL VR
wild type
let-381(ns1026)
D
hot-5prom2::gfp n=22
E
lgc-55prom::gfp n=20
F
gbb-2::sl2::gfp::h2b n=23
G
snf-11fosmid::mchery n=20

© The Author(s)

◀ **Figure EV2. GLR gene expression is lost in *let-381* autoregulatory mutant animals.**

(**A**) Schematic showing the location of the *let-381* motif (red bar) in the *let-381* promoter region and region deleted in the *let-381(ns1026)* mutation. (**B**) Conservation of the *let-381* autoregulatory motif sequence (red box) is shown among five nematode species. Asterisks indicate conserved nucleotides. (**C–G**) Effect of *let-381* autoregulatory motif deletion, *let-381(ns1026)*, on expression of (**C**) *gly-18*, (**D**) *hot-5*, (**E**) *lgc-55*, (**F**) *gbb-2*, and (**G**) *snf-11* in GLR glia. Bar graphs show quantifications of gene expression at the L4 stage. For (**C**) animal images showing gene expression at L1 and L4 stages in wild-type and mutant backgrounds are shown on the left. Dashed red circles outline expression in GLR glia. Data information: Anterior is left, dorsal is up and scale bars are 10 μm for all animal images.

© The Author(s)

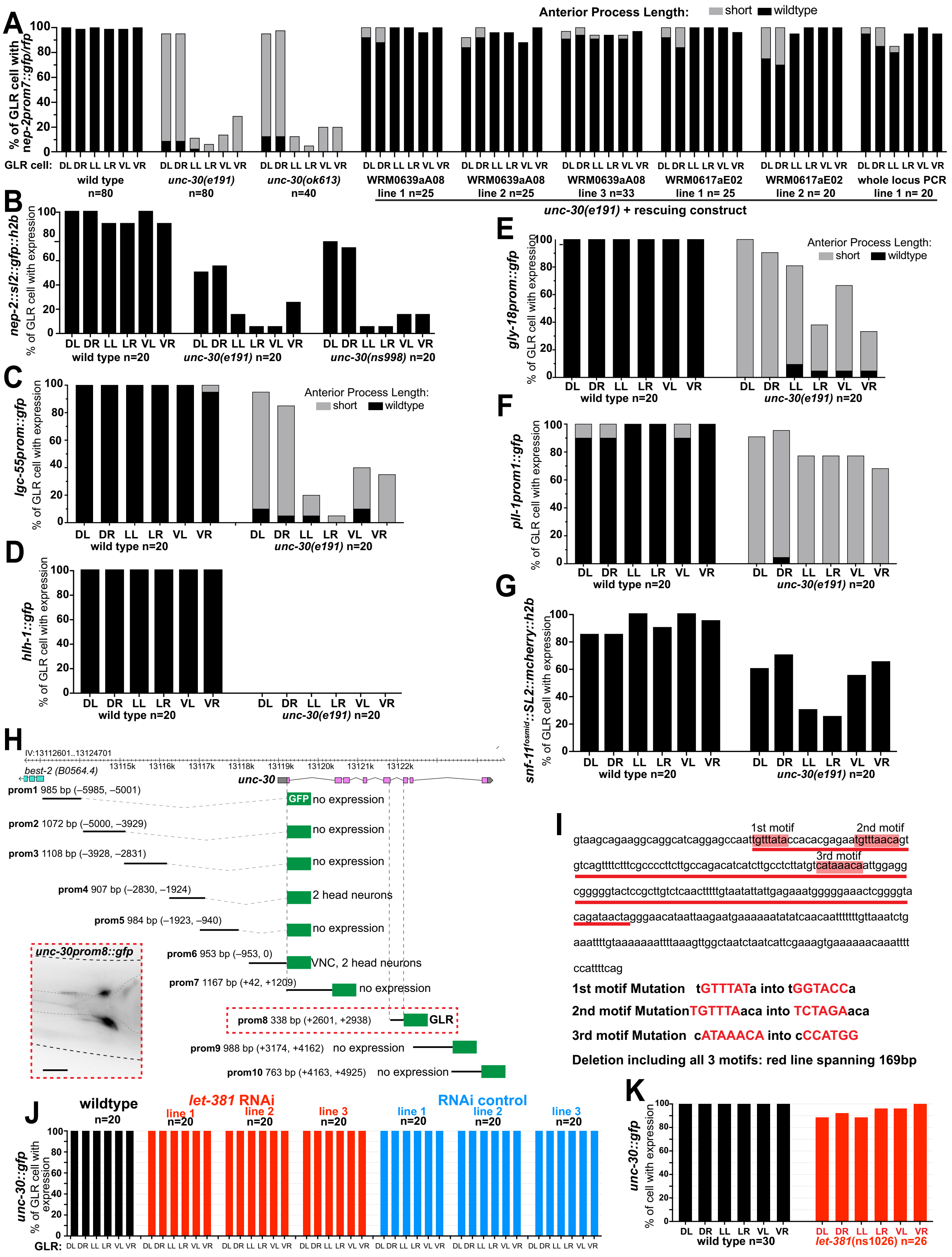

A
Anterior Process Length: short wildtype
% of GLR cell with nep-2prom7::gfp/rfp
GLR cell: DL DR LL LR VL VR
wild type n=80
unc-30(e191) n=80
unc-30(ok613) n=40
WRM0639aA08 line 1 n=25
WRM0639aA08 line 2 n=25
WRM0639aA08 line 3 n=33
WRM0617aE02 line 1 n= 25
WRM0617aE02 line 2 n= 20
whole locus PCR line 1 n= 20
unc-30(e191) + rescuing construct
B
nep-2::sl2::gfp::h2b
% of GLR cell with expression
wild type n=20
unc-30(e191) n=20
unc-30(ns998) n=20
C
lgc-55prom::gfp
% of GLR cell with expression
Anterior Process Length: short wildtype
wild type n=20
unc-30(e191) n=20
D
hlh-1::gfp
% of GLR cell with expression
wild type n=20
unc-30(e191) n=20
E
gly-18prom::gfp
% of GLR cell with expression
Anterior Process Length: short wildtype
wild type n=20
unc-30(e191) n=20
F
pll-1prom1::gfp
% of GLR cell with expression
wild type n=20
unc-30(e191) n=20
G
snf-11fosmid::SL2::mcherry::h2b
% of GLR cell with expression
wild type n=20
unc-30(e191) n=20
H
IV:13112601..13124701
best-2 (B0564.4)
unc-30
prom1 985 bp (–5985, –5001) GFP no expression
prom2 1072 bp (–5000, –3929) no expression
prom3 1108 bp (–3928, –2831) no expression
prom4 907 bp (–2830, –1924) 2 head neurons
prom5 984 bp (–1923, –940) no expression
prom6 953 bp (–953, 0) VNC, 2 head neurons
prom7 1167 bp (+42, +1209) no expression
prom8 338 bp (+2601, +2938) GLR
prom9 988 bp (+3174, +4162) no expression
prom10 763 bp (+4163, +4925) no expression
unc-30prom8::gfp
I
1st motif 2nd motif 3rd motif
gtaagcagaaggcaggcatcaggagccaattgtttataccacacgagaatgtttaacagt
gtcagttttcttcgcccccttcttgccagacatcatcttgcctcttatgtcataaacaattggagg
cgggggtactccgcttgtctcaactttttgtaatattattgagaaatgggggaaactcggggta
cagataactagggaacataattaagaatgaaaaaaatatatcaacaattttttttgttaaatctg
aaattttgtaaaaaaattttaaagttggctaatctaatcattcgaaagtgaaaaaacaaatttt
ccattttcag
1st motif Mutation tGTTTATa into tGGTACCa
2nd motif Mutation TGTTTAaca into TCTAGAaca
3rd motif Mutation cATAAACA into cCCATGG
Deletion including all 3 motifs: red line spanning 169bp
J
unc-30::gfp
% of GLR cell with expression
wildtype n=20
let-381 RNAi line 1 n=20 line 2 n=20 line 3 n=20
RNAi control line 1 n=20 line 2 n=20 line 3 n=20
GLR: DL DR LL LR VL VR
K
unc-30::gfp
% of cell with expression
wild type n=30
let-381(ns1026) n=26

 © The Author(s)

◀ **Figure EV3. Effect of *unc-30* on GLR gene expression.**

(**A**) Transgenic constructs containing different fosmid clones (WRM) or PCR amplicons carrying wild-type copies of UNC-30 can rescue the effect of *unc-30(e191)* on GLR gene expression and anterior process length. (**B-G**) Effect of *unc-30* mutation on expression of different genes in GLR glia. Expression of (**E**) *gly-18*, (**F**) *pll-1* and (**G**) *snf-11* is affected at a lesser extent compared to (**B**) *nep-2*, (**C**) *lgc-55* and (**D**) *hlh-1*. (**H**) Cis-regulatory dissection analysis of *unc-30*. The fifth intron (prom8) of *unc-30* is sufficient to drive expression in GLR glia. (**I**) Three *let-381* motifs are found in the fifth intron of *unc-30* (red boxes). Details on *let-381* motif mutation alleles are shown below the DNA sequence. (**J**, **K**) Endogenous *unc-30::gfp* expression is not affected by postembryonic *let-381* knockdown either (**J**) by GLR-specific RNAi or (**K**) in the GLR-specific *let-381* autoregulatory motif deletion allele *let-381(ns1026)*. Data information: Anterior is left, dorsal is up and scale bars are 10 µm for all animal images.

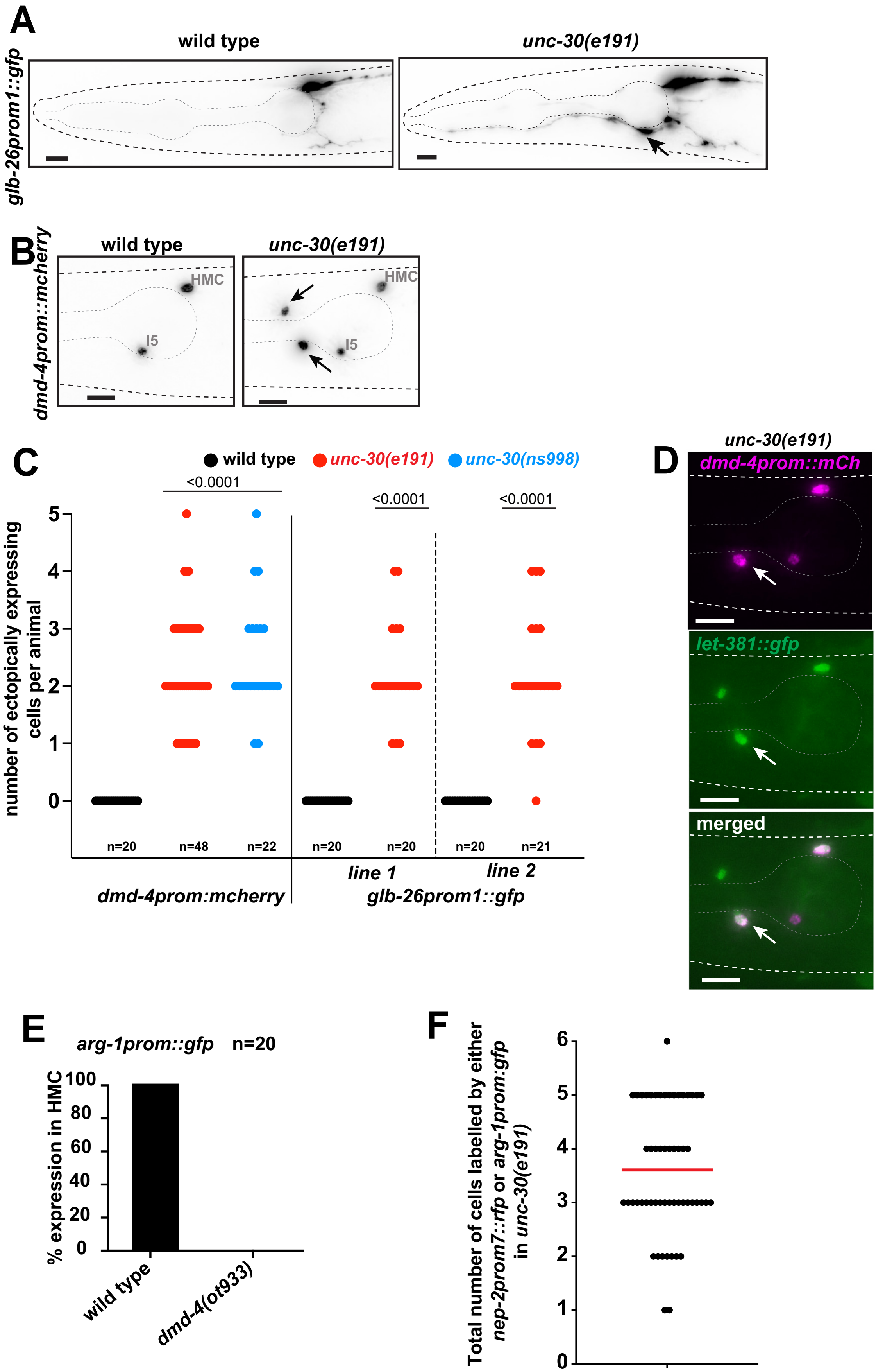
A
wild type
unc-30(e191)
glb-26prom1::gfp
B
wild type
unc-30(e191)
dmd-4prom::mcherry
HMC
I5
C
wild type
unc-30(e191)
unc-30(ns998)
<0.0001
number of ectopically expressing cells per animal
n=20
n=48
n=22
n=20
n=20
n=20
n=21
line 1
line 2
dmd-4prom:mcherry
glb-26prom1::gfp
D
unc-30(e191)
dmd-4prom::mCh
let-381::gfp
merged
E
arg-1prom::gfp n=20
% expression in HMC
wild type
dmd-4(ot933)
F
Total number of cells labelled by either nep-2prom7::rfp or arg-1prom:gfp in unc-30(e191)

 © The Author(s)

Figure EV4. ***unc-30*** **represses HMC gene expression in GLR glia.**

(**A**) Fluorescence images showing expression of *glb-26prom::gfp* in wild type and *unc-30(e191)* mutants. (**B**) Fluorescence images showing expression of *dmd-4prom::mCherry* in wild type and *unc-30(e191)* mutants. Arrows point to ectopic expression in *unc-30(e191)* mutants. (**C**) Quantification of ectopic expression of the two HMC reporters shown in (**A**) and (**B**) in *unc-30* mutant backgrounds. (**D**) Cells ectopically expressing (white arrow) the HMC reporter *dmd-4prom::mCherry* (magenta) always co-express *let-381::gfp* (green). (**E**) Expression of *arg-1prom::gfp* is lost in HMC in *dmd-4(ot933)* mutants. (**F**) Total number of GLR glia cells expressing either the GLR glia-specific *nep-2prom7::rfp* or the HMC-specific *arg-1prom::gfp* in *unc-30(e191)* mutants. Data information: unpaired *t* test used for statistical analysis in (**C**). Anterior is left, dorsal is up and scale bars are 10 μm for all animal images.

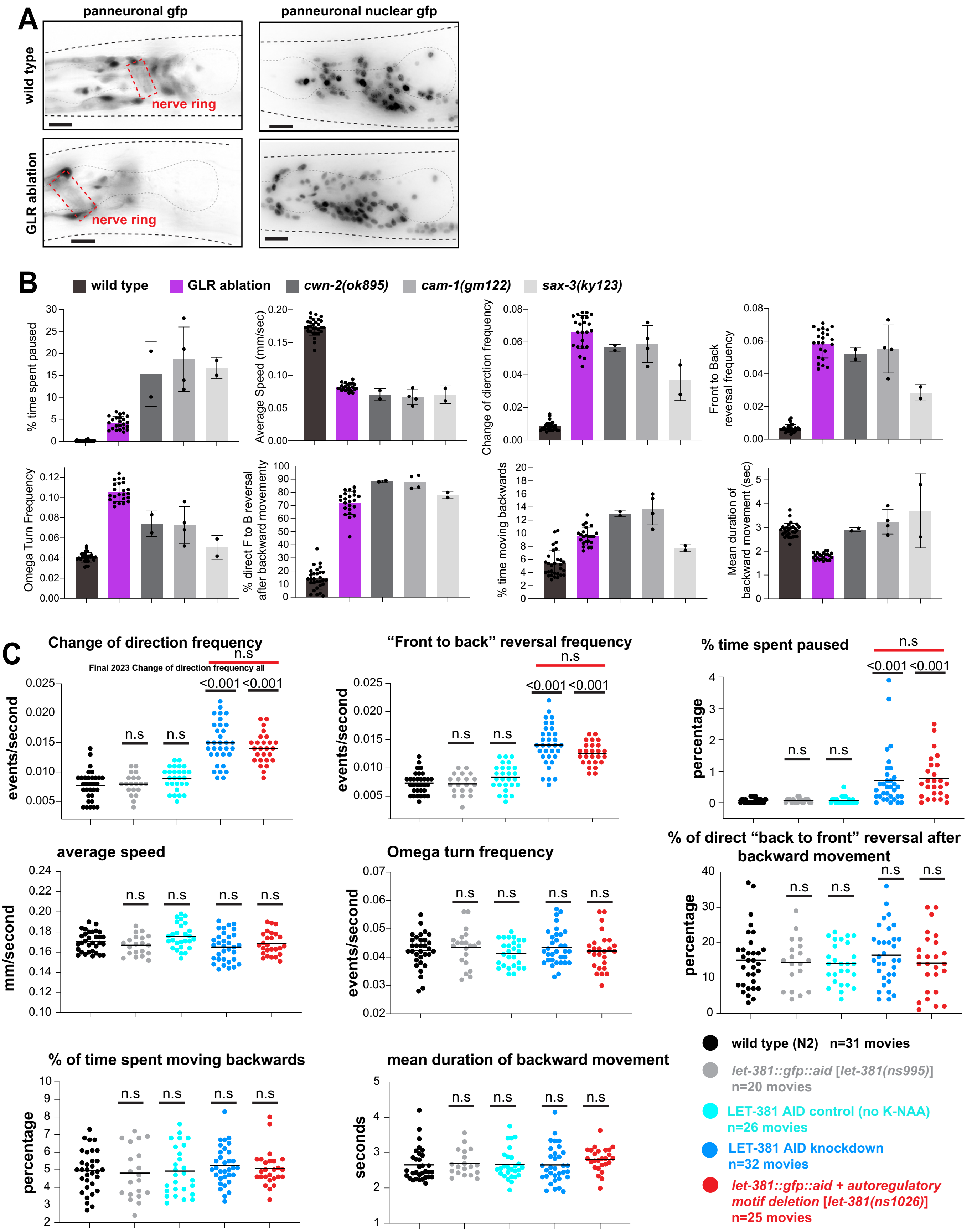
A
panneuronal gfp
panneuronal nuclear gfp
wild type
GLR ablation
nerve ring
B
wild type
GLR ablation
cwn-2(ok895)
cam-1(gm122)
sax-3(ky123)
% time spent paused
Average Speed (mm/sec)
Change of dierction frequency
Front to Back reversal frequency
Omega Turn Frequency
% direct F to B reversal after backward movemeny
% time moving backwards
Mean duration of backward movement (sec)
C
Change of direction frequency
Final 2023 Change of direction frequency all
"Front to back" reversal frequency
% time spent paused
average speed
Omega turn frequency
% of direct "back to front" reversal after backward movement
% of time spent moving backwards
mean duration of backward movement
events/second
percentage
mm/second
seconds
n.s
<0.001
wild type (N2) n=31 movies
let-381::gfp::aid [let-381(ns995)] n=20 movies
LET-381 AID control (no K-NAA) n=26 movies
LET-381 AID knockdown n=32 movies
let-381::gfp::aid + autoregulatory motif deletion [let-381(ns1026)] n=25 movies

 © The Author(s)

Figure EV5. Locomotion defects of GLR-ablated animals could partially be due to anteriorly displaced nerve ring.

(**A**) In wild-type animals (top row), axons of the nerve ring (dashed red box) are located between the two pharyngeal bulbs. In GLR-ablated animals (bottom row) the nerve ring is anteriorly displaced, located on top of the anterior pharynx bulb. As evidenced in the images on the right, not only the axonal projections, but also neuronal cell bodies (panneuronal nuclear gfp) are anteriorly displaced. Panneuronal gfp = *unc-119prom::gfp*, panneuronal nuclear gfp = *rab-3prom1::nls::yfp*. (**B**) *cwn-2(ok895)*, *cam-1(gm122)*, *sax-3(ky123)* mutants with anteriorly displaced nerve rings exhibit locomotion defects to the same direction, although of different magnitude as the GLR glia-ablated animals. (**C**) Auxin (K-NAA) dependent LET-381::AID knockdown results in similar defects in the same locomotion parameters as the *let-381(ns1026)* autoregulatory mutation. Genotypes are: wild-type N2 (black), *let-381(ns995)* control (gray), LET-381::AID knockdown [*let-381(ns995);nsIs879 (nep-2prom7::TIR1)*] exposed to K-NAA auxin (dark blue), LET-381::AID control [*let-381(ns995) ; nsIs879 (nep-2prom7::TIR1)*] not exposed to K-NAA auxin (light blue) and *let-381(ns1026)* autoregulatory mutation (red). Data information: in (**B**) wild type $n = 29$ movies, GLR ablation $n = 23$ movies, *cwn-2(ok895)* $n = 2$ movies, *cam-1(gm122)* $n = 4$ movies, *sax-3(ky123)* $n = 2$ movies. Bar height indicates average (center of error bars) and error bars show standard deviation in (**B**). Unpaired t test used for statistical analysis in (**C**); controls (gray and light blue) were compared to wild type (black). LET-38::AID knockdown (dark blue) was compared to its control group (light blue) and *let-381(ns1026)* was compared to its control (gray). No statistically significant differences were observed between the LET-381::AID knockdown and *let-381(ns1026)* as indicated by the red line on the top of the three upper diagrams. Anterior is left, dorsal is up and scale bars are 10 µm for all animal images.

© The Author(s)
